# Critical assessment of protein intrinsic disorder prediction

**DOI:** 10.1038/s41592-021-01117-3

**Published:** 2021-04-19

**Authors:** Marco Necci, Damiano Piovesan, CAID Predictors, Md Tamjidul Hoque, Ian Walsh, Sumaiya Iqbal, Michele Vendruscolo, Pietro Sormanni, Chen Wang, Daniele Raimondi, Ronesh Sharma, Yaoqi Zhou, Thomas Litfin, Oxana Valerianovna Galzitskaya, Michail Yu. Lobanov, Wim Vranken, Björn Wallner, Claudio Mirabello, Nawar Malhis, Zsuzsanna Dosztányi, Gábor Erdős, Bálint Mészáros, Jianzhao Gao, Kui Wang, Gang Hu, Zhonghua Wu, Alok Sharma, Jack Hanson, Kuldip Paliwal, Isabelle Callebaut, Tristan Bitard-Feildel, Gabriele Orlando, Zhenling Peng, Jinbo Xu, Sheng Wang, David T. Jones, Domenico Cozzetto, Fanchi Meng, Jing Yan, Jörg Gsponer, Jianlin Cheng, Tianqi Wu, Lukasz Kurgan, DisProt Curators, Vasilis J. Promponas, Stella Tamana, Cristina Marino-Buslje, Elizabeth Martínez-Pérez, Anastasia Chasapi, Christos Ouzounis, A. Keith Dunker, Andrey V. Kajava, Jeremy Y. Leclercq, Burcu Aykac-Fas, Matteo Lambrughi, Emiliano Maiani, Elena Papaleo, Lucia Beatriz Chemes, Lucía Álvarez, Nicolás S. González-Foutel, Valentin Iglesias, Jordi Pujols, Salvador Ventura, Nicolás Palopoli, Guillermo Ignacio Benítez, Gustavo Parisi, Claudio Bassot, Arne Elofsson, Sudha Govindarajan, John Lamb, Marco Salvatore, András Hatos, Alexander Miguel Monzon, Martina Bevilacqua, Ivan Mičetić, Giovanni Minervini, Lisanna Paladin, Federica Quaglia, Emanuela Leonardi, Norman Davey, Tamas Horvath, Orsolya Panna Kovacs, Nikoletta Murvai, Rita Pancsa, Eva Schad, Beata Szabo, Agnes Tantos, Sandra Macedo-Ribeiro, Jose Antonio Manso, Pedro José Barbosa Pereira, Radoslav Davidović, Nevena Veljkovic, Borbála Hajdu-Soltész, Mátyás Pajkos, Tamás Szaniszló, Mainak Guharoy, Tamas Lazar, Mauricio Macossay-Castillo, Peter Tompa, Silvio C. E. Tosatto

**Affiliations:** 1 Department of Biomedical Sciences, University of Padua, Padua, Italy; 2 Computer Science, University of New Orleans, New Orleans, LA USA; 3 Bioprocessing Technology Institute, Agency for Science, Technology and Research, Singapore, Singapore; 4 Center for the Development of Therapeutics and Stanley Center for Psychiatric Research, Broad Institute of MIT and Harvard, Cambridge, MA USA; 5 Centre for Misfolding Diseases, Department of Chemistry, University of Cambridge, Cambridge, UK; 6 Department of Medicine, Columbia University, New York, NY USA; 7 ESAT-STADIUS, KU Leuven, Leuven, Belgium; 8 Fiji National University, Suva, Fiji; 9 Institute for Glycomics and School of Information and Communication Technology, Griffith University, Southport, Queensland Australia; 10 Institute of Protein Research, Russian Academy of Sciences, Pushchino, Russia; 11 Institute of Theoretical and Experimental Biophysics, Russian Academy of Sciences, Pushchino, Russia; 12 Interuniversity Institute of Bioinformatics in Brussels, Vrije Universiteit Brussel, Brussels, Belgium; 13 Division of Bioinformatics, Department of Physics, Chemistry, and Biology, Linköping University, Linköping, Sweden; 14 Michael Smith Laboratories, University of British Columbia, Vancouver, British Columbia Canada; 15 MTA-ELTE Lendulet Bioinformatics Research Group, Department of Biochemistry, Eötvös Loránd University, Budapest, Hungary; 16 Structural and Computational Biology Unit, European Molecular Biology Laboratory, Heidelberg, Germany; 17 School of Mathematical Sciences and LPMC, Nankai University, Tianjin, China; 18 School of Statistics and Data Science, LPMC and KLMDASR, Nankai University, Tianjin, China; 19 RIKEN Center for Integrative Medical Sciences, Yokohama, Japan; 20 Griffith University, Brisbane, Queensland Australia; 21 School of Engineering and Physics, University of the South Pacific, Suva, Fiji; 22 Signal Processing Laboratory, School of Engineering and Built Environment, Griffith University, Brisbane, Queensland Australia; 23 Institut de Minéralogie, de Physique des Matériaux et de Cosmochimie, Sorbonne Université, Muséum National d’Histoire Naturelle, Paris, France; 24 Switch Laboratorium, VIB-KU Leuven, Leuven, Belgium; 25 Center for Applied Mathematics, Tianjin University, Tianjin, China; 26 Toyota Technological Institute at Chicago, Chicago, IL USA; 27 University College London, London, UK; 28 Department of Electrical and Computer Engineering, University of Alberta, Edmonton, Alberta Canada; 29 Department of Electrical Engineering and Computer Science, University of Missouri, Columbia, SC USA; 30 Department of Computer Science, Virginia Commonwealth University, Richmond, VA USA; 31 Bioinformatics Research Laboratory, Department of Biological Sciences, University of Cyprus, Nicosia, Cyprus; 32 Bioinformatics Unit, Fundación Instituto Leloir, Avda, Patricias Argentinas, Buenos Aires, Argentina; 33 Chemical Process & Energy Resources Institute, Centre for Research & Technology Hellas, Thessalonica, Greece; 34 Center for Computational Biology and Bioinformatics, Indiana University School of Medicine, Indianapolis, IN USA; 35 Centre de Recherche en Biologie cellulaire de Montpellier, University of Montpellier, Montpellier, France; 36 Danish Cancer Society Research Center, Copenhagen, Denmark; 37 Consejo Nacional de Investigaciones Científicas y Técnicas, Instituto de Investigaciones Biotecnológicas, Universidad Nacional de San Martín, San Martín, Buenos Aires, Argentina; 38 Departament de Bioquimica i Biologia Molecular and Institut de Biotecnologia i Biomedicina, Universitat Autònoma de Barcelona, Bellaterra, Spain; 39 Departamento de Ciencia y Tecnología, Universidad Nacional de Quilmes – CONICET, Bernal, Buenos Aires, Argentina; 40 Department of Biochemistry and Biophysics and Science for Life Laboratory, Stockholm University, Solna, Sweden; 41 Department of Biology, Section of Computational and RNA Biology, Copenhagen University, Copenhagen, Denmark; 42 Department of Woman and Child Health, Univerisity of Padova – Pediatric Research Institute, Città della Speranza, Padova, Italy; 43 Division of Cancer Biology, The Institute of Cancer Research, Chelsea, London, UK; 44 Institute of Enzymology, Research Centre for Natural Sciences, Budapest, Hungary; 45 Instituto de Biologia Molecular e Celular and Instituto de Investigação e Inovação em Saúde, Universidade do Porto, Porto, Portugal; 46 Vinča Institute of Nuclear Sciences – National Institute of thе Republic of Serbia, University of Belgrade, Belgrade, Serbia; 47 MTA-ELTE Lendület Bioinformatics Research Group, Department of Biochemistry, Eötvös Loránd University, Budapest, Hungary; 48 VIB-VUB Center for Structural Biology, Brussels, Belgium; 49 Structural Biology Brussels, Vrije Universiteit Brussel, Brussels, Belgium

**Keywords:** Computational platforms and environments, Software, Proteins, Protein structure predictions, Machine learning

## Abstract

Intrinsically disordered proteins, defying the traditional protein structure–function paradigm, are a challenge to study experimentally. Because a large part of our knowledge rests on computational predictions, it is crucial that their accuracy is high. The Critical Assessment of protein Intrinsic Disorder prediction (CAID) experiment was established as a community-based blind test to determine the state of the art in prediction of intrinsically disordered regions and the subset of residues involved in binding. A total of 43 methods were evaluated on a dataset of 646 proteins from DisProt. The best methods use deep learning techniques and notably outperform physicochemical methods. The top disorder predictor has *F*_max_ = 0.483 on the full dataset and *F*_max_ = 0.792 following filtering out of bona fide structured regions. Disordered binding regions remain hard to predict, with *F*_max_ = 0.231. Interestingly, computing times among methods can vary by up to four orders of magnitude.

## Abstract (web summary)

Results are presented from the first Critical Assessment of protein Intrinsic Disorder prediction (CAID) experiment, a community-based blind test to determine the state of the art in predicting intrinsically disordered regions in proteins.

## Main

Intrinsically disordered proteins (IDPs) and regions (IDRs) that do not adopt a fixed, three-dimensional fold under physiological conditions are now well recognized in structural biology^1^. The last two decades have seen an increase in evidence for the involvement of IDPs and IDRs in a variety of essential biological processes^2,3^ and molecular functions that complement those of globular domains^4,5^. Their involvement in diseases such as Alzheimer’s^6^, Parkinson’s^7^ and cancer^8^ also makes them promising targets for drug discovery^9,10^. Despite their importance, IDPs/IDRs are historically understudied due to the difficulties in direct measurement of their dynamic behavior and because some of them tend to be disordered only under specific conditions, such as pH, presence of post-translational modifications, localization and binding—that is, their structural disorder is context dependent^11^. Experimental methods used to detect intrinsic structural disorder (ID) include X-ray crystallography, nuclear magnetic resonance spectroscopy (NMR), small-angle X-ray scattering, circular dichroism and Förster resonance energy transfer^12–15^. Each technique provides a unique point of view on the phenomenon of ID, and different types of experimental evidence give researchers insights into the functional mechanisms of IDPs, such as flexibility, folding-upon-binding and conformational heterogeneity.

An accumulation of experimental evidence has corroborated the early notion that ID can be inferred from sequence features^16^. Dozens of ID prediction methods based on different principles and computing techniques have been published^17^, including VSL2B^18^, DisEMBL^19^, DISOPRED^20^, IUPred^21^ and Espritz^22^. Both predicted and experimentally derived coordinates of IDRs and annotations related to their function are stored in a variety of dedicated databases: DisProt^23^, MobiDB^24^, IDEAL^25^, DIBS^26^ and MFIB^27^ each focus on particular aspects of the ID spectrum. More recently, IDR annotations are also included in some core data resources including InterPro^28^, UniProt^29^ and PDBe^30^.

Intrinsic structural disorder binding predictions are widely used, but an assessment of these predictors has never been systematically performed and is badly needed. In this report, we describe the first edition of CAID, a biennial experiment inspired by the critical assessment of protein structure prediction (CASP) for the benchmarking of ID and binding predictors on a community-curated dataset of 646 novel proteins obtained from DisProt^23^. CAID is expected to set a new quality standard in the field.

## Results

**Fig. 1:**
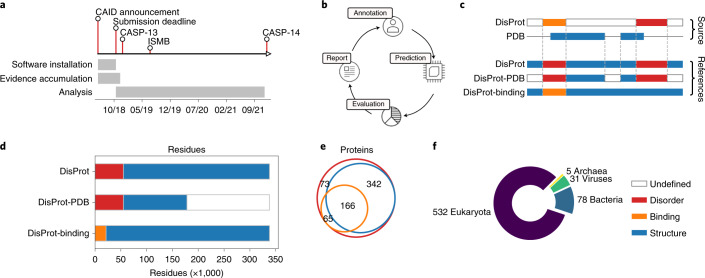
CAID dashboard. **a**, CAID timeline: phases of CAID from June 2018 to the present. The initial results were presented and discussed at the conferences Intelligent Systems for Molecular Biology (ISMB) and CASP. **b**, CAID process: iterative process of the CAID experiment in four phases. (1) Annotation: any process that produces unpublished annotation of IDR coordinates; in this edition, annotation refers to the DisProt round of annotation. (2) Prediction: annotations are used to build references with which we test predictors. (3) Evaluation: predictions are evaluated. (4) Report: a report of the evaluation is produced and published in peer-reviewed journals and on a web page that allows the reader to browse the evaluation of all CAID editions. **c**, Residue classification strategy for the DisProt and DisProt-PDB references. **d**, Number of residues for each class in different references. **e**, Number of proteins for each set of annotations that they contain. **f**, Number of proteins in each taxon.

CAID was organized as follows (Fig. 1a). Participants submitted their implemented prediction software to the assessors and provided support to install and test them on the MobiDB servers. The assessors ran the packages and generated predictions for a set of proteins for which disorder annotations were not previously available. Given a protein sequence, the task of an ID predictor is to assign a score to each residue for its propensity of being intrinsically disordered at any stage of the protein’s life. In CAID, we evaluated the accuracy of the prediction methods as well as software runtimes, which directly impact their suitability for large-scale analyses.

Structural properties of proteins can be studied by a number of different experimental techniques, giving direct or indirect evidence of disorder. Different techniques are biased in different ways. For example, IDRs inferred from missing residues in X-ray experiments are generally shorter because longer, noncrystallizable IDRs are either excised when preparing the construct or are detrimental to crystallization. At the other end of the spectrum is circular dichroism, which can detect the absence of fixed structure in the full protein but does not provide any information about IDR coordinates. IDR annotations are more reliable when confirmed by multiple lines of independent and different experimental evidence.

In this first round of CAID, we selected the DisProt database as the reference for structural disorder because it provides a large number of manually curated disorder annotations at the protein level, with the majority of residues annotated with more than one experiment^23^. DisProt annotates IDRs of at least ten residues likely to be associated with a biological function and excludes short loops connecting secondary structure elements. DisProt also contains protein–protein interaction interfaces falling into disordered regions, used as a separate dataset (DisProt-binding).

Ideally DisProt annotations would be complete—that is, each protein would be annotated with all disordered (or binding) regions present under physiological conditions. If this were true, we could simply consider all residues to be structured (that is negatives) when not annotated as disordered (that is, positives). Since not all IDRs are yet in DisProt, we created the DisProt-Protein Database (-PDB) dataset, where negatives are restricted to PDB Observed residues (Fig. 1c). This dataset is more conservative but can be considered more reliable as it excludes ‘uncertain’ residues that have neither structural nor disorder annotation. Compared to DisProt, DisProt-PDB is more similar to datasets used to train some disorder predictors (for example, refs. ^19,20,22^) and for CASP disorder challenges^31^.

The distribution of organisms reflects what is known from other studies^4,5^, with the majority of ID targets coming from eukaryotes, a good representation of viruses and bacteria but much fewer from archaea (Fig. 1f). At the species level, annotations are strongly biased in favor of model organisms with a majority from human, mouse, rat, *Escherichia coli* and several other common model organisms (Supplementary Fig. 6). Target proteins are not redundant at the sequence level, and are different from known examples available in the previous DisProt release. Mean sequence identity is 22.2% against the previous DisProt release and 17.1% within the dataset (Supplementary Fig. 3). CAID has two main categories—the prediction of ID and the prediction of binding sites found in IDRs. ID prediction can be further divided into prediction of IDRs and prediction of fully disordered proteins.

## IDR prediction performance

**Fig. 2:**
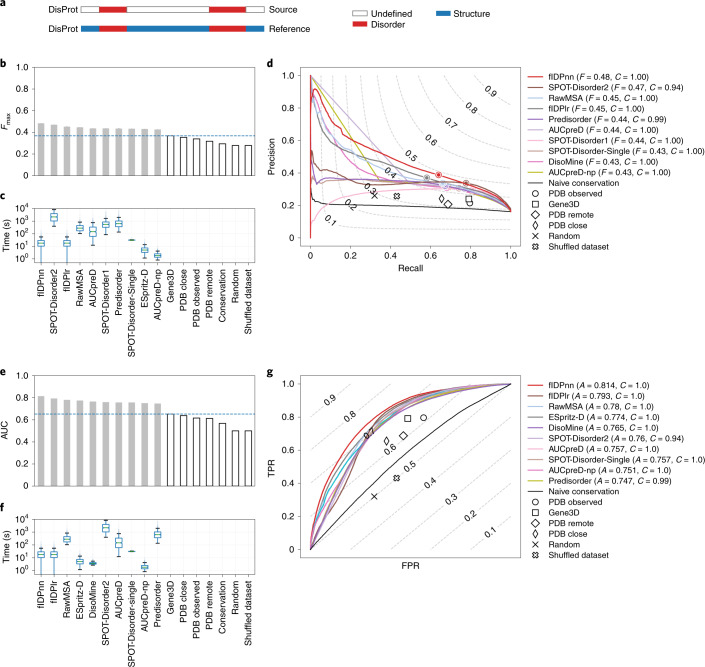
Prediction success and CPU times for the ten top-ranking disorder predictors in the DisProt dataset. **a**, The reference used (DisProt, *n* = 646 proteins) in the analysis and how it was obtained. **b**–**g**, Performance of predictors expressed as maximum *F*1-score across all thresholds (*F*_max_) (**b**) and AUC (**e**) for the ten top-ranking methods (light gray) and baselines (white), and distribution of execution time per target (**c**,**f**) using the DisProt dataset. **b**,**e**, The horizontal line indicates, respectively, *F*_max_ and AUC of the best baseline. **d**,**g**, Precision–recall (**d**) and ROC curves (**g**) of the ten top-ranking methods and baselines using the DisProt dataset, with level curves of *F*1-score and balanced accuracy, respectively. *F*, *F*_max_; *C*, coverage; *A*, AUC. **c**,**f**, Boxplots are defined as follows: the middle value of the dataset is the median (Q2/50th percentile) and box boundaries are the first quartile (Q1/25th percentile) and third quartile (Q3/75th percentile), respectively; maximum is Q3 + 1.5 × (Q3 – Q1) and minimum is Q1 – 1.5 × (Q3 – Q1). Outliers are hidden for clarity. **c**,**f**, Magenta dots indicate that the entire distribution of execution times is <1 s. Q1–Q3, first to third quartiles. TPR, true positive rate; FPR, false positive rate.

**Fig. 3:**
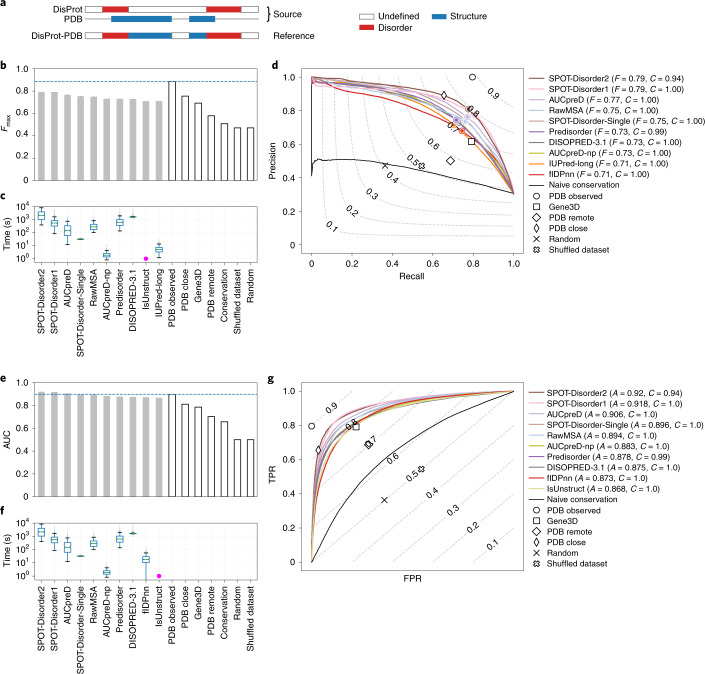
Prediction success and CPU times for the ten top-ranking disorder predictors in the DisProt-PDB dataset. **a**, The reference used (DisProt-PDB, *n* = 646 proteins) in the analysis and how it was obtained. **b**–**g**, Performance of predictors expressed as maximum *F*1-score across all thresholds (*F*_max_) (**b**) and AUC (**e**) for the ten top-ranking methods (light gray) and baselines (white), and distribution of execution time per target (**c**,**f**) using the DisProt-PDB dataset. **b**,**e**, The horizontal line indicates, respectively, *F*_max_ and AUC of the best baseline. **d**,**g**, Precision–recall (**d**) and ROC curves (**g**) of the ten top-ranking methods and baselines using the DisProt-PDB dataset, with level curves of *F*1-score and balanced accuracy, respectively. **c**,**f**, boxplots are defined as follows: the middle value of the dataset is the median (Q2/50th percentile) and box boundaries are the first quartile (Q1/25th percentile) and third quartile (Q3/75th percentile), respectively; maximum is Q3 + 1.5 × (Q3 – Q1) and minimum is Q1 – 1.5 × (Q3 – Q1). Outliers are hidden for clarity. **c**,**f**, Magenta dots indicate that the entire distribution of execution times is <1 s.

The quality of IDR prediction can be evaluated in different ways. In some cases, it is relevant to know the fraction of disorder while in others it is more important to know the exact position of the IDR in the sequence. Since disorder can be used as a proxy either to estimate the complexity of an organism or complement a sequence search, it is also important for a predictor to be sufficiently rapid for genome-scale application. For CAID, we report the maximum *F*1-score (*F*_max_—that is, maximum harmonic mean between precision and recall across all thresholds), which takes into account predictions across the entire sensitivity spectrum^32^. The performance of top methods, based on *F*_max_ and calculated over all targets, is shown in Figs. 2 and 3 for the datasets DisProt and DisProt-PDB, respectively. The *F*1-score, which is insensitive to dataset imbalance (Fig. 1d), provides a ranking almost identical to that obtained with Matthews correlation coefficient (MCC). Supplementary Figs. 12, 13, 33 and 34 show a full comparison and the dependence of *F*1-score and MCC on predictor confidence scores, along with the predictor default confidence threshold (Supplementary Figs. 10, 11, 30 and 31). All methods were compared with the various baselines described in Methods. In some applications, the objective was to predict which protein fragments are disordered based on known examples in the PDB. This is a different problem than prediction of functional IDRs—for example, aiming to evaluate their biophysical properties. The naive baselines help us understand this difference and assess the effectiveness of the transfer-by-homology of structural information for IDR prediction (Discussion). In the PDB Observed baseline, mimicking perfect knowledge, all residues not covered by any PDB structure are labeled as disordered. Alternatively, in the Gene3D baseline, residues are considered disordered if they do not match any Gene3D prediction for homologous domains. In the Shuffled dataset baseline, the reference is randomly shuffled at the dataset level while Random is an actual random predictor that does not use any previous knowledge.

The values of *F*_max_ (Fig. 2b,d) and area under the receiver operating characteristic (ROC) curve (AUC) (Fig. 3e,g) were substantially different when predictors were tested on the DisProt dataset, which contained uncertain residues, as opposed to the DisProt-PDB dataset. By definition, the PDB Observed baseline cannot predict negative residues outside PDB regions: it generates 56.5% false positives, which dropped to zero when considering the DisProt-PDB dataset in which the uncertain residues are completely filtered out. IDRs overlapping PDB regions, usually corresponding to residues involved in folding-upon-binding events, instead generate false negatives. These are far less common (20.4%) and remain the same for the two datasets. The Gene3D baseline typically increases PDB coverage (negatives), exploiting the transfer-by-homology principle. As a consequence, the probability of false positives is lower (48.6%) and false negatives are only marginally more frequent (20.9%). For the DisProt dataset, Gene3D slightly outperforms PDB Observed in terms of both *F*_max_ (Fig. 2b,d) and AUC (Fig. 3e,g). Rather, for the DisProt-PDB dataset, PDB Observed is notably superior to all methods with only 6.3% mispredicted residues, all false negatives. Given the relevance of the host organism in determining environmental factors for IDPs such as temperature, we wondered whether predictor performance would be affected in different subsets. Performance was assessed separately for mammalian and prokaryotic proteins (Supplementary Figs. 19–28 show the DisProt dataset and Supplementary Figs. 40–49 show the DisProt-PDB dataset). The ranking changes only slightly after the top two positions. Performance for mammalian sequences is ~0.05 and ~0.03 lower in terms of *F*_max_ and AUC, respectively, for all methods, suggesting that this is a somewhat harder challenge.

Across the different performance measures, the methods SPOT-Disorder2, fIDPnn, RawMSA and AUCpreD are consistently found among the top five. While the ordering changes for different measures and reference sets, and the differences among them are not statistically significant (Supplementary Figs. 17, 18, 23, 28, 38, 39, 44 and 49), these methods can be seen broadly as performing consistently well. Looking at the precision–recall curves (Fig. 2d), we notice that the top five methods (excluding fIDPnn/lr in the DisProt dataset and AUCpred-np in the DisProt-PDB dataset) leverage evolutionary information, introducing a database search as a preliminary step. The performance gain, on average 4.5% in terms of *F*_max_, comes at the cost of slowing prediction by two to four orders of magnitude (Fig. 2c and Supplementary Figs. 4, 12–14 and 33–35).

## Fully disordered proteins

We considered fully disordered proteins (IDPs) separately because these are particularly challenging to investigate experimentally; for example, they cannot be probed with X-ray crystallography yet they are of great interest because they fulfill unique biological functions^5,33^. We therefore designed another classification challenge: separation of IDPs from all other proteins. We consider proteins as IDPs when at least 95% of residues are predicted or annotated as disordered, and predictors were asked to identify IDPs based on this criterion. According to this definition, the number IDPs in the DisProt dataset is 40 out of 646. Different threshold values did not substantially affect the ranking (Supplementary Tables 6–8). In Table 1 all methods are sorted based on *F*1-score. False positives are limited for many methods, although correct IDP predictions are generally made for less than half of the dataset. The fraction of residues predicted as disordered is also notably different across methods (Supplementary Fig. 50), suggesting room for improvement. Methods using secondary structure information may be at a disadvantage for IDP prediction, since annotations frequently rely on detection methods without residue-level resolution (for example, circular dichroism; Supplementary Fig. 7).

**Table 1:** Confusion matrix and metrics for the prediction of fully disordered proteins in the DisProt dataset

	TN	FP	FN	TP	MCC	*F*1-s	TNR	TPR	PPV	BAC
fIDPnn	585	16	19	26	0.569	0.598	0.973	0.578	0.619	0.776
RawMSA	582	19	19	26	0.546	0.578	0.968	0.578	0.578	0.773
VSL2B	578	23	22	23	0.468	0.505	0.962	0.511	0.500	0.736
fIDPlr	566	35	18	27	0.468	0.505	0.942	0.600	0.435	0.771
Predisorder	589	12	26	19	0.479	0.500	0.980	0.422	0.613	0.701
SPOT-Disorder1	572	29	23	22	0.416	0.458	0.952	0.489	0.431	0.720
DisoMine	551	50	17	28	0.421	0.455	0.917	0.622	0.359	0.770
AUCpreD	588	13	28	17	0.431	0.453	0.978	0.378	0.567	0.678
SPOT-Disorder2	574	27	24	21	0.409	0.452	0.955	0.467	0.438	0.711
SPOT-Disorder-Single	594	7	30	15	0.452	0.448	0.988	0.333	0.682	0.661
IsUnstruct	588	13	29	16	0.411	0.432	0.978	0.356	0.552	0.667
IUPred2A-long	595	6	32	13	0.420	0.406	0.990	0.289	0.684	0.639
**Gene3D**	505	96	10	35	0.391	0.398	0.840	0.778	0.267	0.809
ESpritz-N	597	4	33	12	0.426	0.393	0.993	0.267	0.750	0.630
ESpritz-D	555	46	23	22	0.342	0.389	0.923	0.489	0.324	0.706
PyHCA	596	5	33	12	0.411	0.387	0.992	0.267	0.706	0.629
JRONN	595	6	33	12	0.397	0.381	0.990	0.267	0.667	0.628
MobiDB-lite	599	2	34	11	0.437	0.379	0.997	0.244	0.846	0.621
DisPredict-2	586	15	32	13	0.330	0.356	0.975	0.289	0.464	0.632
IUPred2A-short	599	2	35	10	0.413	0.351	0.997	0.222	0.833	0.609
S2D-2	572	29	30	15	0.288	0.337	0.952	0.333	0.341	0.643
**PDB Observed**	468	133	13	32	0.286	0.305	0.779	0.711	0.194	0.745
AUCpreD-np	590	11	35	10	0.293	0.303	0.982	0.222	0.476	0.602
ESpritz-X	595	6	36	9	0.321	0.300	0.990	0.200	0.600	0.595
FoldUnfold	456	145	14	31	0.256	0.281	0.759	0.689	0.176	0.724
DISOPRED-3.1	596	5	39	6	0.246	0.214	0.992	0.133	0.545	0.563
DisEMBL-HL	601	0	41	4	0.288	0.163	1.000	0.089	1.000	0.544
**PDB Remote**	590	11	42	3	0.085	0.102	0.982	0.067	0.214	0.524
DisEMBL-465	601	0	43	2	0.204	0.085	1.000	0.044	1.000	0.522
**PDB Close**	589	12	43	2	0.043	0.068	0.980	0.044	0.143	0.512
**Conservation**	441	160	38	7	−0.064	0.066	0.734	0.156	0.042	0.445
DynaMine	601	0	45	0	0.000	0.000	1.000	0.000	0.000	0.500
GlobPlot	601	0	45	0	0.000	0.000	1.000	0.000	0.000	0.500
DFLpred	601	0	45	0	0.000	0.000	1.000	0.000	0.000	0.500

TN, true negatives count; TP, true positives count; FN, false negatives count; FP, false positives count; *F*1-s, *F*1-score; TNR, true negative rate, specificity; TPR, true positive rate, recall; PPV, positive predictive value, precision; BAC, balanced accuracy for prediction of fully disordered proteins. Proteins with disorder prediction or disorder annotation covering at least 95% of the sequence are considered fully disordered. Predictors are sorted by their *F*1-score. Baseline names are in bold.

## Prediction of disordered binding sites

**Fig. 4:**
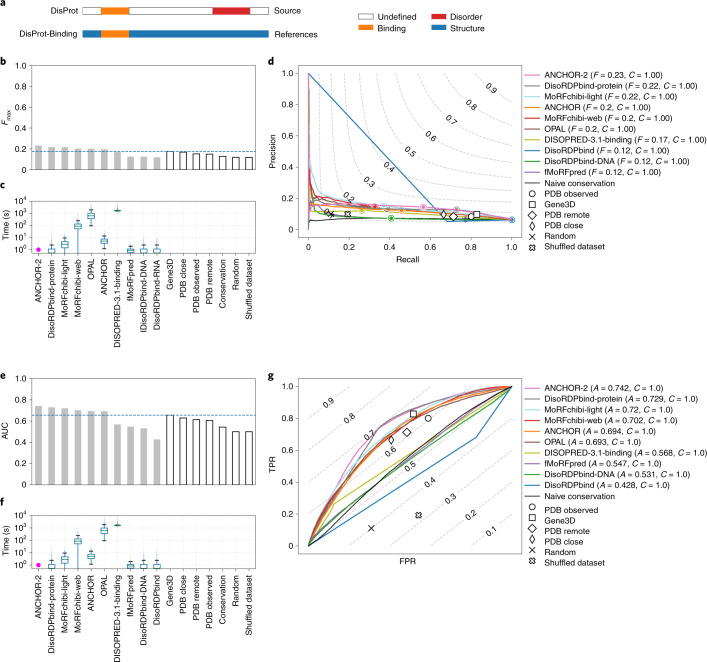
Prediction success and CPU times for the ten top-ranking binding predictors in the DisProt-binding dataset. **a**, The reference used (DisProt-binding, *n* = 646 proteins) in the analysis and how it was obtained. **b**–**g**, Performance of predictors expressed as maximum *F*1-score across all thresholds (*F*_max_) (**b**) and AUC (**e**) for the ten top-ranking methods (light gray) and baselines (white), and distribution of execution time per target (**c**,**f**) using the DisProt-binding dataset. **b**,**e**, The horizontal line indicates, respectively, *F*_max_ and AUC of the best baseline. **d**,**g**, Precision–recall (**d**) and ROC curves (**g**) of the ten top-ranking methods and baselines using the DisProt-binding dataset, with level curves of *F*1-score and balanced accuracy, respectively. **c**,**f**, boxplots are defined as follows: the middle value of the dataset is the median (Q2/50th percentile) and box boundaries are the first quartile (Q1/25th percentile) and third quartile (Q3/75th percentile), respectively; maximum is Q3 + 1.5 × (Q3 – Q1) and minimum is Q1 – 1.5 × (Q3 – Q1). Outliers are hidden for clarity. **c**,**f**, Magenta dots indicate that the entire distribution of execution times is <1 s.

As a second major challenge, CAID evaluated the prediction of binding sites within IDRs, commonly referred to as linear interacting peptides^24^ or short linear motifs^34^ leveraging DisProt annotations for binding regions (Supplementary Fig. 52 shows dataset composition and overlap to other databases). In DisProt, binding annotations retrieved from the literature are fraught with more ambiguity than disorder examples. In addition, experimental evidence for the exact position of a binding region is often inaccurate because binding is annotated as a feature of an IDR. Our reference includes all entries in the DisProt dataset, even if they were not annotated with binding regions. This translates to a dataset where the majority of targets (414 out of 646) have no positives. In this challenge, we retained the PDB Observed and Gene3D baselines even if they were not designed to detect binding regions. Because target binding regions in DisProt are found within IDRs, the baselines are expected to attain high recall and low precision. All models perform poorly, as do the naive baselines (Fig. 4b,d). At *F*_max_, their recall is higher than their precision as for the baselines (Fig. 4c). However, the top five methods—ANCHOR-2 (ref. ^21^), DisoRDPbind^35^, MoRFchibi (light and web)^36^ and OPAL^37^—perform better than the baselines (Fig. 4b), which trade off considerably more precision due to an abundant overprediction. The execution times of the top five methods have very different scales and are inversely proportional to their performance, with the best methods requiring less central processing unit (CPU) time. The performance of predictors on mammalian and prokaryotic proteins for the DisProt-binding dataset is only marginal (Supplementary Figs. 63–72).

## Software implementation

We also evaluated those technical aspects related to software implementation—that is, speed and usability—that have a direct impact on their application for large-scale analyses. Speed in particular is highly variable, with methods of comparable performance varying by up to four orders of magnitude in execution time (Supplementary Fig. 4). In general, all methods incorporate a mix of different scripts and programming languages. Some software configuration scripts contain errors. In many cases data paths and file names are hardcoded in the program—for example, the sequence database or output file path. Only a few programs allow specification of a temporary folder, which is important for parallel execution. It is possible to provide precalculated sequence searches for only a few methods. Several methods implemented are reliant on dependencies, sometimes on specific software versions or CPUs with a modern instruction set. Some programs are particularly eager for random-access memory (RAM), crashing with longer input sequences or do not have a timeout control and execute forever. Output formats differ, with some not adequately documented. Only a few software programs support multithreading and only one was submitted as a Docker container. In summary, the software implementation for disorder predictors has considerable room for improvement regarding practical purposes.

## Discussion

The problem of predicting protein ID is challenging, for several reasons. The first is in the definition of ID, indicating that a protein sequence does not encode a stable structural state that is ordered. Defining ID as a property that a protein does not have (that is, order) implies that many conformational states fit the definition, covering a continuum between fully disordered states and folded states with long dynamic regions^38,39^. The second problem is the lack of a consensus reference experimental method, or set of experimental methods, yielding an operational definition of ID (compared to X-ray crystallography in the definition of ordered structures). The third problem is the dependence of ID on events or conditions at certain points in time along the life of a protein. Some proteins remain unfolded until they bind a partner^40^ while others are disordered providing they are in a specific cellular compartment and fold following translocation^41^, and some enzymes undergo order-to-disorder-to-order transition as part of their catalytic cycle^42^. Given these challenges, CAID represents a community-based effort to develop and implement evaluation strategies to assess (1) clear definitions of ID and (2) the performance of methods used in the prediction of ID. In its first round, CAID leverages the DisProt database^23^ of curated experimental evidence to assess ID predictors. In DisProt, curators store the coordinates of IDRs when there is experimental evidence in peer-reviewed articles of highly mobile residue stretches longer than ten residues. We anticipate that future rounds may include reference data arising from ever-improving consensus operational definitions—for example, NMR measurements, which are particularly powerful in the characterization of experimental protein disorder. For example, one could define disordered regions as those that exhibit high conformational variability under physiological conditions using multiple orthogonal measures. ID predictors were previously assessed from the fifth to the tenth editions of CASP, but this was abandoned due to the lack of good reference data.

A long-term goal for CAID is to help the selection of candidate IDPs for experimental testing. One of the main properties of IDPs is their ability to form many low-affinity and high-specificity interactions^43^. It remains challenging to predict the interacting residues of an IDP from its sequence. At present, multiple high-throughput experiments are available for the detection of interactions capable of resolving interacting regions^44^. However, binding sites obtained from high-throughput experiments (for example, CoIP, Y2H) and reported in the literature often lack this grade of resolution. Furthermore, while some attempts have been made to mitigate this problem^45^, a high false-positive rate plagues all experimental methods used to identify binding: proteins interacting in experimental conditions do not necessarily interact in the cell under physiological physicochemical conditions, or simply due to spatiotemporal segregation^46^. DisProt annotates binding partners and interaction regions of IDPs used in CAID to attempt the first assessment of binding predictors.

One of the major challenges in CAID is the definition of negatives—that is, residues that are not disordered or do not bind. Knowledge about negative results is a long-standing problem in biology^47^ and is especially relevant for our assessment. If the annotation of IDRs in a protein is not complete, how do we know which regions are structured? This is even more relevant for binding regions, because we are far from being able to map all binding partners of a protein with residue resolution under different cellular settings. To overcome this problem, which is intrinsic to how we detect and store data, ID predictor performance was tested in two scenarios. In the first of these we assumed that all annotations were complete, considering all residues outside of annotated regions as structured. In the second scenario, we used resolved residues from PDBs to annotate structure and filtered out all residues that were covered by neither disorder nor structure annotation. Binding site predictors were tested on a dataset where all residues outside of binding regions are considered nonbinding.

Despite these challenges, CAID revealed progress in the detection of ID from sequence and highlighted that there remains scope for improvement in both disorder and binding site predictors. One of the primary goals was to determine whether automated algorithms perform better than naive assumptions such as sequence conservation or three-dimensional structure. As far as ID is concerned, the performance of predictors in comparison to naive baselines largely depends on the assumption made on nondisordered residues. On the DisProt-PDB dataset, where disorder is inferred from DisProt annotation, order from the presence of a PDB structure and all other residues is filtered out: naive baselines outperform predictors. However, when only DisProt annotations are considered (DisProt dataset), the tables are turned and predictors, while obtaining lower overall scores, outperform naive baselines (Figs. 2 and 3). When uncertain residues are retained in the analysis (DisProt dataset), the number of false positives increases and precision plunges, lowering the *F*1-score. This means that either predictors detect ID in the uncertain residues—suggesting that DisProt annotation is incomplete, predictors overpredict or both. Naive baselines are outperformed by predictors since they predict all uncertain residues as disordered, which are all counted as false positives. This suggests that predictors have reached a state of maturity and can be trusted with relative confidence when no experimental evidence is available. It also confirms that when experimental evidence is present, it is more reliable than predictions.

An interesting special case is how predictors behave with fully disordered DisProt targets (Table 1). This case is compelling because predictors are usually not trained on these examples. Predictors vastly outperform naive baselines in these cases due to their large overprediction. The count of false positives puts baselines at a disadvantage, compensating for their low count of false negatives. PDB Observed classifies a protein as fully disordered whenever no structure is available for that protein. However, the absence of a protein from PDB may be simply due to the lack of studies on that protein. Gene3D performs better since it generalizes from existing structures, but still tends to overpredict disorder (or underpredict order). At the opposite side of the spectrum, methods that are too conservative in their disorder classification (for example, MobiDB-lite) perform worse than expected on fully disordered proteins. Results from the DisProt dataset suggest that several methods are consistently among the top performers, although the exact ranking is subject to some variation. fIDPnn and SPOT-Disorder2 perform consistently well, with RawMSA and AUCpreD following closely. The execution times for these four methods vary by up to three orders of magnitude, suggesting there is room for optimization of the software. Of note, both fIDPnn and RawMSA were unpublished at the time of the CAID experiment. While top-performing methods are able to achieve a certain balance between under- and overprediction, it is interesting to note how they are not able to identify all fully disordered targets. Not even methods that trade off specificity to increase the detection of relevant cases are able to attain full sensitivity. This confirms that predictors are not trained on this particular class of proteins, and suggests that they have room for improvement in this direction.

CAID offers an attempt at assessment of binding predictors. As discussed above, this is intrinsically difficult due to the complex nature of this phenomenon and how it is detected and stored. While we are aware of these difficulties, we still think that an assessment is useful for researchers who either use or develop binding predictors. Furthermore, while it is arguable that this evaluation has limitations, its publication helps highlight such constraints and exposes this problem to the rest of the scientific community. We compared predictors to the same baselines used for the disorder challenge but, while their design remains unchanged, their underlying naive assumption changes slightly. The PDB Observed baseline assumes that whatever is not covered by a structural annotation in PDB is not only disordered but also involved in one or more interactions. When considering all targets in the CAID dataset, including those not annotated as binders, predictors slightly outperform the baselines but have limited performance overall. Figure 4 shows disagreement with the DisProt-binding reference in both positive and negative classification, highlighting the potential for improvement of binding predictors. We have to consider that the dataset used is strongly unbalanced. Although a prominent function of IDPs is mediation of protein–protein interactions, most targets (414 of 646) do not contain an identified binding region and those that do include binding regions often have them spanning the whole disordered region in which they are found. This strong bias is due to how DisProt was previously annotated, with the label ‘binding’ being associated with an entire IDR. In the latest DisProt version this annotation style has been replaced with a more detailed one, ensuring that future editions of CAID will be less biased towards long binding regions. The improved definition of boundaries for disordered binding regions could favor methods trained specifically to recognize shorter binding regions. Overall, this suggests a large growth potential in both predictors and reference sets for this challenge.

In conclusion, the CAID experiment has provided a fully blind assessment of ID predictors, almost a decade after CASP stopped assessing them, and a new assessment of ID binding regions. The results are encouraging, showing that the methods are sufficiently mature to be useful but also that substantial room for improvement remains. As the quality of ID data improves, we expect predictors to become more accurate and reliable.

## Methods

All software programs were executed using a homogeneous cluster of nodes running Ubuntu 16.04 on Intel 8 core processors with 16 GB of RAM and a mechanical hard disk. In the text we refer to proteins as targets, to disordered residues as positive labels and to structured/ordered residues as negative labels. Experiment design is described in the Nature Research Reporting Summary.

### Reference sets

In CAID different reference sets were built, differing in the subset of DisProt used to define positive labels and in the definition of negatives labels.

For the disorder challenge, we generated two reference sets called DisProt and DisProt-PDB. Both references are composed of a set of 646 targets, annotated between June 2018 and November 2018 (DisProt release 2018_11). Positive labels in both reference sets are those residues annotated as disordered in the DisProt database. In the DisProt reference set, all labels not positive are assigned as negatives. In the DisProt-PDB set, PDB structures mapping on the protein sequence define negative labels. All residues not covered by either DisProt annotation or PDB structures are masked and were excluded from the analysis. It should be noted that a fraction of resolved structures in the PDB has been annotated as disordered^48,49^. While in this edition of CAID we decided to consider any resolved residue from crystallography, NMR or electron microscopy experiments (excluding those overlapping with DisProt annotation) as structured, we plan to apply a filtering on subsequent editions. This problem will become progressively less relevant as DisProt annotations become more complete, since disorder always overwrites structure.

For the binding challenge we generated a reference set that we called DisProt-binding. Positive labels are those residues annotated as binding in the DisProt database, whereas all labels not positive are assigned as negatives. Notice that 232 targets have at least one annotation of binding in the DisProt database. Because DisProt-binding is composed of all 646 targets considered in the analysis, the majority of targets (that is, 646 – 232 = 414) do not contain positive labels.

### Predictions

Most predictors output a series of score and state pairs per residue of the input sequence. Scores are floating point numbers while states are binary labels predicting whether a residue is in a disordered or structured state. If scores are missing, states will be used as scores. If states are missing, they are generated by applying a threshold to scores. By default, thresholds are inferred from states. When states are not available and a threshold is not specified by the authors of the method, we set the threshold to 0.5. This ensures correct default threshold estimates for any distribution of scores. Prediction scores are rounded to the third decimal figure, which sets the number of possible thresholds to 1,000. Bootstrapping samples the whole dataset with replacements 1,000 times. Resampling is done at the label (residue) level. Confidence intervals are calculated on Student’s *t*-distribution at alpha set to 0.05.

### Baselines

A number of baseline predictors have been built for comparison with actual predictors. Two are based on randomization of the dataset (Shuffled dataset, Random) and one on an estimate of residue conservation through evolution (Conservation). The last four consider the opposite of structure as disorder (PDB Observed, PDB Close, PDB Remote and Gene3D).

The Shuffled dataset is a reshuffling of the DisProt dataset—that is, random permutation of labels across the entire dataset. This preserves the proportion of positive labels across the dataset but not necessarily for each single target. The Random baseline is a random classifier in which the prediction score of each label is assigned randomly. It is built by randomly drawing floating point numbers out of a uniform distribution [0,..,1] and applying a threshold of 0.5.

The Conservation baseline uses the naive consideration that IDPs on average are less conserved than globular proteins. It is calculated from the distance between the residue frequencies of homologous sequences for each target against the residue frequencies of the BLOSUM62 alignments. Amino acid frequencies for the targets are extracted from the position-specific scoring matrix generated by running three iterations of PSI-BLAST^50^ against UniRef90. The distance is calculated from the Jensen–Shannon divergence^51^ of the two frequencies. This returns values in the [0,…,1] interval where any position with a score >0.4 is considered positive (that is, disordered).

Several naive baselines are based on the assumption that whatever is not annotated as structure in the PDB is disordered. PDB Observed has the structure annotation defined by PDB structures as mapped on UniProt sequences by Mobi 2.0 (ref. ^52^) (October 2019). Whenever we are unable to map perfectly the PDB sequence on the UniProt sequence, unmapped residues were considered not observed and excluded from the analysis. This applies to His-tags, mutated sequences and missing residues (in both X-ray and NMR structures); PDB Close and PDB Remote have the structure annotation defined by observed residues in PDBs with similar sequence. The similarity is calculated as the identity percentage given by a three-iteration PSI-BLAST^50^ of DisProt targets against PDB seqres. PDB Close considers PDB structures with at least 30% sequence identity (that is, close homologs), while PDB remote considers only PDB structures with sequence identity 20–30% (that is, remote homologs). Gene3D has structure annotations defined by Gene3D^53^ (v.4.2.0) predictions, calculated with InterProScan^28^ (v.5.38–76.0).

### Target and dataset metrics

Metrics were calculated following two strategies—dataset and target. In the dataset strategy, all targets (proteins) reference classifications and prediction classifications are concatenated in two single arrays. Confusion matrix and subsequent evaluation metrics are calculated once, comparing these arrays. In the target strategy confusion matrix and subsequent evaluation, metrics are calculated for each target (protein) and the mean value of the evaluation metrics is taken. The former strategy is equivalent to summing the confusion matrices for each target and computing evaluation metrics on the resulting confusion matrix, while the latter is equivalent to calculation of the evaluation metrics on the average of the confusion matrices of the targets.

### Notes on calculation of evaluation metrics

Throughout the manuscript, *F*_max_ and AUC are the main assessment criteria used. *F*_max_ is the maximum point in the precision–recall curve while AUC is the area under the ROC curve. Additional metrics are used for comparison, and they all follow standard definitions as described in Supplementary Table 4. *F*-beta (0.5, 1, 2) and MCC are set to 0 if the denominator is 0. Since the MCC denominator is a multiplication of the number of positive and negative classifications and positive and negative labels in the reference, if any of these classes amounts to 0 we set MCC to 0. This means that, for both fully disordered proteins and those predicted to be fully disordered or fully ordered, MCC is 0. This situation is very likely in target strategy with the DisProt-PDB dataset, and explains why the MCC for target strategy is much lower than that for the dataset strategy (Supplementary Fig. 34). This effect can also be seen in the heatmap of target MCC, where a large number of targets have MCC = 0.

### Statistics

In ranking plots (Supplementary Figs. 17, 18, 23, 28, 38, 39, 44, 49, 61, 66 and 71), *P* values are calculated with a two-tailed *t*-test. The bootstrapping used in Figs. 2–4 samples the whole dataset with replacements 1,000 times. Resampling is done at the label (residue) level. Confidence intervals are calculated based on Student’s *t*-distribution at alpha set to 0.05.

### Assessors’ policy

Prediction methods published by the assessors were not included in the challenges: their methods are included for reference only.

### Reporting Summary

Further information on research design is available in the Nature Research Reporting Summary linked to this article.

## Online content

Any methods, additional references, Nature Research reporting summaries, source data, extended data, supplementary information, acknowledgements, peer review information; details of author contributions and competing interests; and statements of data and code availability are available at 10.1038/s41592-021-01117-3.

## Supplementary information

Supplementary Information Supplementary Tables 1–12 and Figs. 1–72.

Reporting Summary

## Data Availability

Raw DisProt annotations, reference datasets and predictions in CAID format are available at https://idpcentral.org/caid/data/1/. Description of the process and code to produce references is available in the GitHub CAID repository at https://github.com/BioComputingUP/CAID. All data used in the analysis are also available in the Code Ocean capsule (10.24433/CO.3610625.v1).

## Code availability

Results of the CAID assessment can be fully reproduced by downloading the code and following the instructions in the CAID GitHub repository at https://github.com/BioComputingUP/CAID. The CAID software is a Python 3 package that produces all outputs necessary for CAID, including baselines, references and plots. See Data availability for information about how to obtain Input predictions, references and sequence annotations. The CAID package is dependent on public Python 3 libraries and on the vectorized_cls_metrics library, available at https://github.com/marnec/vectorized_cls_metrics. The code is also available and reproducible in the Code Ocean capsule (10.24433/CO.3610625.v1).

## References

[1] Tompa, P. & Fersht, A. *Structure and Function of Intrinsically Disordered Proteins* (CRC Press, 2009).

[2] Dunker AK, Bondos SE, Huang F, Oldfield CJ (2015). Intrinsically disordered proteins and multicellular organisms. Semin. Cell Dev. Biol..

[3] Wright PE, Dyson HJ (2015). Intrinsically disordered proteins in cellular signalling and regulation. Nat. Rev. Mol. Cell Biol..

[4] Ward JJ, Sodhi JS, McGuffin LJ, Buxton BF, Jones DT (2004). Prediction and functional analysis of native disorder in proteins from the three kingdoms of life. J. Mol. Biol..

[5] Necci M, Piovesan D, Tosatto SCE (2016). Large-scale analysis of intrinsic disorder flavors and associated functions in the protein sequence universe. Protein Sci..

[6] Melo AM (2016). A functional role for intrinsic disorder in the tau–tubulin complex. Proc. Natl Acad. Sci. USA.

[7] Dev KK, Hofele K, Barbieri S, Buchman VL, van der Putten H (2003). Part II: alpha-synuclein and its molecular pathophysiological role in neurodegenerative disease. Neuropharmacology.

[8] Iakoucheva LM, Brown CJ, Lawson JD, Obradović Z, Dunker AK (2002). Intrinsic disorder in cell-signaling and cancer-associated proteins. J. Mol. Biol..

[9] Cheng Y (2006). Rational drug design via intrinsically disordered protein. Trends Biotechnol..

[10] Uversky VN (2012). Intrinsically disordered proteins and novel strategies for drug discovery. Expert Opin. Drug Discov..

[11] Mohan A, Uversky VN, Radivojac P (2009). Influence of sequence changes and environment on intrinsically disordered proteins. PLoS Comput. Biol..

[12] Plitzko JM, Schuler B, Selenko P (2017). Structural biology outside the box—inside the cell. Curr. Opin. Struct. Biol..

[13] Tompa P (2011). Unstructural biology coming of age. Curr. Opin. Struct. Biol..

[14] Holmstrom ED, Nettels D, Schuler B (2018). Conformational plasticity of hepatitis C virus core protein enables RNA-induced formation of nucleocapsid-like particles. J. Mol. Biol..

[15] Felli, I. C. & Pierattelli, R. *Intrinsically Disordered Proteins Studied by NMR Spectroscopy* (Springer, 2015).

[16] Williams RJ (1978). The conformational mobility of proteins and its functional significance. Biochem. Soc. Trans..

[17] Liu Y, Wang X, Liu B (2019). A comprehensive review and comparison of existing computational methods for intrinsically disordered protein and region prediction. Brief. Bioinform..

[18] Peng K, Radivojac P, Vucetic S, Dunker AK, Obradovic Z (2006). Length-dependent prediction of protein intrinsic disorder. BMC Bioinformatics.

[19] Linding R (2003). Protein disorder prediction: implications for structural proteomics. Structure.

[20] Jones DT, Cozzetto D (2015). DISOPRED3: precise disordered region predictions with annotated protein-binding activity. Bioinformatics.

[21] Mészáros, B., Erdős, G. & Dosztányi, Z. IUPred2A: context-dependent prediction of protein disorder as a function of redox state and protein binding. *Nucleic Acids Res*. **46**, W329–W337 (2018). 10.1093/nar/gky384 PMC6030935 29860432

[22] Walsh I, Martin AJM, Di Domenico T, Tosatto SCE (2012). ESpritz: accurate and fast prediction of protein disorder. Bioinformatics.

[23] Hatos, A. et al. DisProt: intrinsic protein disorder annotation in 2020. *Nucleic Acids Res*. **48**, D269–D276 (2020). 10.1093/nar/gkz975 PMC7145575 31713636

[24] Piovesan D (2018). MobiDB 3.0: more annotations for intrinsic disorder, conformational diversity and interactions in proteins. Nucleic Acids Res..

[25] Fukuchi S (2014). IDEAL in 2014 illustrates interaction networks composed of intrinsically disordered proteins and their binding partners. Nucleic Acids Res..

[26] Schad E (2018). DIBS: a repository of disordered binding sites mediating interactions with ordered proteins. Bioinformatics.

[27] Fichó E, Reményi I, Simon I, Mészáros B (2017). MFIB: a repository of protein complexes with mutual folding induced by binding. Bioinformatics.

[28] Mitchell AL (2019). InterPro in 2019: improving coverage, classification and access to protein sequence annotations. Nucleic Acids Res..

[29] The UniProt Consortium. (2017). UniProt: the universal protein knowledgebase. Nucleic Acids Res..

[30] Velankar S (2016). PDBe: improved accessibility of macromolecular structure data from PDB and EMDB. Nucleic Acids Res..

[31] Monastyrskyy B, Kryshtafovych A, Moult J, Tramontano A, Fidelis K (2014). Assessment of protein disorder region predictions in CASP10. Proteins.

[32] Radivojac P (2013). A large-scale evaluation of computational protein function prediction. Nat. Methods.

[33] Deiana A, Forcelloni S, Porrello A, Giansanti A (2019). Intrinsically disordered proteins and structured proteins with intrinsically disordered regions have different functional roles in the cell. PloS ONE.

[34] Kumar, M. et al. ELM—the eukaryotic linear motif resource in 2020. *Nucleic Acids Res*. **48**, D296–D306 (2020). 10.1093/nar/gkz1030 PMC7145657 31680160

[35] Peng Z, Kurgan L (2015). High-throughput prediction of RNA, DNA and protein binding regions mediated by intrinsic disorder. Nucleic Acids Res..

[36] Malhis N, Jacobson M, Gsponer J (2016). MoRFchibi SYSTEM: software tools for the identification of MoRFs in protein sequences. Nucleic Acids Res..

[37] Sharma, R., Raicar, G., Tsunoda, T., Patil, A. & Sharma, A. OPAL: prediction of MoRF regions in intrinsically disordered protein sequences. *Bioinformatics* **34**, 1850–1858 (2018). 10.1093/bioinformatics/bty032 29360926

[38] Forman-Kay JD, Mittag T (2013). From sequence and forces to structure, function, and evolution of intrinsically disordered proteins. Structure.

[39] Sormanni P (2017). Simultaneous quantification of protein order and disorder. Nat. Chem. Biol..

[40] Dyson HJ, Wright PE (2002). Coupling of folding and binding for unstructured proteins. Curr. Opin. Struct. Biol..

[41] Jakob, U., Kriwacki, R. & Uversky, V. N. Conditionally and transiently disordered proteins: awakening cryptic disorder to regulate protein function. *Chem. Rev*. **114**, 6779–6805 (2014). 10.1021/cr400459c PMC4090257 24502763

[42] Bahar I, Chennubhotla C, Tobi D (2007). Intrinsic dynamics of enzymes in the unbound state and relation to allosteric regulation. Curr. Opin. Struct. Biol..

[43] Van Roey K (2014). Short linear motifs: ubiquitous and functionally diverse protein interaction modules directing cell regulation. Chem. Rev..

[44] Blikstad C, Ivarsson Y (2015). High-throughput methods for identification of protein–protein interactions involving short linear motifs. Cell Commun. Signal..

[45] Vidalain P-O, Boxem M, Ge H, Li S, Vidal M (2004). Increasing specificity in high-throughput yeast two-hybrid experiments. Methods.

[46] Scott JD, Pawson T (2009). Cell signaling in space and time: where proteins come together and when they’re apart. Science.

[47] Mehta, D. Highlight negative results to improve science. *Nature*10.1038/d41586-019-02960-3 (2019). 10.1038/d41586-019-02960-3 33009522

[48] Zhou J, Oldfield CJ, Yan W, Shen B, Dunker AK (2020). Identification of intrinsic disorder in complexes from the Protein Data Bank. ACS Omega.

[49] Monzon AM (2020). Experimentally determined long intrinsically disordered protein regions are now abundant in the Protein Data Bank. Int. J. Mol. Sci..

[50] Altschul SF (1997). Gapped BLAST and PSI-BLAST: a new generation of protein database search programs. Nucleic Acids Res..

[51] Capra JA, Singh M (2007). Predicting functionally important residues from sequence conservation. Bioinformatics.

[52] Piovesan, D. & Tosatto, S. C. E. Mobi 2.0: an improved method to define intrinsic disorder, mobility and linear binding regions in protein structures. *Bioinformatics* **34**, 122–123 (2018). 10.1093/bioinformatics/btx592 28968795

[53] Lewis TE (2018). Gene3D: extensive prediction of globular domains in proteins. Nucleic Acids Res..

