# Supplementary material for: Critical assessment of protein intrinsic disorder prediction
Source: Nat Methods. 2021 Apr 19;18(5):472–81. doi: 10.1038/s41592-021-01117-3 (PMC8105172; doi:10.1038/s41592-021-01117-3)
Supplement: Supplementary file 1 — Supplementary Tables 1–12 and Figs. 1–72. [file 41592_2021_1117_MOESM1_ESM.pdf]

nature portfolio

https://doi.org/10.1038/s41592-021-01117-3

Supplementary information

Critical assessment of protein intrinsic disorder prediction

In the format provided by the authors and unedited

Critical Assessment of Protein Intrinsic Disorder Prediction

Marco Necci[1,], Damiano Piovesan[1,*], CAID Predictors[#], DisProt Curators[#], Silvio C.E. Tosatto[1,+]*
[1] Dept. of Biomedical Sciences, University of Padua, Italy.
[*]*Contributed equally.*
[#]*Lists of authors and their affiliations appear at the end of the paper.*
[+]*Corresponding author silvio.tosatto@unipd.it*

Supplementary Information

Table of Contents

Tables **5**
Prediction methods 5
Supplementary Table 1. Disorder predictors 6
Supplementary Table 2. Binding predictors 7
Supplementary Table 3. Predictors description 12
DisProt dataset 12
Evaluation Results 12
Supplementary Table 4. Per-protein classification performance in the DisProt dataset 13
Supplementary Table 5. Per-residue classification performance in the DisProt dataset 14
Evaluation Results (Full ID) 14
Supplementary Table 7. Detection of proteins with more than 90% of disorder 16
Supplementary Table 8. Detection of proteins with more than 99% of disorder 17
DisProt-PDB dataset 17
Evaluation Results 17
Supplementary Table 9. Per protein classification performance in the DisProt-PDB dataset 19
Supplementary Table 10. Per-residue classification performance in the DisProt-PDB dataset 20
Disprot-Binding dataset 20
Evaluation Results 20
Supplementary Table 11. Per-protein classification performance in the DisProt-Binding dataset 20
Supplementary Table 12. Per-residue classification performance in the DisProt-Binding dataset 21

Figures **21**
Dataset 21
Annotations 21
Supplementary Figure 1. Dataset composition. 21
Supplementary Figure 2. Overlap of DisProt structural transitions with PDB. 22
Redundancy 22
Supplementary Figure 3. Dataset redundancy. 22

Predictor CPU time 22
Supplementary Figure 4. CPU time to performance for disorder predictors. 23
Supplementary Figure 5. CPU time to performance for binding predictors. 23
Species representation 24
Supplementary Figure 6. Species representation. 24
Fully ID 25
Supplementary Figure 7. Detection method of fully disordered proteins. 25
Disorder 25
DisProt dataset 25
Supplementary Figure 8: Precision recall curve in the DisProt dataset. 26
Supplementary Figure 9. F1-Score progress with threshold in the DisProt dataset. 26
Supplementary Figure 10. MCC progress with threshold in the DisProt dataset. 27
Supplementary Figure 11. Balanced accuracy progress with threshold in the DisProt dataset. 28
Supplementary Figure 12: FMax in the DisProt dataset. 29
Supplementary Figure 13: MCC in the DisProt dataset. 29
Supplementary Figure 14: Balanced accuracy in the DisProt dataset. 30
Supplementary Figure 15: MCC per protein in the DisProt dataset. 31
Supplementary Figure 16: FMax per protein in the DisProt dataset. 31
Supplementary Figure 17. Overall average ranking of all predictors and baselines in the DisProt dataset. 32
Supplementary Figure 18. Overall average ranking of the 10 best ranking predictors and baselines in the DisProt dataset. 33
Mammals 34
Supplementary Figure 19. Prediction success and CPU times for the ten top-ranking disorder predictors for mammalian proteins in the DisProt dataset. 34
Supplementary Figure 20: FMax for mammalian proteins in DisProt dataset. 35
Supplementary Figure 21. F1-Score progress with threshold for mammalian proteins in the DisProt dataset. 36
Supplementary Figure 22: FMax per target for mammalian proteins in the DisProt dataset. 37
Supplementary Figure 23. Overall average ranking of all predictors and baselines for mammalian proteins in the DisProt dataset. 38
Prokaryotes 39
Supplementary Figure 24. Prediction success and CPU times for the ten top-ranking disorder predictors for prokaryotic proteins in the DisProt dataset. 39
Supplementary Figure 25: FMax for prokaryotic proteins in the DisProt dataset. 40
Supplementary Figure 26. F1-score progress with threshold for prokaryotic proteins in the DisProt dataset. 41
Supplementary Figure 27: FMax per protein for prokaryotic proteins in the DisProt dataset. 42
Supplementary Figure 28. Overall average ranking of all predictors and baselines for prokaryotic proteins in the DisProt dataset. 43
DisProt-PDB dataset 43
Supplementary Figure 29: Precision recall curve in the DisProt-PDB dataset. 44
Supplementary Figure 30. F1-score progress with threshold in the DisProt-PDB dataset. 45

Supplementary Figure 31. MCC progress with threshold in the DisProt-PDB dataset. 46
Supplementary Figure 32. Balanced accuracy progress with threshold in the DisProt-PDB dataset. 47
Supplementary Figure 33: FMax in the DisProt-PDB dataset. 48
Supplementary Figure 34: MCC in the DisProt-PDB dataset. 48
Supplementary Figure 35: Balanced accuracy in the DisProt-PDB dataset. 49
Supplementary Figure 36: MCC per protein in the DisProt-PDB dataset. 50
Supplementary Figure 37: FMax per protein in the DisProt-PDB dataset. 50
Supplementary Figure 38. Overall average ranking of all predictors and baselines in the DisProt-PDB dataset. 51
Supplementary Figure 39. Overall average ranking of the 10 best ranking predictors and baselines in the DisProt-PDB dataset. 52
Mammals 53
Supplementary Figure 40. Prediction success and CPU times for the ten top-ranking disorder predictors for mammalian proteins in the DisProt-PDB dataset. 53
Supplementary Figure 41: FMax for mammalian proteins in the DisProt-PDB dataset. 54
Supplementary Figure 42. F1-Score progress with threshold for mammalian proteins in the DisProt-PDB dataset. 55
Supplementary Figure 43: FMax per protein for mammalian proteins in the DisProt-PDB dataset. 56
Supplementary Figure 44. Overall average ranking of all predictors and baselines for mammalian proteins in the DisProt-PDB dataset. 57
Prokaryotes 58
Supplementary Figure 45. Prediction success and CPU times for the ten top-ranking disorder predictors for prokaryotic proteins in the DisProt-PDB dataset. 58
Supplementary Figure 46: FMax for prokaryotic proteins in the DisProt-PDB dataset. 59
Supplementary Figure 47. F1-Score progress with threshold for prokaryotic proteins in the DisProt-PDB dataset. 60
Supplementary Figure 48: FMax per protein for prokaryotic proteins in the DisProt-PDB dataset. 61
Supplementary Figure 49. Overall average ranking of all predictors and baselines for prokaryotic proteins in the DisProt-PDB dataset. 62
Fully Disordered Proteins 63
Supplementary Figure 50. Fraction of disordered residues in fully disordered proteins. 63
Binding 63
Supplementary Figure 51. Type of bindings in the DisProt-Binding dataset. 63
Supplementary Figure 52. Annotation overlap between resources annotating binding. 64
DisProt-Binding dataset 64
Supplementary Figure 53: Precision recall curve in the DisProt-Binding dataset. 64
Supplementary Figure 54. F1-score progress with threshold in the DisProt-Binding dataset. 65
Supplementary Figure 55. MCC progress with threshold in the DisProt-Binding dataset. 66
Supplementary Figure 56. Balanced accuracy progress with threshold in the DisProt-Binding dataset. 67
Supplementary Figure 57: FMax in the DisProt-Binding dataset. 68
Supplementary Figure 58: MCC in the DisProt-Binding dataset. 69
Supplementary Figure 59: Balanced accuracy in the DisProt-Binding dataset. 69

Supplementary Figure 60: MCC per protein in the DisProt-Binding dataset. 70
Supplementary Figure 61: FMax per protein in the DisProt-Binding dataset. 70
Supplementary Figure 62. Overall average ranking of all predictors and baselines. 71
Mammals 71
Supplementary Figure 63. Prediction success and CPU times for the ten top-ranking disorder predictors for mammalian proteins in the DisProt-Binding dataset. 72
Supplementary Figure 64: FMax for mammalian proteins in the DisProt-Binding proteins. 73
Supplementary Figure 65. F1-Score progress with threshold for mammalian proteins in the DisProt-Binding dataset. 74
Supplementary Figure 66: FMax per proteins for mammalian proteins in the DisProt-Binding dataset. 75
Supplementary Figure 67. Overall average ranking of all predictors and baselines for mammalian proteins in the DisProt-Binding dataset. 76
Prokaryotes 77
Supplementary Figure 68. Prediction success and CPU times for the ten top-ranking disorder predictors for prokaryotic proteins in the DisProt-Binding dataset. 77
Supplementary Figure 69: FMax for prokaryotic proteins in the DisProt-Binding dataset. 78
Supplementary Figure 70. F1-Score progress with threshold for prokaryotic proteins in the DisProt-Binding dataset. 79
Supplementary Figure 71: FMax per protein for prokaryotic proteins in the DisProt-Binding dataset. 80
Supplementary Figure 72. Overall average ranking of all predictors and baselines for prokaryotic proteins in the DisProt-Binding dataset. 80

Tables

Prediction methods

Method	Principal Investigator	Keywords	Publication (PubMed ID)
AUCpreD	Jinbo Xu	sequence labeling, deep conditional neural fields, conditional random fields, deep convolutional neural networks, AUC-maximization	27587688
AUCpreD_np	Jinbo Xu	sequence labeling, deep conditional neural fields, conditional random fields, deep convolutional neural networks, AUC-maximization	27587688
DFLpred	Lukasz Kurgan	disordered linkers, logistic regression, sliding window	27307636
DisEMBL-465	Rob B. Russell	Coils, missing coordinates, target selection	14604535
DisEMBL-HL	Rob B. Russell	Coils, missing coordinates, loops	14604535
DisoMine	Wim Vranken	Protein disorder prediction, recurrent neural network, biophysical features	n/a
DISOPRED-3.1	David T Jones	Meta-predictor, neural network, long IDRs	25391399
DisPredict2	Tamjidul Hoque	PSEE, contact energy, burial	27588752
DynaMine	Wim Vranken	Protein backbone dynamics prediction, linear regression, biophysical information	24225580
ESpritz-D	Silvio Tosatto	neural network, fast, DisProt	22190692
ESpritz-N	Silvio Tosatto	neural network, fast, NMR	22190692
ESpritz-X	Silvio Tosatto	neural network, fast, xray	22190692
fIDPlr	Lukasz Kurgan	meta-predictor, disorder function, binding, sliding window	n/a
fIPDnn	Lukasz Kurgan	meta-predictor, disorder function, binding	n/a
FoldUnfold	Oxana V. Galzitskaya	intrinsically flexibility, size of window, threshold	17021161
GlobPlot	Toby J. Gibson	Globularity, propensity, secondary structure, missing electron densities	12824398
IsUnstruct	Oxana V. Galzitskaya	residual potentials, boundary energy, disordered patterns, two-state model	21572175
IUpred-long	Zsuzsanna Dosztányi	energy estimation, statistical potential, fast	15955779
IUpred-short	Zsuzsanna Dosztányi	energy estimation, statistical potential, fast	15955779
IUpred2A-long	Zsuzsanna Dosztányi	energy estimation, statistical potential, fast	29860432
IUpred2A-short	Zsuzsanna Dosztányi	energy estimation, statistical potential, fast	29860432
JRONN	Robert Esnouf	Regional order neural network	15947016
MobiDB-lite	Silvio Tosatto	Meta-predictor, fast, InterPro	28453683
Predisorder	Jianlin Cheng	recurrent neural networks, disorder prediction	20025768

pyHCA	Isabelle Callebaut	Hydrophobic cluster, residue physico chemical properties, sequence topology, SVC	n/a
rawMSA	Björn Wallner	deep network, bidirectional neural network, embeddings, multiple sequence alignment	31415569
s2D-1	Michele Vendruscolo	Secondary structure, intrinsic disorder, NMR spectroscopy, sequence-based predictions	25534081
s2D-2	Michele Vendruscolo	Secondary structure, intrinsic disorder, NMR spectroscopy, sequence-based predictions	n/a
SPOT-Disorder1	Yaoqi Zhou	bidirectional recurrent neural network, LSTM	28011771
SPOT-Disorder-Single	Yaoqi Zhou	single-sequence, deep neural network	28011771
SPOT-Disorder2	Yaoqi Zhou	IncReSeNet	32173600
VSL2B	Zoran Obradovic	SVM, DisProt, xray	16618368

Supplementary Table 1. Disorder predictors
Participating methods for disorder region prediction grouped according to the Principal Investigator.

**Method**	**Principal Investigator**	**Keywords**	**Publication (PubMed ID)**
ANCHOR	Zsuzsanna Dosztányi	energy estimation, statistical potential, fast, disorder-to-order transition, energy gain	19717576
ANCHOR-2	Zsuzsanna Dosztányi	energy estimation, statistical potential, fast, disorder-to-order transition, energy gain	29860432
DISOPRED-3.1 binding	David T. Jones	Meta-predictor, machine-learning, long IDRs, SVM	25391399
DisoRDPbind_all	Lukasz Kurgan	disorder function, nucleic acids, DNA-binding, RNA-binding, protein-binding	26109352
DisoRDPbind_dna	Lukasz Kurgan	disorder function, nucleic acids, DNA-binding, RNA-binding, protein-binding	26109352
DisoRDPbind_prot	Lukasz Kurgan	disorder function, nucleic acids, DNA-binding, RNA-binding, protein-binding	26109352
DisoRDPbind_rna	Lukasz Kurgan	disorder function, nucleic acids, DNA-binding, RNA-binding, protein-binding	26109352
fMoRFpred	Lukasz Kurgan	MoRF, induced folding, molecular recognition features, sliding window	26651072
MorfChibi-light	Joerg Gsponer	Molecular recognition features (MoRFs), Support Vector Machines, Hierarchical learning	27174932
MorfChibi-web	Joerg Gsponer	Molecular recognition features (MoRFs), Support Vector Machines, Hierarchical learning, Conservation	27174932
OPAL	Alok Sharma	Molecular Recognition Features, Intrinsically Disordered Proteins, Support Vector Machine, BigramMoRF and StructMoRF	29360926

Supplementary Table 2. Binding predictors

Participating methods for disorder binding site prediction grouped according to the Principal Investigator.

Method (Principal Investigator)	Description
**ANCHOR (Zsuzsanna Dosztányi)**	ANCHOR is a fast method that predicts disordered regions that can undergo a disorder-to-order transition upon binding to globular protein partners. To predict such regions ANCHOR aims to capture the general disorder tendency of these regions and their ability to gain energy by interacting with a more structured environment using the estimated energy approach. Most of the parameters are based on known protein structures and only five parameters were optimized on known disordered binding regions, lending the methods its robustness.
**ANCHOR2 (Zsuzsanna Dosztányi)**	ANCHOR2 retains the original idea behind ANCHOR and employs a simple biophysics-based model to predict disordered binding regions in a similar way. This approach relies on a larger collection of known disordered binding regions to improve on the modeling of the structured environment of these regions.
**AUCpreD (Jinbo Xu)**	The AUCpreD method developed by Xu group formulates IDR prediction as a sequence labeling problem and employs a machine learning technique called Deep Convolutional Neural Fields (DeepCNF) to solve it. DeepCNF is an integration of deep convolutional neural networks (DCNN) and conditional random fields (CRF). DeepCNF can model not only complex sequence-structure relationships in a hierarchical manner, but also the correlation of order and disorder labels among adjacent residues. AUCpredD can predict IDRs from not only sequence profiles (i.e., evolutionary information), but also primary sequence. Predicting from a primary sequence is not as accurate as predicting from a sequence profile, but much faster. The distribution of ordered and disordered residues is highly imbalanced (about 15:1), which makes it challenging to accurately predict IDRs using traditional machine learning approaches. To deal with this, AUCpredD is trained by maximizing area under the ROC curve (AUC), which is an unbiased measure for class-imbalanced data. Four publicly available datasets are used to train, validate and evaluate AUCpreD. In particular, the UniProt90 dataset released before May 01, 2010 is used to train and validate the model parameters. The CASP9, CASP10 and CAMEO test proteins are used to evaluate prediction accuracy. Sequence identity 25% is employed to remove redundancy between training and test data. In total, there are 13,800 training and validation proteins and a 10-fold cross-validation is performed to train 10 different models, which are then combined to form the final prediction model.
**AUCpreD_no-profile (Jinbo Xu)**	The AUCpreD method developed by Xu group formulates IDR prediction as a sequence labeling problem and employs a machine learning technique called Deep Convolutional Neural Fields (DeepCNF) to solve it. DeepCNF is an integration of deep convolutional neural networks (DCNN) and conditional random fields (CRF). DeepCNF can model not only complex sequence-structure relationships in a hierarchical manner, but also the correlation of order and disorder labels among adjacent residues. AUCpredD can predict IDRs from not only sequence profiles (i.e., evolutionary information), but also primary sequence. Predicting from a primary sequence is not as accurate as predicting from a sequence profile, but much faster. The distribution of ordered and disordered residues is highly imbalanced (about 15:1), which makes it challenging to accurately predict IDRs using traditional machine learning approaches. To deal with this, AUCpredD is trained by maximizing area under the ROC curve (AUC), which is an unbiased measure for class-imbalanced data. Four publicly available datasets are used to train, validate and evaluate AUCpreD. In particular, the UniProt90 dataset released before May 01, 2010 is used to train and validate the model parameters. The CASP9, CASP10 and CAMEO test

	proteins are used to evaluate prediction accuracy. Sequence identity 25% is employed to remove redundancy between training and test data. In total, there are 13,800 training and validation proteins and a 10-fold cross-validation is performed to train 10 different models, which are then combined to form the final prediction model.
**DFLpred (Lukasz Kurgan)**	Logistic regression based on custom-designed features extracted from putative disorder and propensity for helix and coil conformations. Fast and requires protein sequence as the only input, thus applicable to prediction at the whole genome scale.
**DisEMBL-465 (Rob B. Russel)**	DisEMBL is a computational tool for prediction of disordered/unstructured regions within a protein sequence. As no clear definition of disorder exists, we have developed parameters based on several alternative definitions, and introduced a new one based on the concept of ``hot loops'', i.e. coils with high temperature factors. DisEMBL is useful for target selection and the design of constructs as needed for many biochemical studies, particularly structural biology and structural genomics projects.
**DisEMBL-HL (Rob B. Russel)**	DisEMBL is a computational tool for prediction of disordered/unstructured regions within a protein sequence. As no clear definition of disorder exists, we have developed parameters based on several alternative definitions, and introduced a new one based on the concept of ``hot loops'', i.e. coils with high temperature factors. DisEMBL is useful for target selection and the design of constructs as needed for many biochemical studies, particularly structural biology and structural genomics projects.
**DisoMine (Wim Vranken)**	DisoMine predicts protein disorder with recurrent neural networks from simple predictions of protein dynamics, secondary structure and early folding. The tool is fast and requires only a single sequence, making it applicable for large-scale screening, including poorly studied and orphan proteins.
**DISOPRED-3.1 (David T. Jones)**	DisoPred3 first identifies disordered residues through a consensus of the output generated by DISOPRED2 and two additional machine-learning based modules trained on large IDRs, and then annotates them as protein binding through an additional SVM classifier
**DISOPRED-3.1_binding (David T. Jones)**	DISOPRED3.1 binding finds binding regions in disordered regions predicted by DISOPRED3. It’s based on an SVM classifier. Using a sliding window of size 15, we derived three independent SVM classifiers from the training data that are based on (i) single sequences alone; (ii) PSSM values obtained after three search iterations of PSI-BLAST against UniRef90; (iii) the same PSSM scores, followed by the length of input region.
**DisoRDPbind_all (Lukasz Kurgan)**	Logistic regression based on custom-designed features extracted from putative disorder, putative secondary structure, sequence complexity profile, and selected physicochemical properties of residues. Regression predictions are combined with results of the alignment into a dataset of functionally annotated disordered regions. Fast and requires protein sequence as the only input, thus applicable to prediction at the whole genome scale.
**DisoRDPbind_dna (Lukasz Kurgan)**	Logistic regression based on custom-designed features extracted from putative disorder, putative secondary structure, sequence complexity profile, and selected physicochemical properties of residues. Regression predictions are combined with results of the alignment into a dataset of functionally annotated disordered regions. Fast and requires protein sequence as the only input, thus applicable to prediction at the whole genome scale.
**DisoRDPbind_prot (Lukasz Kurgan)**	Logistic regression based on custom-designed features extracted from putative disorder, putative secondary structure, sequence complexity profile, and selected physicochemical properties of residues. Regression predictions are combined with results of the alignment into a dataset of functionally annotated disordered regions. Fast and requires protein sequence as the only input, thus applicable to prediction at the whole genome scale.

**DisoRDPbind_rna (Lukasz Kurgan)**	Logistic regression based on custom-designed features extracted from putative disorder, putative secondary structure, sequence complexity profile, and selected physicochemical properties of residues. Regression predictions are combined with results of the alignment into a dataset of functionally annotated disordered regions. Fast and requires protein sequence as the only input, thus applicable to prediction at the whole genome scale.
**DisPredict2 (Md Tamjidul Hoque)**	DisPredic2 uses position specific estimated energy, named PSEE, for each residue of a protein based on sequence information alone. The quantification of PSEE includes the interaction effect of the target residue within a neighborhood in terms of pairwise contact energies between different amino acid types. Neighborhood size is estimated in terms of the number of residues on either side of the target residue with which it can form favorable contacts. Furthermore, it utilizes the predicted relative exposure (or burial) of a residue to approximate the local three-dimensional conformational position and stability of the residue.
**DynaMine (Wim Vranken)**	DynaMine is a fast, high-quality predictor of protein backbone dynamics from single protein sequences. DynaMine is trained on information derived from experimental NMR chemical shift data for proteins in solution, and can identify disordered regions within proteins without depending on prior disorder knowledge or three-dimensional structural information.
**ESpritz-D (Silvio Tosatto)**	ESpritz combines a sophisticated BRNN architecture with enhanced definitions of disorder flavors. This version is based on DisProt2. The BRNN improves performance in general compared to previous iterations of this predictor (Spritz3 and CSpritz4) and especially on this training-set.
**ESpritz-N (Silvio Tosatto)**	ESpritz combines a sophisticated BRNN architecture with enhanced definitions of disorder flavors. This is based on NMR mobility calculated on NMR conformers from PDB. The BRNN improves performance in general compared to previous iterations of this predictor (Spritz3 and CSpritz4) and substantially on this training-set.
**ESpritz-X (Silvio Tosatto)**	ESpritz combines a sophisticated BRNN architecture with enhanced definitions of disorder flavors. This version is based on X-ray structures from PDB. The BRNN improves performance in general compared to previous iterations of this predictor (Spritz3 and CSpritz4) and slightly on this training-set.
**fIDPlr (Lukasz Kurgan)**	Logistic regression model trained with a comprehensive set of custom-designed features extracted from predicted disorder and disorder function predicted with DFLpred, DisoRDPbind and fMoRFpred and pre-processed using wrapper-based feature selection. Fast and requires protein sequence as the only input, thus applicable to prediction at the whole genome scale.
**fIPDnn (Lukasz Kurgan)**	Dense and deep neural network trained with a comprehensive feature set extracted from predicted disorder and disorder function predicted with DFLpred, DisoRDPbind and fMoRFpred. Fast and requires protein sequence as the only input, thus applicable to prediction at the whole genome scale.
**fMoRFpred (Lukasz Kurgan)**	Support Vector Machine (SVM) based on custom-designed features extracted from putative intrinsic disorder, putative secondary structure, estimated B-factor and physicochemical characteristics including structural stability and unfolding energy. Fast and requires protein sequence as the only input, thus applicable to prediction at the whole genome scale.
**FoldUnfold (Oxana V. Galzitskaya)**	A new parameter, namely, the average packing density of the residues, was introduced to detect disordered regions in the protein sequence. We showed that regions with a low expected packing density will be responsible for the appearance of disordered regions.
**GlobPlot (Toby J Gibson)**	GlobPlot identifies regions of globularity and disorder within protein sequences based on a running sum of the propensity for amino acids to be in an ordered or disordered state. The GlobPlot package currently contains seven different propensity sets, as for example

	tendency to form secondary structure and missing electron densities in X-Ray experiments.
**IsUnstruct (Oxana V. Galzitskaya)**	The Ising model is used. The energy of transfer to the unfolded state is attributed to each residue, and the energy of the boundary between the folded and unfolded states is also taken into account. These energies were optimally selected based on PDB.
**IUPred-long (Zsuzsanna Dosztányi)**	IUPred-long predicts protein disorder based on an energy estimation approach utilizing statistical potentials. The parameters of the method are derived from a collection of known structures only and disordered regions are recognized based on their unfavorable estimated energies. IUPred is a fast and robust method that carries out predictions for single protein sequences without using evolutionary information.
**IUPred-short (Zsuzsanna Dosztányi)**	IUPred-short is based on the same approach as IUPred-long but choices of window sizes are slightly tailored towards predicting missing residues from PDB structures.
**IUPred2A-long (Zsuzsanna Dosztányi)**	IUPred2A-long is an implementation of IUPred-long in PYTHON with minor bug fixes.
**IUPred2A-short (Zsuzsanna Dosztányi)**	IUPred2A-short is an implementation of IUPred-short in PYTHON with minor bug fixes.
**JRONN (Robert Esnouf)**	Regional order neural network (RONN) software as an application of our recently developed 'bio-basis function neural network' pattern recognition algorithm to the detection of natively disordered regions in proteins. The decision about the likelihood of disorder is based on alignments to an ensemble of sequences of known folding state.
**MobiDB-lite (Silvio Tosatto)**	MobiDB-lite is a meta-predictor that combines the results of 8 highly orthogonal disorder predictors in a consensus. A post processing phase smooths a strict majority consensus. Finally, predicted regions shorter than 20 residues are filtered out.
**MorfChibi-light (Joerg Gsponer)**	MoRFchibi_light scores are computed using Hierarchical Learning [PMID: 26517836, 30952844]. Specifically, the scores of two modules (MoRFchibi and ESpritz) are combined hierarchically using Bayes rule to generate the MoRFchibi_web score. The MoRFchibi module is assembled hierarchically from two support vector machines that utilize RBF and Sigmoid kernels and are designed to identify MoRFs based on the contrast of physicochemical properties of MoRFs and their flanking regions. ESpritz1 is used with the (D) option parameter to predict long disordered protein regions. The hierarchical structure of the predictor generates more balanced scores such that MoRF sequences used in the training will not get too high scores that overshadow or obfuscate the ones of novel MoRF sequences.
**MorfChibi-web (Joerg Gsponer)**	MoRFchibi_web scores are computed using Hierarchical Learning [PMID: 26517836, 30952844]. Specifically, the scores of three modules (MoRFchibi, ESpritz, and ICS) are combined hierarchically using Bayes rule to generate the MoRFchibi_web score. The MoRFchibi module is assembled hierarchically from two support vector machines that utilize RBF and Sigmoid kernels and are designed to identify MoRFs based on the contrast of physicochemical properties of MoRFs and their flanking regions. ESpritz1 is used with the (D) option parameter to predict long disordered protein regions. ICS predicts MoRF regions based on conservation information using PSI-BLAST PSSM matrices. The hierarchical structure of the predictor generates more balanced scores such that MoRF sequences used in the training will not get too high scores that overshadow or obfuscate the ones of novel MoRF sequences.

**OPAL (Alok Sharma)**	OPAL is an ensemble of two predictors, MoRFchibi and PROMIS. PROMIS is trained using the structural information of the disordered protein sequences and MoRFchibi uses the physicochemical properties of the disordered protein sequences. StructMoRF and BigramMoRF framework is used to extract features from the MoRF residues and the neighboring amino acids upstream and downstream of the MoRF region. Successive feature selection scheme in the forward direction is performed and a support vector machine (SVM) classifier is used for prediction. OPAL provided a significant performance improvement over the benched marked predictors. The datasets used are obtained from Disfani et al.,(2012) and Malhis et al., (2015). TRAIN set is used to train the model; TEST set is used for evaluation and EXP53 set for validating the performance.
**PreDisorder (Jianlin Cheng)**	The bidirectional recurrent neural networks are used to predict disordered residues. The predicted disorder probabilities are scaled to make the ratio of predicted disordered residues is similar to that in the training data.
**pyHCA (Isabelle Callebaut)**	The pyHCA disorder predictor uses a topology-based approach considering hydrophobic and hydrophilic runs within HCA-based hydrophobic clusters. The approach only uses the information contained in a single protein sequence, i.e. without homology information such as that provided by MSAs. Physico-chemical features were extracted from the hydrophobic clusters and used to train a support vector classifier for the residue disordered states. Training was setup using a 5 fold cross-validation methodology on a splitted Disprot7 database (train/validation/test, 0.8/0.1/0.1).The set of features was selected based on prediction comparisons on the validation set. This approach is particularly well suited for small datasets or proteins without homologs as no large datasets are required for training.
**rawMSA (Björn Wallner)**	rawMSA is a suite of methods for the prediction of structural features of proteins. Here, the input is not a set of pre-determined features (such as evolutionary profiles or predicted secondary structure) as is common in classical ML-based methods. Instead, the whole MSA is used as a textual input to the neural network so that the evolutionary information is not compressed in any way and the feature extraction can be automatically performed by the deep network. The mapping between amino acid letters to floating point vectors is done in the first layer of the deep network with an embedding layer, as is done in Natural Language Processing (NLP) techniques. The input is not split into windows, and all predictions are done at the same time for all input amino acids so that long disordered / ordered regions are not split up at prediction stage.
**s2D (Michele Vendruscolo)**	s2D predicts secondary-structure populations from amino acid sequences, which simultaneously characterizes structure and disorder in a unified statistical mechanics framework. This method is based on advances made in the analysis of NMR chemical shifts that provide quantitative information about the probability distributions of secondary-structure elements in disordered states. s2D predicts secondary-structure populations with an average error of about 14%.
**s2D-2 (Michele Vendruscolo)**	s2D-2 is a re-trained and improved version of the s2D predictor.
**SPOT-Disorder1 (Yaoqi Zhou)**	SPOT-Disorder implements deep bidirectional LSTM recurrent neural networks in the problem of protein intrinsic disorder prediction. Its results improve on a similar method using a traditional, window-based neural network (SPINE-D) in all datasets tested without separate training on short and long disordered regions.
**SPOT-Disorder-Single (Yaoqi Zhou)**	SPOT-Disorder single is based on the same architecture of SPOT-Disorder a deep bidirectional LSTM recurrent neural networks. However applies a single threshold to prediction scores.

**SPOT-Disorder2 (Yaoqi Zhou)**	SPOT-Disorder2 improves on SPOT-Disorder by the use of an ensemble of IncReSeNet, LSTM, and FC network topologies, rather than a single LSTM topology in the previous version. Further advancements were: multiple inception-style pathways and signal Squeeze-and-Excitation, an updated feature set from our previous work to include the latest state-of-the-art predictions for protein secondary structure from SPOT-1D.
**VSL2B (Zoran Obradovic)**	PONDR® VSL2 predictor is a combination of neural network predictors for both short and long disordered regions. A length limit of 30 residues divides short and long disordered regions. Each individual predictor is trained by the dataset containing sequences of that specific length. The final prediction is a weighted average determined by a second layer predictor. PONDR® VSL2 applies not only to the sequence profile, but also the result of sequence alignments from PSI-BLAST and secondary structure prediction from PHD and PSIPRED. This predictor is so far the most accurate predictor in the PONDR® family.

Supplementary Table 3. Predictors description
Name, Principal Investigator authors and brief description of all participating methods for disorder and binding site prediction.

DisProt dataset

Evaluation Results

	BAC	F1-S	FPR	MCC	PPV	TPR	TNR	COV
SPOT-Disorder2	0.712	0.486	0.325	0.308	0.447	0.734	0.675	610
SPOT-Disorder1	0.706	0.475	0.368	0.295	0.424	0.761	0.632	644
RawMSA	0.692	0.449	0.281	0.290	0.439	0.655	0.719	646
AUCpreD	0.704	0.466	0.370	0.283	0.409	0.762	0.630	644
DISOPRED-3.1	0.674	0.427	0.319	0.267	0.421	0.665	0.681	646
Predisorder	0.671	0.429	0.260	0.263	0.425	0.601	0.740	642
IUPred2A-short	0.674	0.424	0.280	0.256	0.407	0.625	0.720	646
IUPred-short	0.675	0.424	0.285	0.256	0.405	0.632	0.715	645
AUCpreD-np	0.681	0.441	0.328	0.254	0.401	0.679	0.672	646
SPOT-Disorder-Single	0.676	0.440	0.349	0.251	0.401	0.686	0.651	646
MobiDB-lite	0.668	0.423	0.289	0.247	0.400	0.619	0.711	645
fIDPnn	0.668	0.440	0.308	0.247	0.416	0.627	0.692	645
IsUnstruct	0.667	0.425	0.304	0.244	0.401	0.632	0.696	646
IUPred-long	0.654	0.395	0.263	0.243	0.432	0.569	0.737	645
ESpritz-X	0.669	0.427	0.327	0.241	0.388	0.658	0.673	645
VSL2B	0.663	0.421	0.320	0.240	0.395	0.639	0.680	644
IUPred2A-long	0.654	0.396	0.268	0.240	0.424	0.573	0.732	646
JRONN	0.657	0.404	0.282	0.238	0.402	0.598	0.718	645
ESpritz-N	0.647	0.389	0.237	0.236	0.413	0.539	0.763	645
fIDPlr	0.647	0.409	0.297	0.220	0.420	0.575	0.703	645

DynaMine	0.654	0.400	0.325	0.220	0.373	0.630	0.675	645
DisoMine	0.643	0.420	0.410	0.206	0.388	0.673	0.590	646
**PDB Close**	0.598	0.383	0.365	0.199	0.404	0.561	0.635	604
PyHCA	0.640	0.390	0.345	0.198	0.370	0.618	0.655	646
S2D-1	0.610	0.350	0.280	0.190	0.377	0.514	0.720	644
**Gene3D**	0.630	0.405	0.474	0.188	0.380	0.709	0.526	652
DisEMBL-465	0.608	0.357	0.218	0.180	0.383	0.446	0.782	644
S2D-2	0.618	0.365	0.398	0.173	0.336	0.634	0.602	644
**PDB Remote**	0.600	0.337	0.403	0.172	0.343	0.607	0.597	530
FoldUnfold	0.620	0.386	0.382	0.169	0.383	0.607	0.618	621
ESpritz-D	0.632	0.400	0.435	0.167	0.359	0.672	0.565	645
**PDB observed**	0.609	0.428	0.507	0.164	0.408	0.697	0.493	652
GlobPlot	0.570	0.300	0.230	0.136	0.353	0.397	0.770	645
DisEMBL-HL	0.563	0.269	0.124	0.135	0.371	0.284	0.876	644
**Conservation**	0.558	0.285	0.380	0.116	0.332	0.516	0.620	652
DisPredict-2	0.557	0.310	0.437	0.060	0.300	0.530	0.563	646
DFLpred	0.473	0.033	0.009	0.020	0.286	0.022	0.991	646

Supplementary Table 4. Per-protein classification performance in the DisProt dataset
Performance of predictors and baselines for *DisProt* dataset. Metrics are averaged over targets (proteins), sorted by MCC and predictor thresholds are optimized on MCC. Baselines are shown in bold. COV is coverage, i.e. number of predicted target proteins.

	**BAC**	**F1-S**	**FPR**	**MCC**	**PPV**	**TPR**	**TNR**	**COV**
fIDPnn	0.720	0.483	0.189	0.370	0.392	0.629	0.811	645
SPOT-Disorder2	0.725	0.469	0.343	0.349	0.333	0.794	0.657	610
fIDPlr	0.693	0.452	0.184	0.330	0.374	0.570	0.816	645
SPOT-Disorder1	0.723	0.434	0.386	0.330	0.294	0.832	0.614	644
RawMSA	0.714	0.445	0.297	0.328	0.321	0.725	0.703	646
AUCpreD	0.712	0.433	0.376	0.318	0.297	0.801	0.624	644
SPOT-Disorder-Single	0.710	0.432	0.341	0.315	0.302	0.760	0.659	646
ESpritz-D	0.703	0.428	0.325	0.307	0.303	0.731	0.675	645
AUCpreD-np	0.699	0.424	0.327	0.301	0.300	0.725	0.673	646
Predisorder	0.691	0.435	0.280	0.301	0.324	0.661	0.720	642
DisoMine	0.698	0.424	0.326	0.299	0.300	0.721	0.674	646
IUPred2A-short	0.688	0.420	0.297	0.290	0.305	0.674	0.703	646
MobiDB-lite	0.688	0.420	0.296	0.289	0.305	0.673	0.704	645
IsUnstruct	0.689	0.418	0.311	0.288	0.300	0.688	0.689	646
ESpritz-X	0.689	0.418	0.309	0.288	0.301	0.686	0.691	645

IUPred-short	0.688	0.418	0.304	0.288	0.302	0.679	0.696	645
IUPred-long	0.686	0.418	0.294	0.287	0.305	0.666	0.706	645
IUPred2A-long	0.685	0.416	0.299	0.285	0.302	0.670	0.701	646
VSL2B	0.684	0.408	0.341	0.277	0.286	0.709	0.659	644
JRONN	0.672	0.401	0.318	0.263	0.287	0.663	0.682	645
ESpritz-N	0.664	0.400	0.271	0.259	0.300	0.599	0.729	645
DISOPRED-3.1	0.674	0.393	0.401	0.258	0.266	0.749	0.599	646
PyHCA	0.660	0.385	0.346	0.241	0.271	0.666	0.654	646
DynaMine	0.660	0.384	0.362	0.240	0.267	0.682	0.638	645
**Gene3D**	0.653	0.368	0.486	0.226	0.240	0.791	0.514	652
DisEMBL-465	0.627	0.363	0.215	0.214	0.296	0.468	0.785	644
FoldUnfold	0.642	0.365	0.382	0.211	0.251	0.666	0.618	621
S2D-1	0.633	0.361	0.329	0.203	0.259	0.595	0.671	644
**PDB Close**	0.637	0.353	0.380	0.202	0.242	0.655	0.620	604
S2D-2	0.624	0.347	0.439	0.183	0.232	0.687	0.561	644
**PDB observed**	0.616	0.339	0.565	0.174	0.215	0.796	0.435	652
DisEMBL-HL	0.577	0.286	0.099	0.172	0.330	0.253	0.901	644
**PDB Remote**	0.614	0.321	0.450	0.163	0.210	0.678	0.550	530
DisPredict-2	0.599	0.326	0.326	0.152	0.237	0.523	0.674	646
GlobPlot	0.587	0.312	0.253	0.143	0.246	0.427	0.747	645
**Conservation**	0.552	0.288	0.483	0.077	0.191	0.587	0.517	652
DFLpred	0.503	0.025	0.008	0.022	0.249	0.013	0.992	646

Supplementary Table 5. Per-residue classification performance in the *DisProt* dataset
Performance of predictors and baselines for *DisProt* dataset. Metrics are calculated over the whole dataset, sorted by MCC and predictors threshold are optimized on MCC. Baselines are shown in bold. COV is coverage, i.e. number of predicted target proteins.

Evaluation Results (Full ID)

Fully disordered proteins (ID fraction ≥80%)										
	**TN**	**FP**	**FN**	**TP**	**MCC**	**F1-S**	**TNR**	**TPR**	**PPV**	**BAC**
fIDPnn	569	21	24	32	0.549	0.587	0.964	0.571	0.604	0.768
fIDPlr	544	46	17	39	0.515	0.553	0.922	0.696	0.459	0.809
RawMSA	553	37	21	35	0.503	0.547	0.937	0.625	0.486	0.781
AUCpreD	568	22	28	28	0.487	0.528	0.963	0.500	0.560	0.731
SPOT-Disorder2	539	51	19	37	0.471	0.514	0.914	0.661	0.420	0.787
PyHCA	562	28	27	29	0.467	0.513	0.953	0.518	0.509	0.735
IUPred2A-long	564	26	28	28	0.464	0.509	0.956	0.500	0.519	0.728
ESpritz-N	565	25	29	27	0.455	0.500	0.958	0.482	0.519	0.720

SPOT-Disorder-Single	577	13	34	22	0.461	0.484	0.978	0.393	0.629	0.685
JRONN	558	32	28	28	0.432	0.483	0.946	0.500	0.467	0.723
Predisorder	528	62	19	37	0.434	0.477	0.895	0.661	0.374	0.778
IUPred2A-short	585	5	37	19	0.492	0.475	0.992	0.339	0.792	0.665
DisoMine	516	74	17	39	0.423	0.462	0.875	0.696	0.345	0.786
VSL2B	516	74	18	38	0.411	0.452	0.875	0.679	0.339	0.777
AUCpreD-np	577	13	36	20	0.428	0.449	0.978	0.357	0.606	0.668
MobiDB-lite	586	4	39	17	0.471	0.442	0.993	0.304	0.810	0.648
ESpritz-D	533	57	24	32	0.388	0.441	0.903	0.571	0.360	0.737
IsUnstruct	548	42	29	27	0.374	0.432	0.929	0.482	0.391	0.705
ESpritz-X	576	14	37	19	0.403	0.427	0.976	0.339	0.576	0.658
DisPredict-2	538	52	29	27	0.338	0.400	0.912	0.482	0.342	0.697
**Gene3D**	464	126	11	45	0.376	0.396	0.786	0.804	0.263	0.795
DisEMBL-HL	580	10	43	13	0.327	0.329	0.983	0.232	0.565	0.608
FoldUnfold	451	139	19	37	0.269	0.319	0.764	0.661	0.210	0.713
**PDB observed**	407	183	13	43	0.270	0.305	0.690	0.768	0.190	0.729
S2D-2	454	136	23	33	0.230	0.293	0.769	0.589	0.195	0.679
DISOPRED-3.1	568	22	44	12	0.223	0.267	0.963	0.214	0.353	0.588
**Conservation**	175	415	18	38	-0.015	0.149	0.297	0.679	0.084	0.488
DisEMBL-465	590	0	52	4	0.256	0.133	1.000	0.071	1.000	0.536
**PDB Close**	540	50	49	7	0.040	0.124	0.915	0.125	0.123	0.520
**PDB Remote**	542	48	51	5	0.008	0.092	0.919	0.089	0.094	0.504
GlobPlot	589	1	55	1	0.082	0.034	0.998	0.018	0.500	0.508
DFLpred	590	0	56	0	0.000	0.000	1.000	0.000	0.000	0.500
DynaMine	590	0	56	0	0.000	0.000	1.000	0.000	0.000	0.500

Supplementary Table 6. Detection of proteins with more than 80% of disorder
Performance of predictors and baselines in the task of identifying fully disordered proteins sorted by F1-Score. Where a fully disordered protein is defined as protein with 80% or more residues annotated / predicted as disordered. Baselines are shown in bold.

Fully disordered proteins (ID fraction ≥90%)										
	**TN**	**FP**	**FN**	**TP**	**MCC**	**F1-S**	**TNR**	**TPR**	**PPV**	**BAC**
fIDPnn	580	17	19	30	0.595	0.625	0.972	0.612	0.638	0.792
RawMSA	572	25	18	31	0.556	0.590	0.958	0.633	0.554	0.795
IUPred2A-long	590	7	28	21	0.542	0.545	0.988	0.429	0.750	0.708
fIDPlr	557	40	18	31	0.479	0.517	0.933	0.633	0.437	0.783
PyHCA	591	6	30	19	0.518	0.514	0.990	0.388	0.760	0.689
JRONN	588	9	29	20	0.503	0.513	0.985	0.408	0.690	0.697

Predisorder	576	21	25	24	0.473	0.511	0.965	0.490	0.533	0.727
SPOT-Disorder-Single	589	8	30	19	0.495	0.500	0.987	0.388	0.704	0.687
AUCpreD	585	12	29	20	0.474	0.494	0.980	0.408	0.625	0.694
ESpritz-N	588	9	30	19	0.485	0.494	0.985	0.388	0.679	0.686
VSL2B	556	41	20	29	0.446	0.487	0.931	0.592	0.414	0.762
DisoMine	540	57	17	32	0.428	0.464	0.905	0.653	0.360	0.779
IsUnstruct	579	18	29	20	0.425	0.460	0.970	0.408	0.526	0.689
SPOT-Disorder2	562	35	24	25	0.412	0.459	0.941	0.510	0.417	0.726
**Gene3D**	499	98	10	39	0.409	0.419	0.836	0.796	0.285	0.816
ESpritz-D	546	51	24	25	0.349	0.400	0.915	0.510	0.329	0.712
MobiDB-lite	594	3	36	13	0.443	0.400	0.995	0.265	0.812	0.630
DisPredict-2	573	24	31	18	0.351	0.396	0.960	0.367	0.429	0.664
ESpritz-X	589	8	35	14	0.398	0.394	0.987	0.286	0.636	0.636
AUCpreD-np	584	13	34	15	0.370	0.390	0.978	0.306	0.536	0.642
IUPred2A-short	593	4	37	12	0.406	0.369	0.993	0.245	0.750	0.619
S2D-2	538	59	28	21	0.265	0.326	0.901	0.429	0.262	0.665
**PDB observed**	449	148	13	36	0.286	0.309	0.752	0.735	0.196	0.743
DisEMBL-HL	596	1	40	9	0.390	0.305	0.998	0.184	0.900	0.591
FoldUnfold	455	142	15	34	0.271	0.302	0.762	0.694	0.193	0.728
DISOPRED-3.1	589	8	41	8	0.255	0.246	0.987	0.163	0.500	0.575
DisEMBL-465	597	0	46	3	0.238	0.115	1.000	0.061	1.000	0.531
**Conservation**	314	283	29	20	-0.035	0.114	0.526	0.408	0.066	0.467
**PDB Remote**	576	21	45	4	0.064	0.108	0.965	0.082	0.160	0.523
**PDB Close**	576	21	46	3	0.036	0.082	0.965	0.061	0.125	0.513
DynaMine	597	0	49	0	0.000	0.000	1.000	0.000	0.000	0.500
GlobPlot	597	0	49	0	0.000	0.000	1.000	0.000	0.000	0.500
DFLpred	597	0	49	0	0.000	0.000	1.000	0.000	0.000	0.500

Supplementary Table 7. Detection of proteins with more than 90% of disorder
Performance of predictors and baselines in the task of identifying fully disordered proteins sorted by F1-Score. Where a fully disordered protein is defined as protein with 90% or more residues annotated / predicted as disordered. Baselines are shown in bold.

Fully disordered proteins (ID fraction ≥99%)										
	**TN**	**FP**	**FN**	**TP**	**MCC**	**F1-S**	**TNR**	**TPR**	**PPV**	**BAC**
RawMSA	588	13	20	25	0.578	0.602	0.978	0.556	0.658	0.767
fIDPnn	587	14	22	23	0.534	0.561	0.977	0.511	0.622	0.744
VSL2B	583	18	22	23	0.502	0.535	0.970	0.511	0.561	0.741
fIDPlr	572	29	20	25	0.467	0.505	0.952	0.556	0.463	0.754
DisoMine	557	44	18	27	0.429	0.466	0.927	0.600	0.380	0.763

Predisorder	596	5	30	15	0.478	0.462	0.992	0.333	0.750	0.663
IsUnstruct	593	8	30	15	0.440	0.441	0.987	0.333	0.652	0.660
AUCpreD	589	12	29	16	0.420	0.438	0.980	0.356	0.571	0.668
SPOT-Disorder2	576	25	26	19	0.385	0.427	0.958	0.422	0.432	0.690
IUPred2A-long	598	3	32	13	0.465	0.426	0.995	0.289	0.812	0.642
SPOT-Disorder-Single	594	7	31	14	0.430	0.424	0.988	0.311	0.667	0.650
ESpritz-N	599	2	33	12	0.460	0.407	0.997	0.267	0.857	0.632
**Gene3D**	505	96	10	35	0.391	0.398	0.840	0.778	0.267	0.809
ESpritz-D	558	43	24	21	0.337	0.385	0.928	0.467	0.328	0.698
DisPredict-2	594	7	33	12	0.384	0.375	0.988	0.267	0.632	0.628
MobiDB-lite	599	2	35	10	0.413	0.351	0.997	0.222	0.833	0.609
**PDB observed**	493	108	13	32	0.328	0.346	0.820	0.711	0.229	0.766
JRONN	597	4	35	10	0.377	0.339	0.993	0.222	0.714	0.608
ESpritz-X	597	4	36	9	0.351	0.310	0.993	0.200	0.692	0.597
AUCpreD-np	591	10	35	10	0.302	0.308	0.983	0.222	0.500	0.603
FoldUnfold	456	145	14	31	0.256	0.281	0.759	0.689	0.176	0.724
IUPred2A-short	600	1	38	7	0.354	0.264	0.998	0.156	0.875	0.577
S2D-2	597	4	38	7	0.293	0.250	0.993	0.156	0.636	0.574
PyHCA	601	0	39	6	0.354	0.235	1.000	0.133	1.000	0.567
DISOPRED-3.1	599	2	41	4	0.227	0.157	0.997	0.089	0.667	0.543
DisEMBL-HL	601	0	42	3	0.250	0.125	1.000	0.067	1.000	0.533
DisEMBL-465	601	0	43	2	0.204	0.085	1.000	0.044	1.000	0.522
**PDB Close**	598	3	43	2	0.115	0.080	0.995	0.044	0.400	0.520
**PDB Remote**	592	9	43	2	0.058	0.071	0.985	0.044	0.182	0.515
**Conservation**	590	11	43	2	0.047	0.069	0.982	0.044	0.154	0.513
DynaMine	601	0	45	0	0.000	0.000	1.000	0.000	0.000	0.500
GlobPlot	601	0	45	0	0.000	0.000	1.000	0.000	0.000	0.500
DFLpred	601	0	45	0	0.000	0.000	1.000	0.000	0.000	0.500

Supplementary Table 8. Detection of proteins with more than 99% of disorder
Performance of predictors and baselines in the task of identifying fully disordered proteins sorted by F1-Score. Where a fully disordered protein is defined as protein with 99% or more residues annotated / predicted as disordered. Baselines are shown in bold.

DisProt-PDB dataset

Evaluation Results

	BAC	F1-S	FPR	MCC	PPV	TPR	TNR	COV
**PDB observed**	0.817	0.751	0.260	0.426	0.847	0.844	0.740	652

SPOT-Disorder2	0.753	0.643	0.106	0.356	0.745	0.651	0.894	610
**PDB Close**	0.716	0.624	0.291	0.341	0.780	0.712	0.709	604
AUCpreD	0.738	0.623	0.122	0.334	0.719	0.646	0.878	644
SPOT-Disorder1	0.761	0.649	0.162	0.330	0.718	0.690	0.838	644
DISOPRED-3.1	0.703	0.581	0.116	0.306	0.712	0.588	0.884	646
Predisorder	0.697	0.579	0.116	0.303	0.705	0.582	0.884	642
RawMSA	0.736	0.612	0.182	0.299	0.690	0.668	0.818	646
AUCpreD-np	0.722	0.601	0.155	0.297	0.679	0.637	0.845	646
SPOT-Disorder-Single	0.730	0.608	0.172	0.290	0.679	0.651	0.828	646
MobiDB-lite	0.673	0.550	0.120	0.274	0.692	0.542	0.880	645
IsUnstruct	0.691	0.574	0.157	0.272	0.674	0.591	0.843	646
ESpritz-X	0.712	0.586	0.195	0.272	0.646	0.646	0.805	645
IUPred2A-short	0.693	0.567	0.151	0.270	0.666	0.591	0.849	646
IUPred-short	0.700	0.574	0.162	0.270	0.660	0.607	0.838	645
ESpritz-N	0.661	0.524	0.103	0.268	0.678	0.517	0.897	645
VSL2B	0.674	0.549	0.144	0.264	0.676	0.556	0.856	644
JRONN	0.657	0.528	0.117	0.258	0.677	0.518	0.883	645
fIDPnn	0.713	0.591	0.253	0.252	0.654	0.673	0.747	645
DynaMine	0.657	0.527	0.139	0.245	0.643	0.533	0.861	645
IUPred-long	0.679	0.540	0.164	0.244	0.675	0.564	0.836	645
**Gene3D**	0.740	0.625	0.380	0.243	0.657	0.778	0.620	652
IUPred2A-long	0.669	0.529	0.151	0.242	0.677	0.544	0.849	646
fIDPlr	0.689	0.562	0.277	0.230	0.657	0.649	0.723	645
PyHCA	0.642	0.500	0.154	0.226	0.646	0.518	0.846	646
S2D-1	0.603	0.456	0.124	0.218	0.642	0.447	0.876	644
DisEMBL-465	0.610	0.485	0.151	0.209	0.620	0.476	0.849	644
DisoMine	0.693	0.558	0.323	0.205	0.622	0.675	0.677	646
S2D-2	0.649	0.511	0.278	0.190	0.569	0.606	0.722	644
**PDB Remote**	0.662	0.518	0.345	0.184	0.595	0.646	0.655	530
FoldUnfold	0.665	0.553	0.330	0.176	0.618	0.637	0.670	621
GlobPlot	0.549	0.394	0.127	0.160	0.605	0.365	0.873	645
ESpritz-D	0.670	0.534	0.374	0.152	0.581	0.666	0.626	645
**Conservation**	0.557	0.396	0.261	0.135	0.565	0.464	0.739	652
DisEMBL-HL	0.661	0.531	0.523	0.132	0.514	0.763	0.477	644
DisPredict-2	0.580	0.435	0.401	0.061	0.519	0.543	0.599	646
DFLpred	0.368	0.046	0.009	0.024	0.515	0.029	0.991	646

Supplementary Table 9. Per protein classification performance in the *DisProt-PDB* dataset
Performance of predictors and baselines for *DisProt-PDB* dataset. Metrics are averaged over targets (proteins) and sorted by MCC. Predictors thresholds are optimized on MCC. Baselines are shown in bold. COV is coverage, i.e. number of predicted target proteins.

	BAC	F1-S	FPR	MCC	PPV	TPR	TNR	COV
**PDB observed**	0.898	0.886	0.000	0.854	1.000	0.796	1.000	652
SPOT-Disorder2	0.836	0.784	0.055	0.706	0.851	0.727	0.945	610
SPOT-Disorder1	0.846	0.788	0.090	0.696	0.795	0.782	0.910	644
**PDB Close**	0.811	0.755	0.033	0.689	0.891	0.655	0.967	604
AUCpreD	0.816	0.756	0.070	0.662	0.820	0.701	0.930	644
SPOT-Disorder-Single	0.817	0.751	0.095	0.646	0.775	0.729	0.905	646
RawMSA	0.815	0.745	0.106	0.635	0.755	0.736	0.894	646
Predisorder	0.788	0.717	0.067	0.619	0.813	0.642	0.933	642
AUCpreD-np	0.797	0.725	0.092	0.615	0.769	0.686	0.908	646
DISOPRED-3.1	0.796	0.724	0.092	0.613	0.768	0.684	0.908	646
IUPred-long	0.783	0.704	0.096	0.588	0.754	0.661	0.904	645
IsUnstruct	0.779	0.700	0.091	0.585	0.760	0.648	0.909	646
IUPred2A-long	0.776	0.697	0.087	0.584	0.766	0.640	0.913	646
MobiDB-lite	0.764	0.683	0.063	0.583	0.806	0.592	0.937	645
VSL2B	0.774	0.695	0.087	0.581	0.765	0.636	0.913	644
fIDPnn	0.782	0.701	0.113	0.576	0.727	0.676	0.887	645
IUPred2A-short	0.773	0.691	0.094	0.574	0.752	0.640	0.906	646
IUPred-short	0.775	0.693	0.104	0.571	0.738	0.654	0.896	645
ESpritz-X	0.778	0.695	0.119	0.566	0.717	0.675	0.881	645
ESpritz-N	0.751	0.662	0.073	0.554	0.779	0.575	0.927	645
DisoMine	0.780	0.693	0.160	0.550	0.668	0.721	0.840	646
JRONN	0.751	0.661	0.081	0.546	0.762	0.583	0.919	645
ESpritz-D	0.778	0.690	0.166	0.544	0.660	0.723	0.834	645
**Gene3D**	0.785	0.692	0.220	0.539	0.615	0.791	0.780	652
fIDPlr	0.761	0.671	0.119	0.537	0.705	0.641	0.881	645
DynaMine	0.739	0.641	0.110	0.505	0.704	0.588	0.890	645
PyHCA	0.731	0.629	0.107	0.494	0.704	0.569	0.893	646
S2D-1	0.724	0.617	0.089	0.494	0.728	0.536	0.911	644
FoldUnfold	0.736	0.636	0.193	0.462	0.608	0.666	0.807	621
DisEMBL-465	0.694	0.570	0.110	0.426	0.667	0.498	0.890	644
S2D-2	0.703	0.591	0.253	0.386	0.536	0.658	0.747	644
**PDB Remote**	0.703	0.579	0.273	0.377	0.505	0.678	0.727	530
GlobPlot	0.641	0.480	0.111	0.328	0.613	0.394	0.889	645

DisEMBL-HL	0.641	0.535	0.470	0.262	0.415	0.752	0.530	644
DisPredict-2	0.625	0.491	0.285	0.240	0.455	0.534	0.715	646
**Conservation**	0.618	0.485	0.296	0.227	0.445	0.533	0.704	652
DFLpred	0.504	0.027	0.005	0.043	0.530	0.014	0.995	646

Supplementary Table 10. Per-residue classification performance in the *DisProt-PDB* dataset
Performance of predictors and baselines for *DisProt-PDB* dataset. Metrics are calculated over the whole dataset and sorted by MCC. Predictors thresholds are optimized on MCC. Baselines are shown in bold. COV is coverage, i.e. number of predicted target proteins.

Disprot-Binding dataset

Evaluation Results

	BAC	F1-S	FPR	MCC	PPV	TPR	TNR	COV
DisoRDPbind-protein	0.652	0.137	0.357	0.062	0.139	0.229	0.643	646
ANCHOR-2	0.677	0.130	0.319	0.055	0.138	0.213	0.681	646
**Gene3D**	0.529	0.143	0.523	0.053	0.125	0.261	0.477	652
MoRFchibi-light	0.629	0.124	0.339	0.041	0.125	0.178	0.661	644
MoRFchibi-web	0.588	0.131	0.407	0.039	0.122	0.205	0.593	644
OPAL	0.482	0.141	0.583	0.039	0.116	0.272	0.417	644
DISOPRED-3.1-binding	0.725	0.095	0.172	0.036	0.130	0.105	0.828	646
ANCHOR	0.571	0.127	0.462	0.026	0.112	0.231	0.538	645
fMoRFpred	0.790	0.031	0.033	0.014	0.128	0.020	0.967	646
DisoRDPbind-DNA	0.804	0.005	0.005	0.004	0.122	0.003	0.995	646
DisoRDPbind-RNA	0.799	0.007	0.011	0.002	0.098	0.005	0.989	646
DisoRDPbind	0.194	0.131	1.000	0.000	0.100	0.358	0.000	646
**PDB observed**	0.490	0.128	0.569	-0.011	0.115	0.224	0.431	652

Supplementary Table 11. Per-protein classification performance in the *DisProt-Binding* dataset
Performance of predictors and baselines for *DisProt-Binding* dataset. Metrics are averaged over targets (proteins) and sorted by MCC. Predictors thresholds are optimized on MCC. Baselines are shown in bold. COV is coverage, i.e. number of predicted target proteins.

	BAC	F1-S	FPR	MCC	PPV	TPR	TNR	COV
ANCHOR-2	0.694	0.220	0.320	0.199	0.130	0.708	0.680	646
DisoRDPbind-protein	0.697	0.214	0.353	0.198	0.125	0.746	0.647	646
MoRFchibi-light	0.636	0.212	0.200	0.161	0.137	0.472	0.800	644
**Gene3D**	0.656	0.175	0.516	0.153	0.098	0.828	0.484	652
OPAL	0.652	0.186	0.374	0.151	0.108	0.678	0.626	644

ANCHOR	0.651	0.178	0.451	0.148	0.101	0.754	0.549	645
MoRFchibi-web	0.631	0.194	0.257	0.143	0.119	0.519	0.743	644
**PDB observed**	0.606	0.152	0.589	0.106	0.084	0.801	0.411	652
DISOPRED-3.1-binding	0.569	0.169	0.125	0.099	0.124	0.263	0.875	646
fMoRFpred	0.515	0.072	0.017	0.054	0.157	0.047	0.983	646
DisoRDPbind-DNA	0.502	0.008	0.000	0.052	0.724	0.004	1.000	646
DisoRDPbind-RNA	0.501	0.010	0.002	0.014	0.136	0.005	0.998	646
DisoRDPbind	0.500	0.119	1.000	0.000	0.063	1.000	0.000	646

Supplementary Table 12. Per-residue classification performance in the *DisProt-Binding* dataset
Performance of predictors and baselines for *DisProt-Binding* dataset. Metrics are calculated over the whole dataset and sorted by MCC. Predictors thresholds are optimized on MCC. Baselines are shown in bold. COV is coverage, i.e. number of predicted target proteins.

Figures

Dataset

Annotations

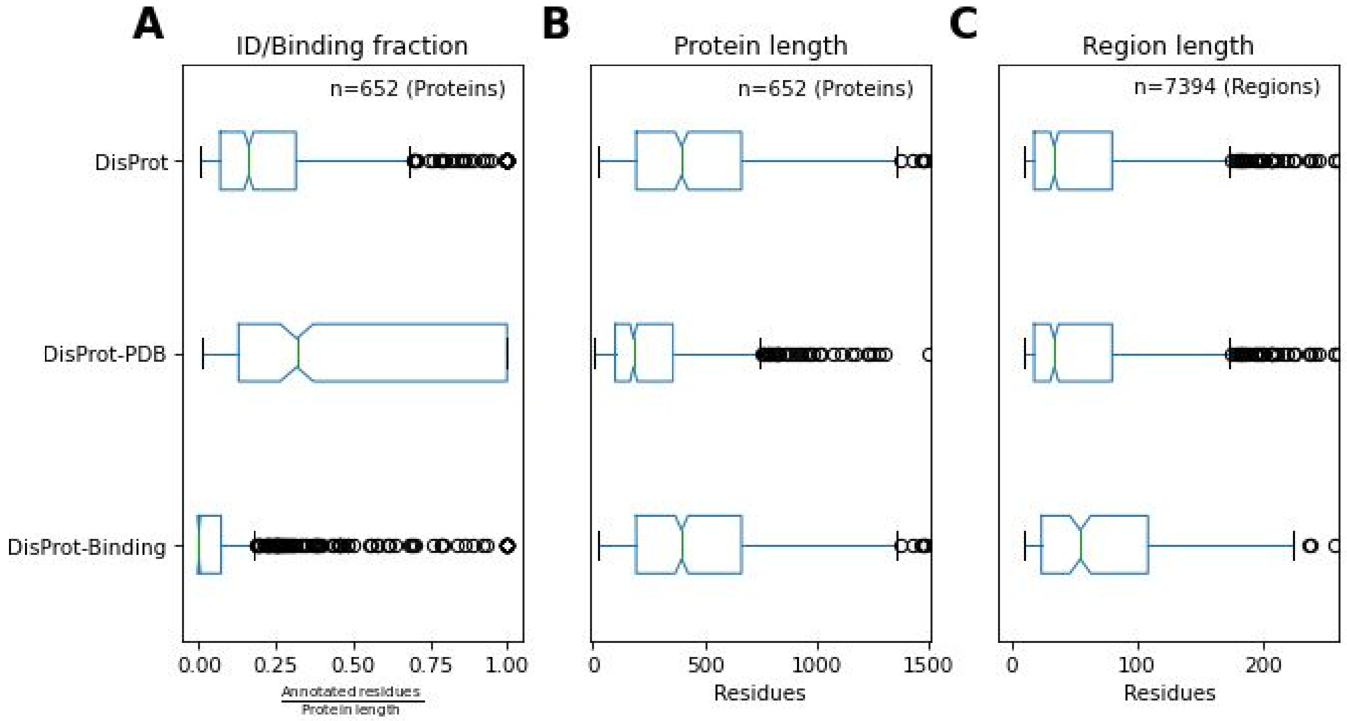

Supplementary Figure 1. Dataset composition.
Distribution of the fraction of disordered / binding residues in each protein (panel A), of the protein lengths (panel B) and region lengths (panel C) in the three datasets (*DisProt*, *DisProt-PDB* and *DisProt-Binding*). Boxplots are defined as follows: the middle value of the dataset is the median (Q2/50th Percentile). The box boundaries are the 1st quartile (Q1/25th Percentile) and 3rd quartile (Q3/75th Percentile) respectively; Maximum is Q3 + 1.5*(Q3-Q1) and Minimum is Q1 -1.5*(Q3-Q1).

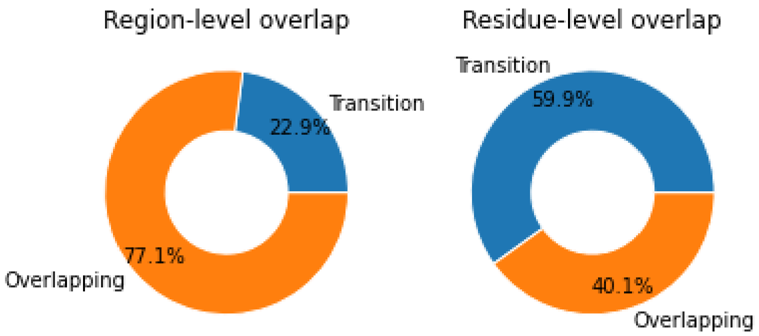

Supplementary Figure 2. Overlap of DisProt structural transitions with PDB.
Fraction of Regions/Residues labelled with a structural transition term from the Disorder Ontology that do (orange) or do not (blue) overlap with PDB resolved regions/residues.

Redundancy

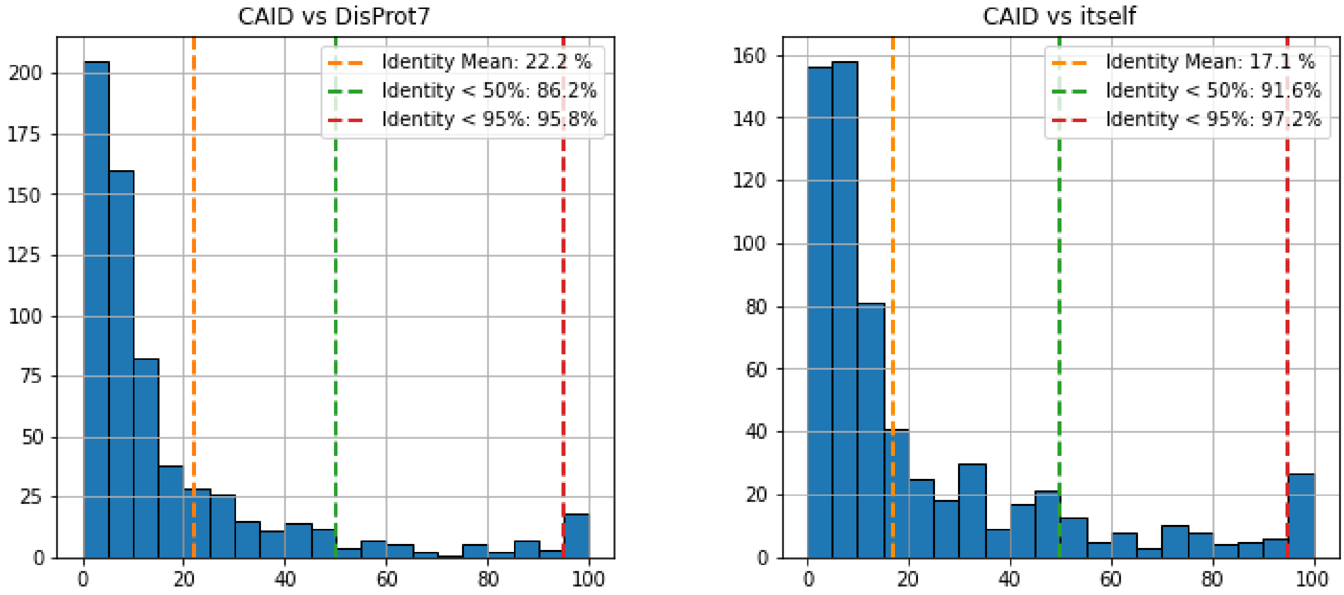

Supplementary Figure 3. Dataset redundancy.
Distribution of highest sequence identity percentage for each target in the CAID dataset when compared with itself (right) and with DisProt 7.0 (left).

Predictor CPU time

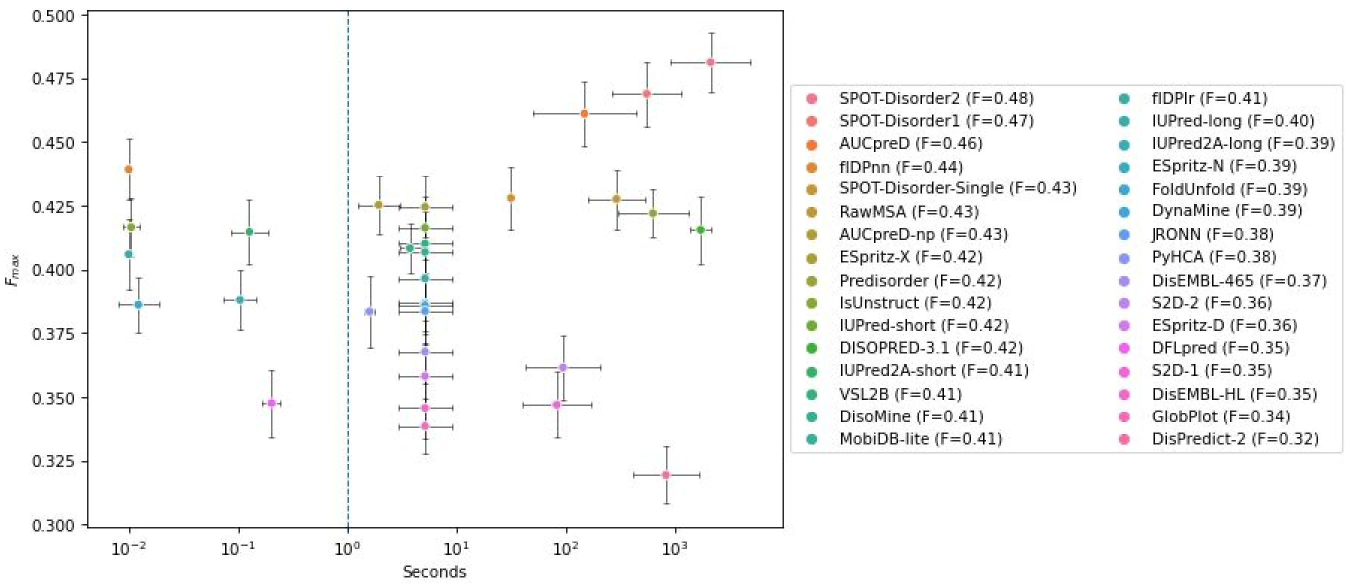

Supplementary Figure 4. CPU time to performance for disorder predictors.
Scatterplot of CPU time in ms in logarithmic scale (x axis) and performance expressed as F_{Max} (y axis) calculated on the *DisProt* dataset. Data is presented as mean +/- SD.

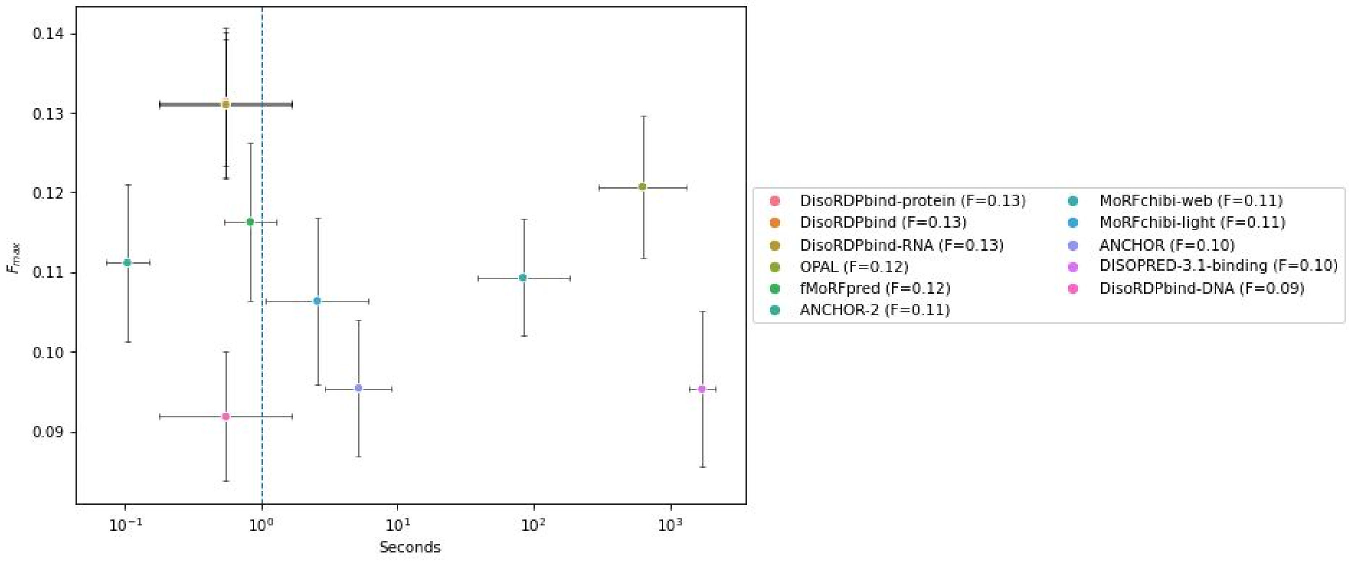

Supplementary Figure 5. CPU time to performance for binding predictors.
Scatterplot of CPU time in ms in logarithmic scale (x axis) and performance expressed as F_{Max} (yaxis) calculated on the *DisProt-Binding* dataset. Data is presented as mean +/- SD.

Species representation

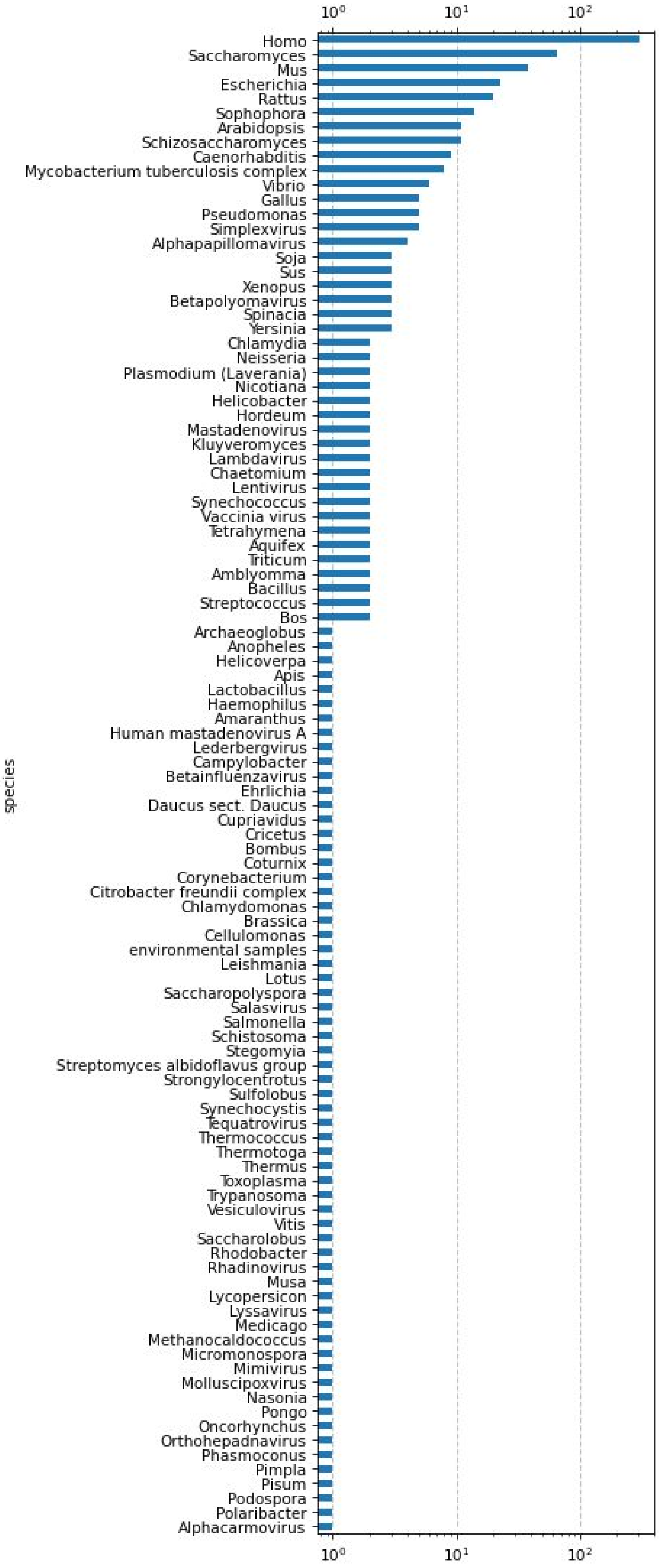

Supplementary Figure 6. Species representation.
Number of entries for each species in the CAID dataset.

Fully ID

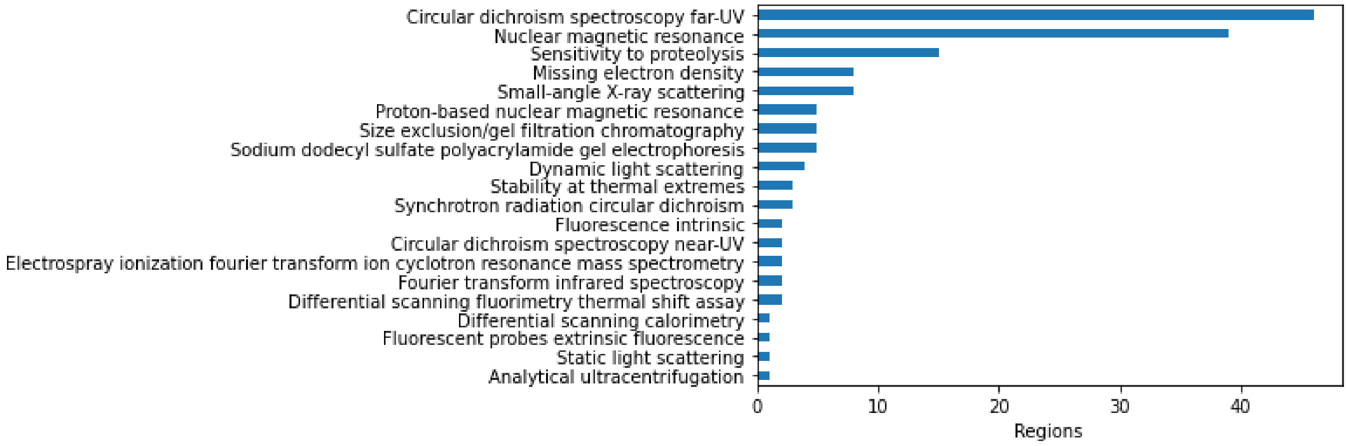

Supplementary Figure 7. Detection method of fully disordered proteins.
Number of regions detected per detection method in fully disordered proteins (calculated as those proteins with a fraction of disorder greater than 95%).

Disorder

DisProt dataset

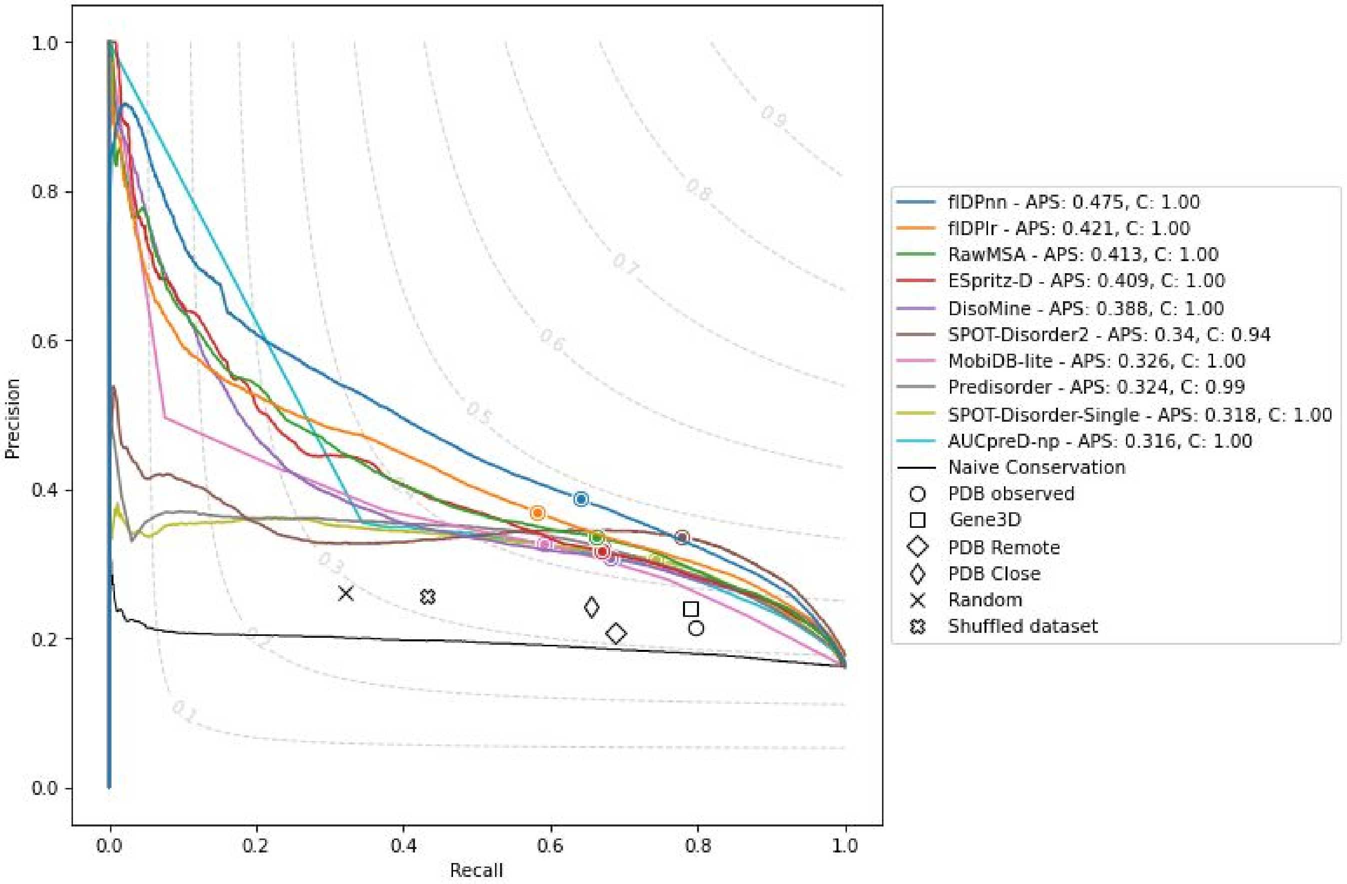

Supplementary Figure 8: Precision recall curve in the *DisProt* dataset.
Precision (y-axis) recall (x-axis) curves of the 10 best ranking methods. Ranking is based on their APS (average precision score) in the *DisProt* dataset.

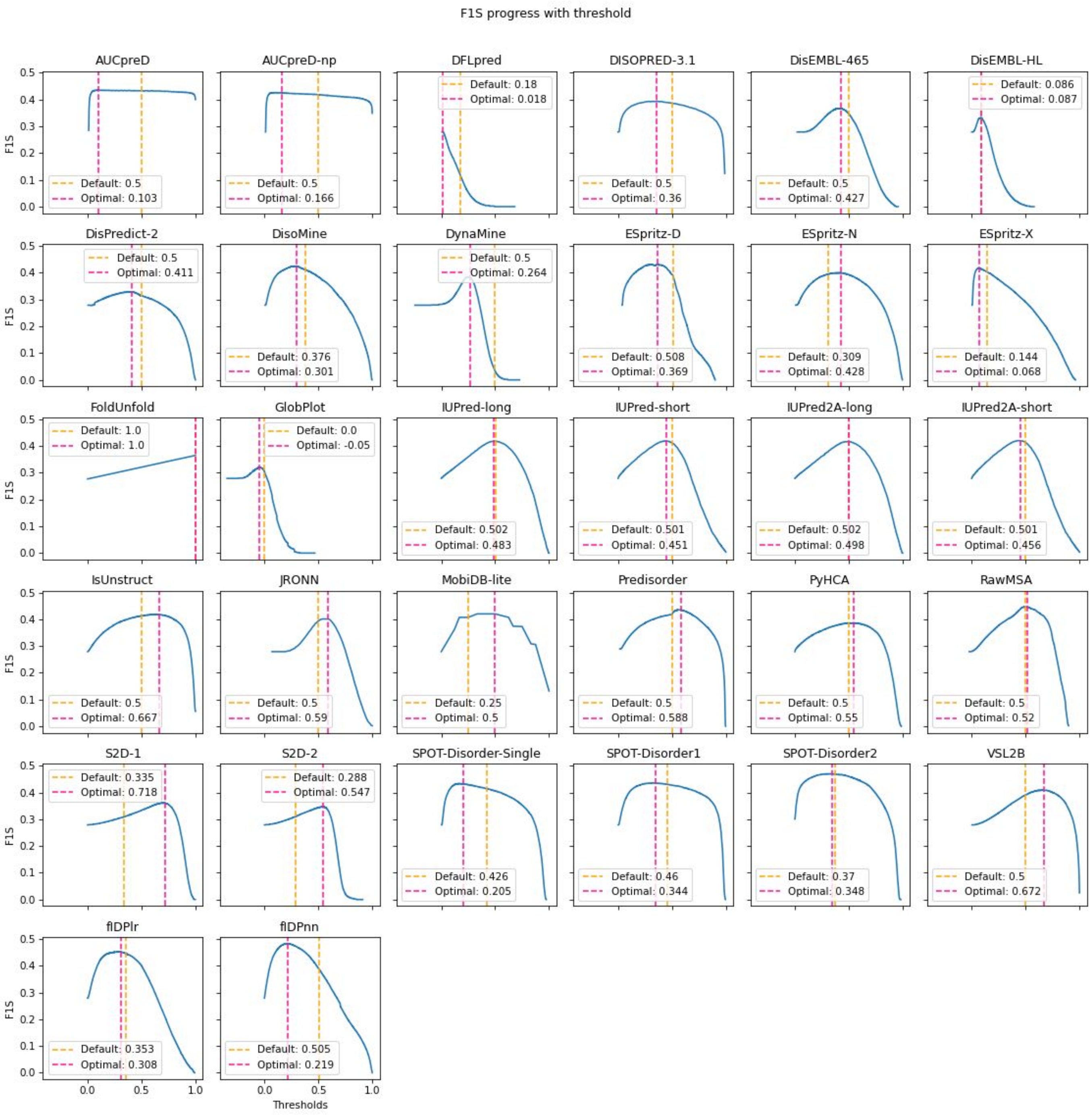

Supplementary Figure 9. F1-Score progress with threshold in the *DisProt* dataset.
F1-score progress (y-axis) with increasing threshold value (x-axis) for each predictor in the *DisProt* dataset.

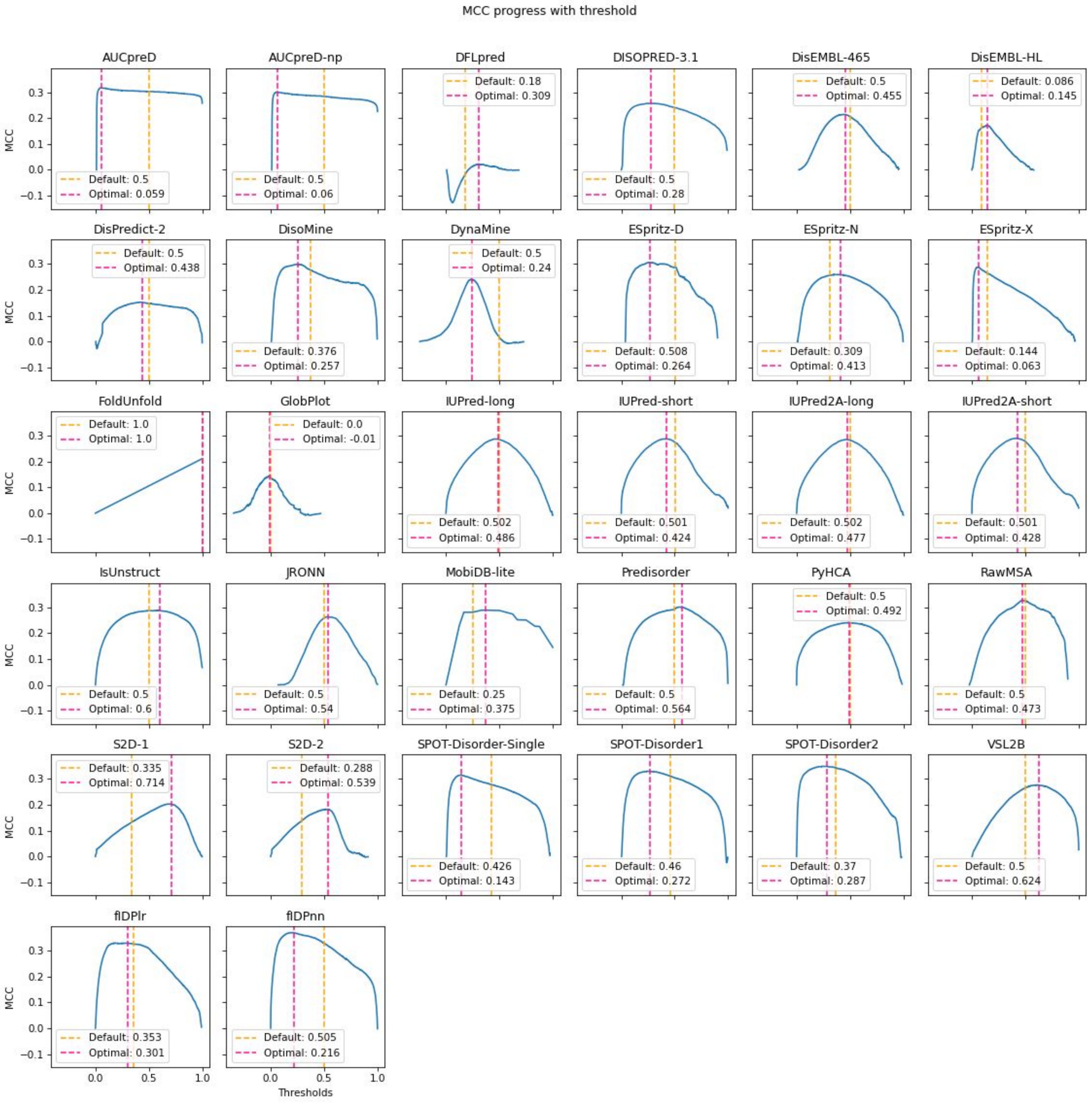

Supplementary Figure 10. MCC progress with threshold in the *DisProt* dataset.

MCC progress (y-axis) with increasing threshold value (x-axis) for each predictor in the *DisProt* dataset.

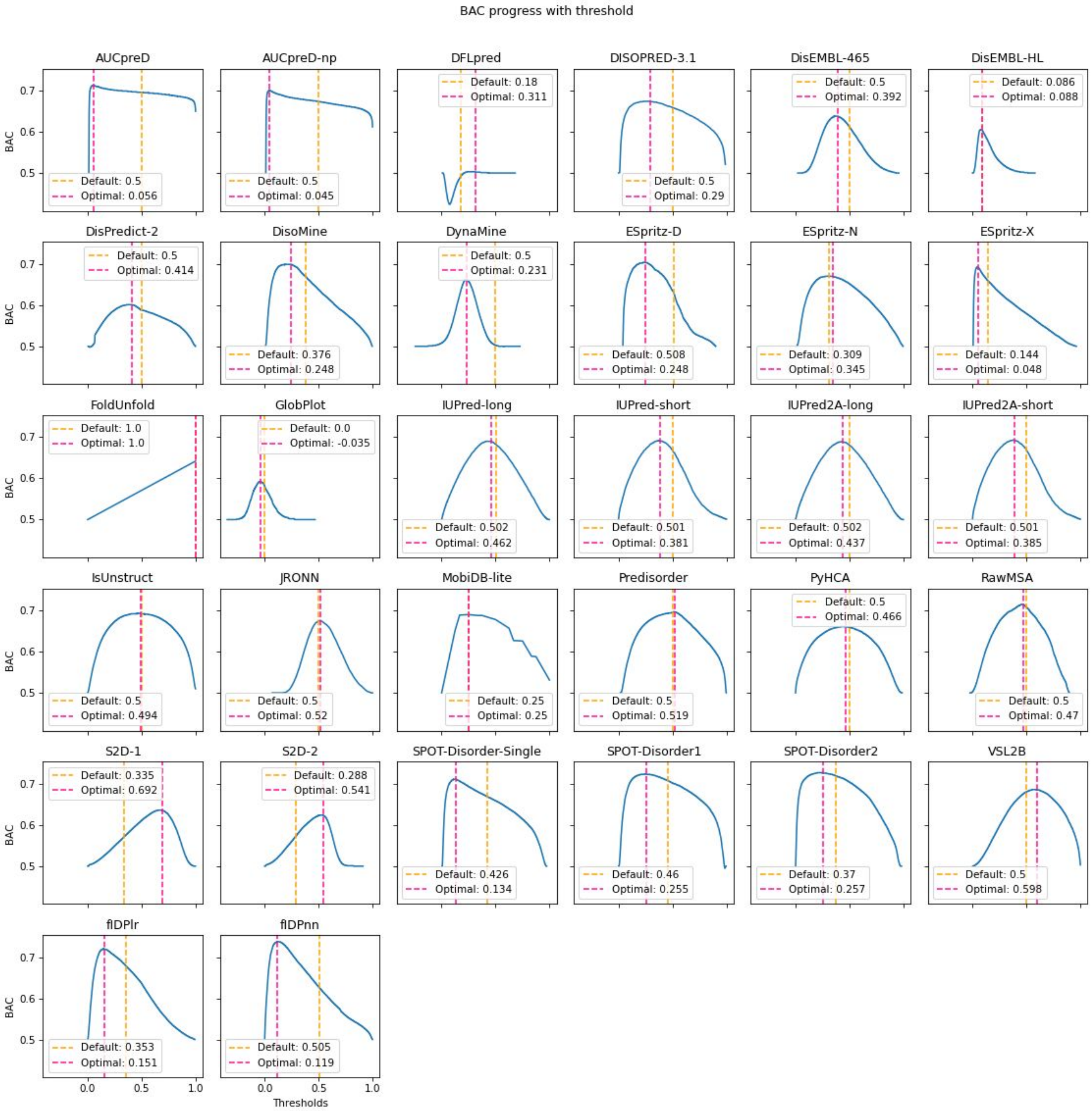

Supplementary Figure 11. Balanced accuracy progress with threshold in the *DisProt* dataset. Balanced accuracy progress (y-axis) with increasing threshold value (x-axis) for each predictor in the *DisProt* dataset.

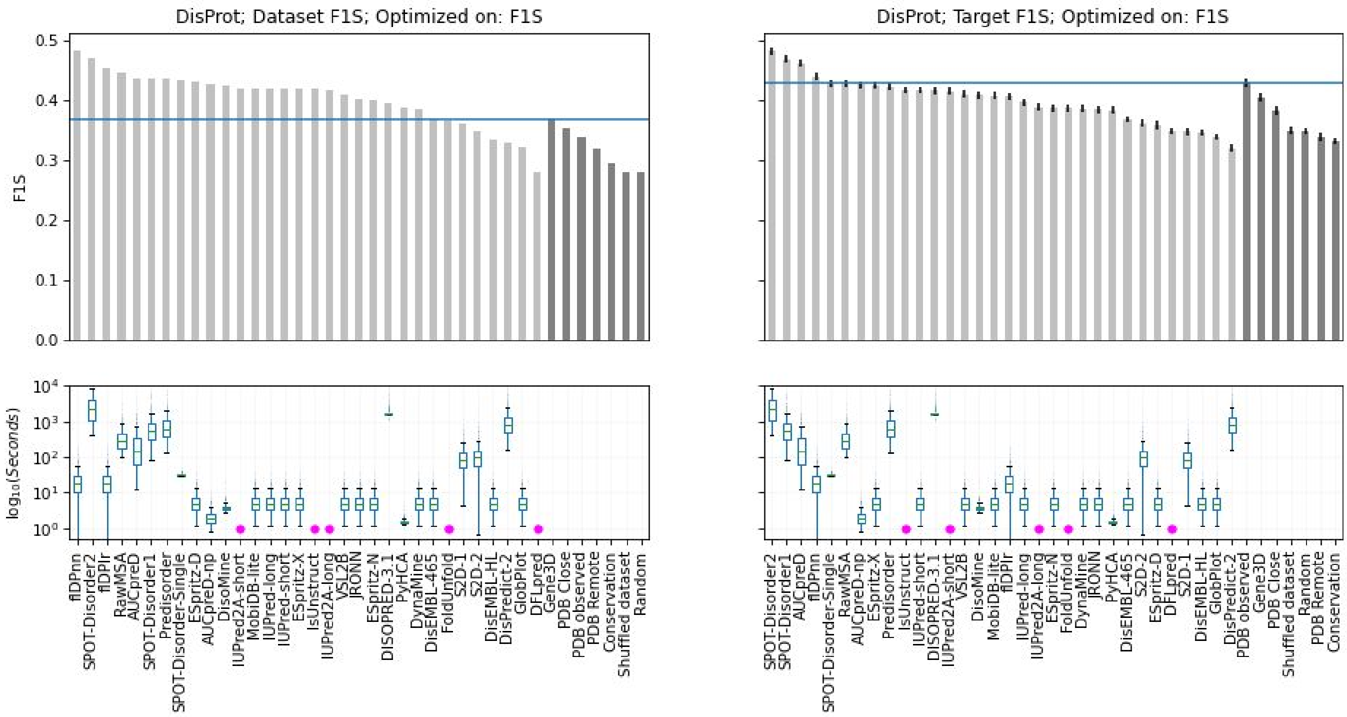

Supplementary Figure 12: F_{Max} in the *DisProt* dataset.
F_{Max} calculated on the whole dataset with confidence intervals as error bars (left) and averaged over proteins with Standard-Error as error bars (right). Calculated on *DisProt* dataset (n= 646 proteins). Boxplots are defined as follows: the middle value of the dataset is the median (Q2/50th Percentile). The box boundaries are the 1st quartile (Q1/25th Percentile) and 3rd quartile (Q3/75th Percentile) respectively; Maximum is Q3 + 1.5*(Q3-Q1) and Minimum is Q1 -1.5*(Q3-Q1). Outliers are hidden for clarity Magenta dots indicate that the whole distribution of execution-times is lower than 1 second.

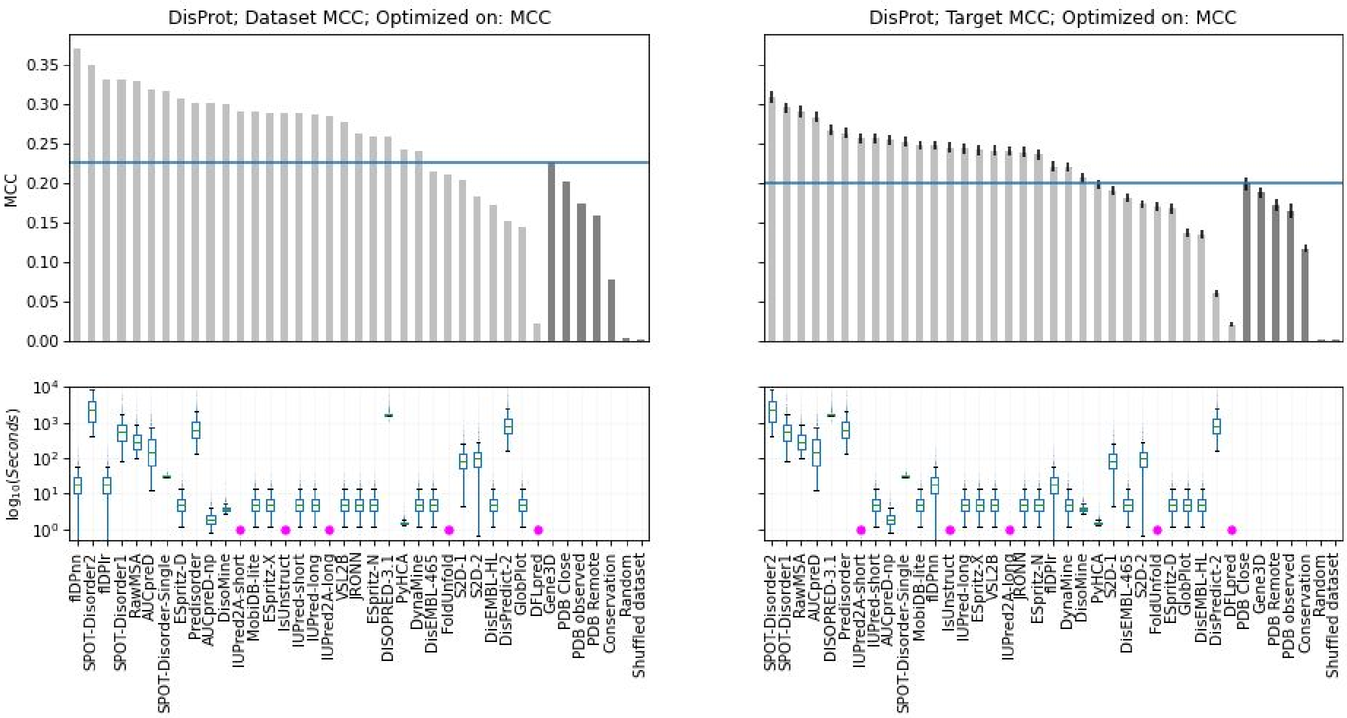

Supplementary Figure 13: MCC in the *DisProt* dataset.
MCC calculated on the whole dataset with confidence intervals as error bars (left) and averaged over proteins with Standard-Error as error bars (right). Calculated on *DisProt* dataset (n= 646 proteins). Predictors threshold is optimized on MCC. Boxplots are defined as follows: the middle value of the

dataset is the median (Q2/50th Percentile). The box boundaries are the 1st quartile (Q1/25th Percentile) and 3rd quartile (Q3/75th Percentile) respectively; Maximum is Q3 + 1.5*(Q3-Q1) and Minimum is Q1 -1.5*(Q3-Q1). Outliers are hidden for clarity. Magenta dots indicate that the whole distribution of execution-times is lower than 1 second.

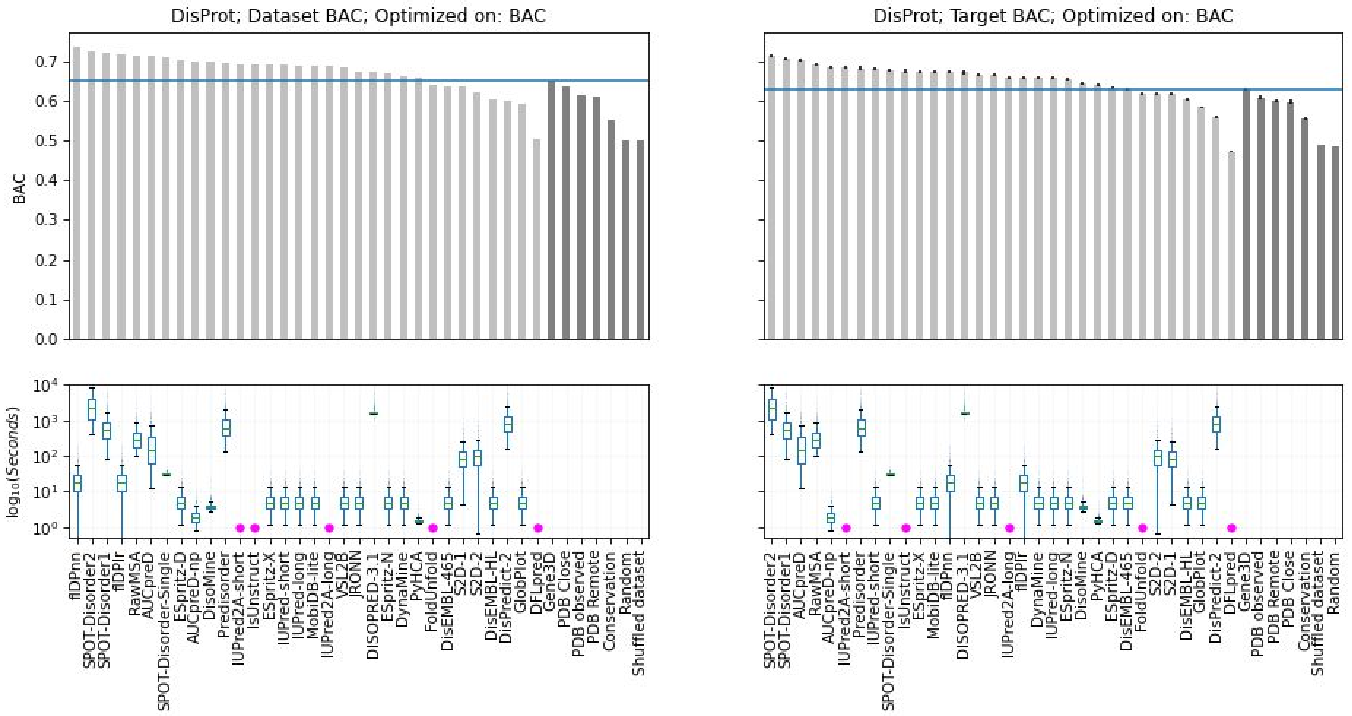

Supplementary Figure 14: Balanced accuracy in the *DisProt* dataset.
Balanced accuracy calculated on the whole dataset with confidence intervals as error bars (left) and averaged over proteins with Standard-Error as error bar (right). Calculated *DisProt* dataset (n= 646 proteins). Predictors threshold is optimized on Balanced accuracy. Boxplots are defined as follows: the middle value of the dataset is the median (Q2/50th Percentile). The box boundaries are the 1st quartile (Q1/25th Percentile) and 3rd quartile (Q3/75th Percentile) respectively; Maximum is Q3 + 1.5*(Q3-Q1) and Minimum is Q1 -1.5*(Q3-Q1). Outliers are hidden for clarity. Magenta dots indicate that the whole distribution of execution-times is lower than 1 second.

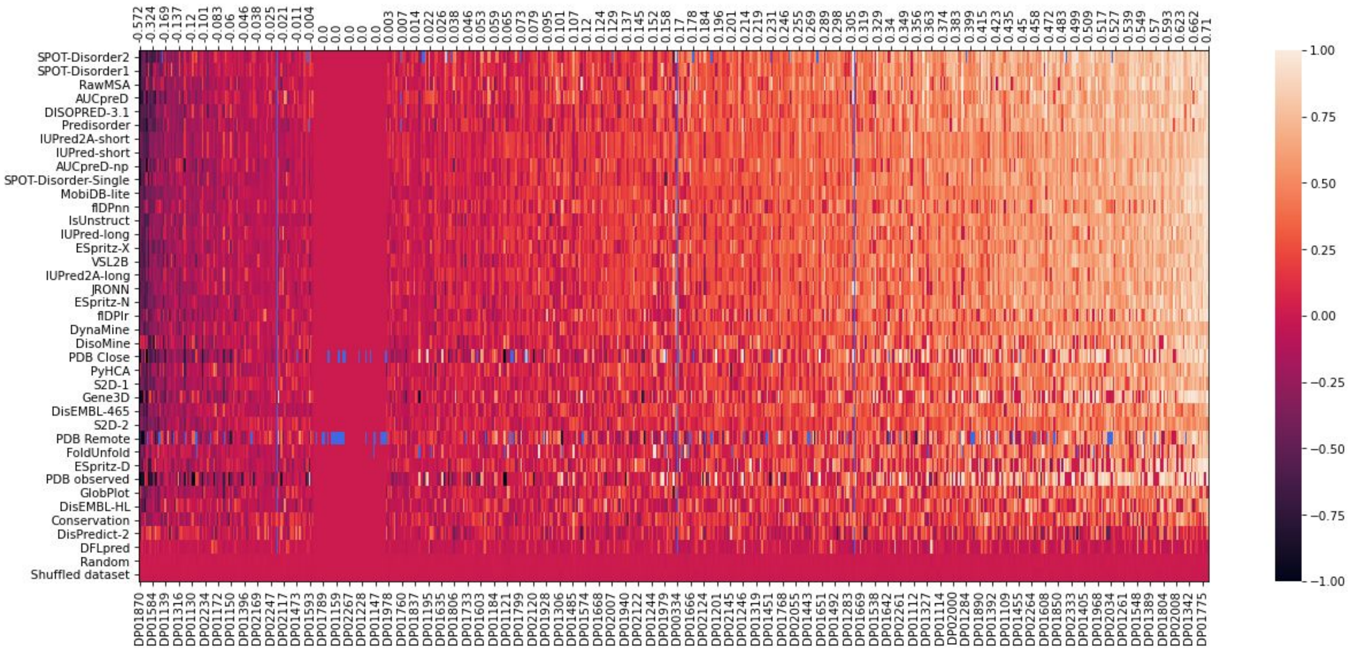

Supplementary Figure 15: MCC per protein in the *DisProt* dataset.

MCC of each target (x-axis, bottom labels, not all labels are visible) from each predictor (y-axis). Targets are sorted by average MCC (x-axis, top labels). Calculated on *DisProt* dataset. Predictors threshold is optimized on MCC. Missing values are in blue.

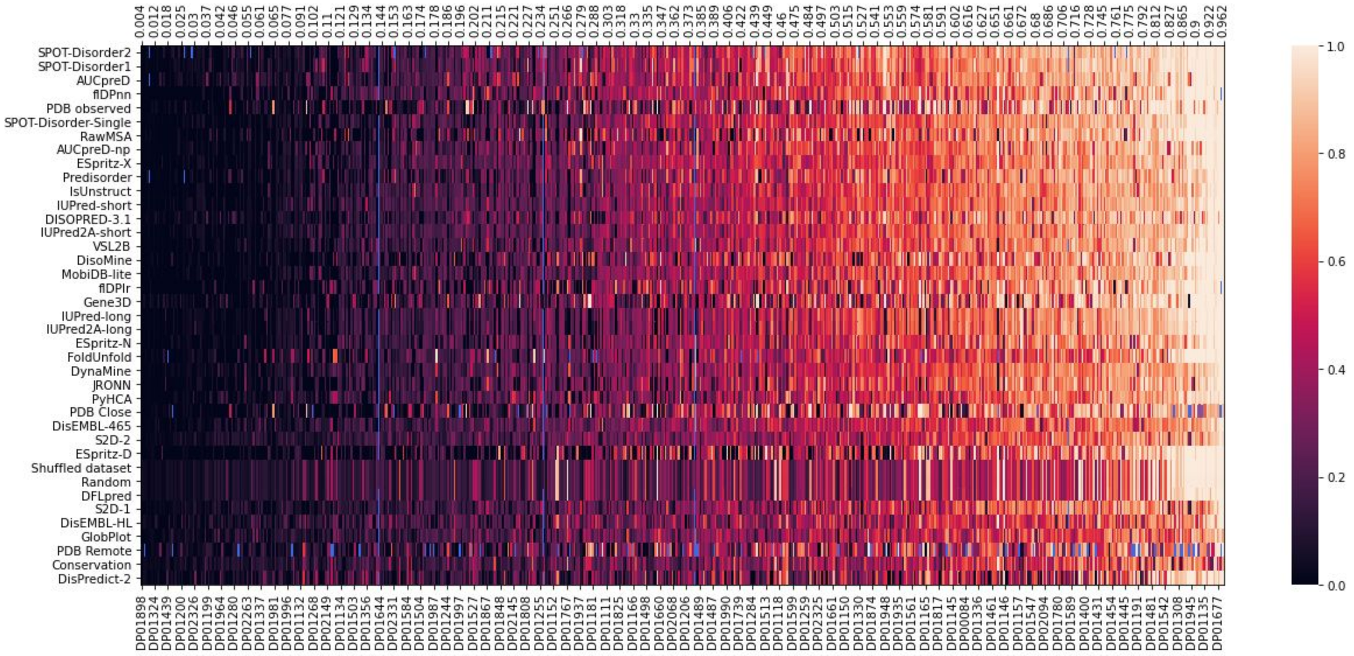

Supplementary Figure 16: F_{Max} per protein in the *DisProt* dataset.

F_{Max} of each target (x-axis, bottom labels, not all labels are visible) from each predictor (y-axis). Targets are sorted by average F_{Max} (x-axis, top labels). Calculated on all *DisProt* dataset. Missing values are in blue.

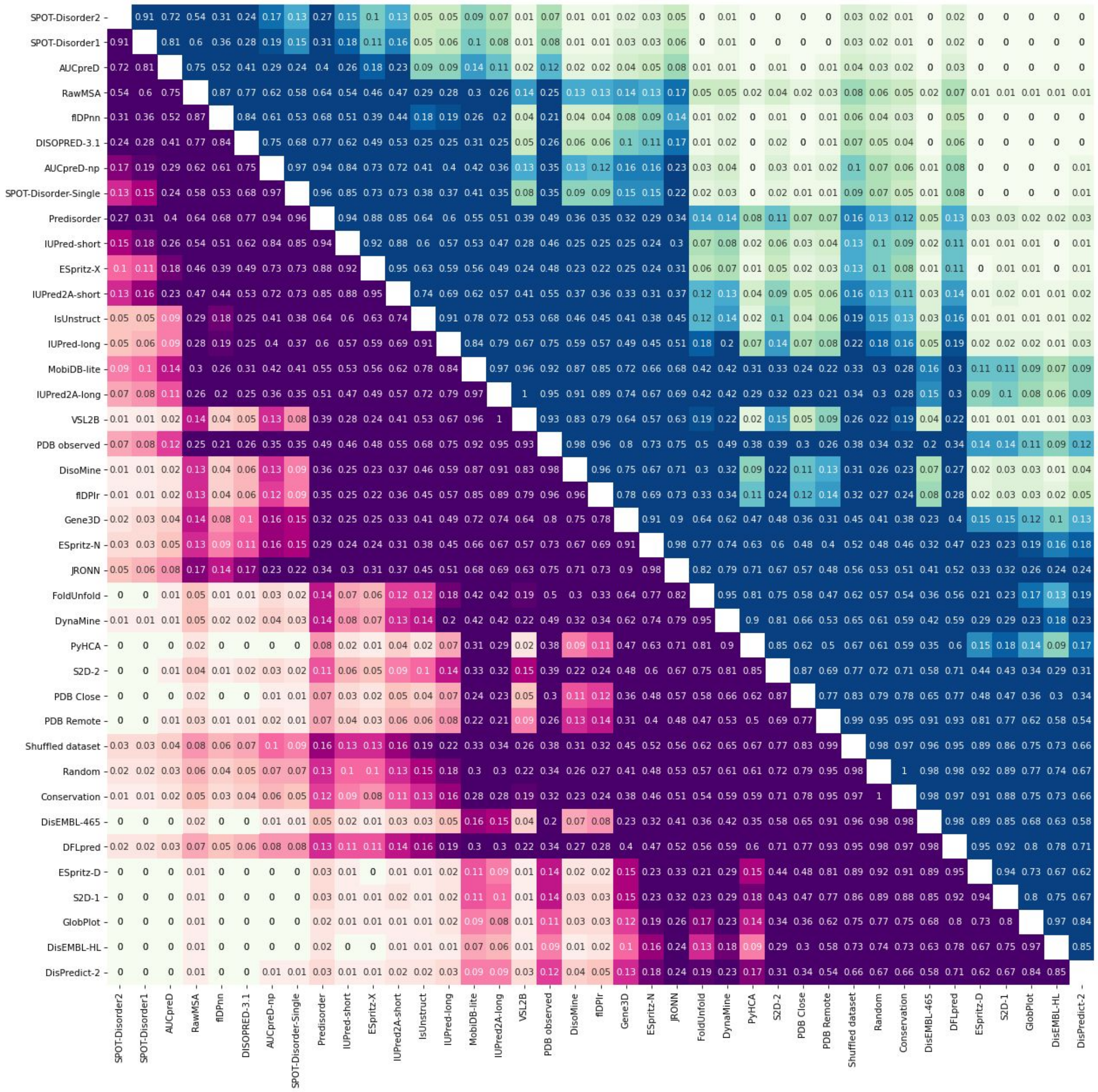

Supplementary Figure 17. Overall average ranking of all predictors and baselines in the *DisProt* dataset.

Heatmap of the T-test p-value associated to the statistical significance of the difference between ranking distribution of predictors. A ranking distribution for a predictor is the position of that predictor in its ranking for the following metrics: 'bac', 'f1s', 'fpr', 'mcc', 'ppv', 'tpr', 'tnr'. Metrics used are: bac, f1s, fpr, mcc, ppv, tpr, tnr; they are calculated per target and with predictors threshold optimized by F1-Score.

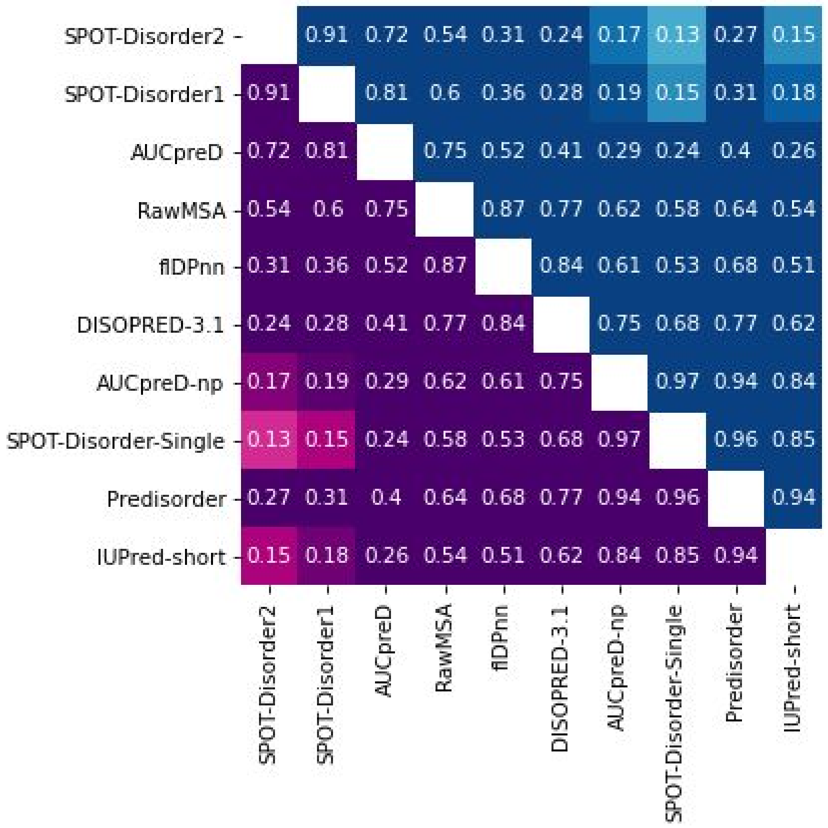

Supplementary Figure 18. Overall average ranking of the 10 best ranking predictors and baselines in the *DisProt* dataset.

Heatmap of the T-test p-value associated to the statistical significance of the difference between ranking distribution of predictors. A ranking distribution for a predictor is the position of that predictor in its ranking for each metric. Metrics used are: bac, f1s, fpr, mcc, ppv, tpr, tnr; they are calculated with predictors threshold optimized by F1-Score.

Mammals

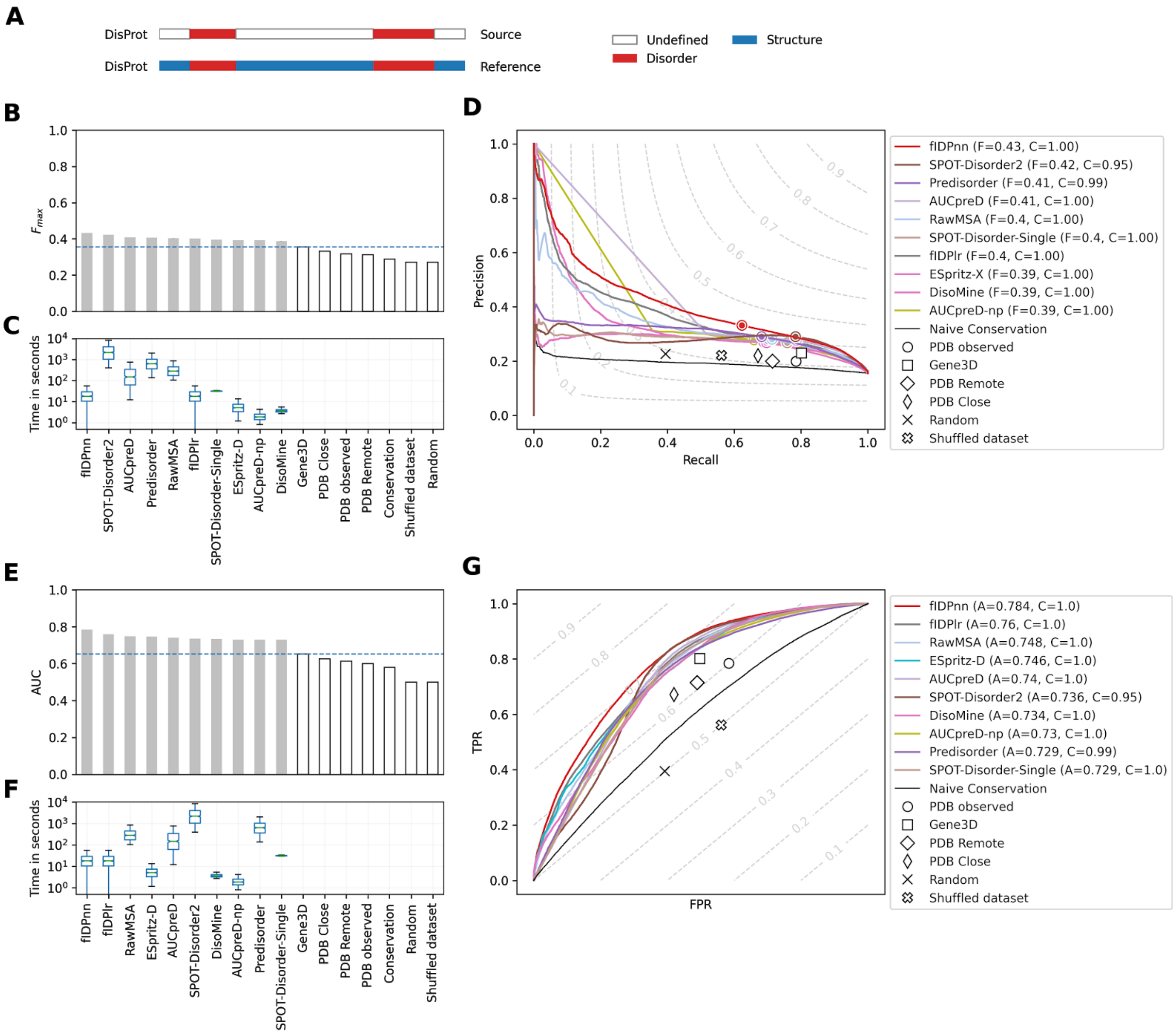

Supplementary Figure 19. Prediction success and CPU times for the ten top-ranking disorder predictors for mammalian proteins in the *DisProt* dataset.

Prediction success and CPU times for the ten top-ranking disorder predictors for mammalian proteins in the *DisProt* dataset (n= 368 proteins). Reference used (*DisProt*) in the analysis and how it is obtained (panel A). Performance of predictors expressed as maximum F1-Score across all thresholds (F_{max}) (panel B) and AUC (panel E) for the top ten best ranking methods (light gray) and baselines (white) and the distribution of execution time per-target (panels C, F) using *DisProt* dataset. The horizontal line in panels B, E indicates the F_{max} and AUC of the best baseline, respectively. Precision-Recall (panel D) and ROC curves (panel G) of ten top-ranking methods and baselines using *DisProt* dataset, with level curves of the F1-Score and Balanced accuracy, respectively. Boxplots in panels **C, F** are defined as follows: the middle value of the dataset is the median (Q2/50th Percentile). The box boundaries are the 1st quartile (Q1/25th Percentile) and 3rd quartile (Q3/75th Percentile) respectively; Maximum is Q3 + 1.5*(Q3-Q1) and Minimum is Q1 -1.5*(Q3-Q1). Outliers are hidden for clarity. Magenta dots on panels C, F indicate that the whole distribution of execution-times is lower than 1 second.

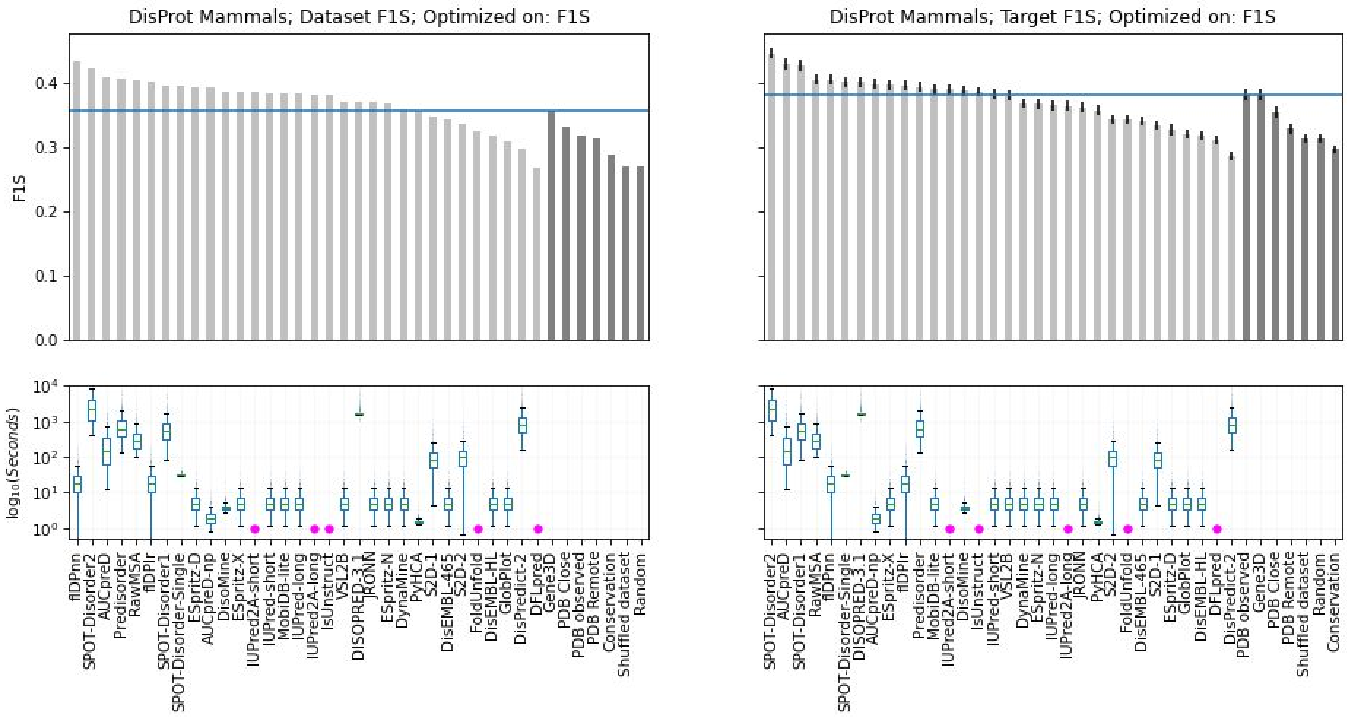

Supplementary Figure 20: F_{Max} for mammalian proteins in *DisProt* dataset.

F_{Max} calculated on the whole dataset with confidence intervals as error bars (left) and averaged over proteins with Standard-Error as error bars (right). Calculated on mammalian proteins of the *DisProt* dataset (n= 368 proteins). Boxplots are defined as follows: the middle value of the dataset is the median (Q2/50th Percentile). The box boundaries are the 1st quartile (Q1/25th Percentile) and 3rd quartile (Q3/75th Percentile) respectively; Maximum is Q3 + 1.5*(Q3-Q1) and Minimum is Q1 -1.5*(Q3-Q1). Outliers are hidden for clarity. Magenta dots indicate that the whole distribution of execution-times is lower than 1 second.

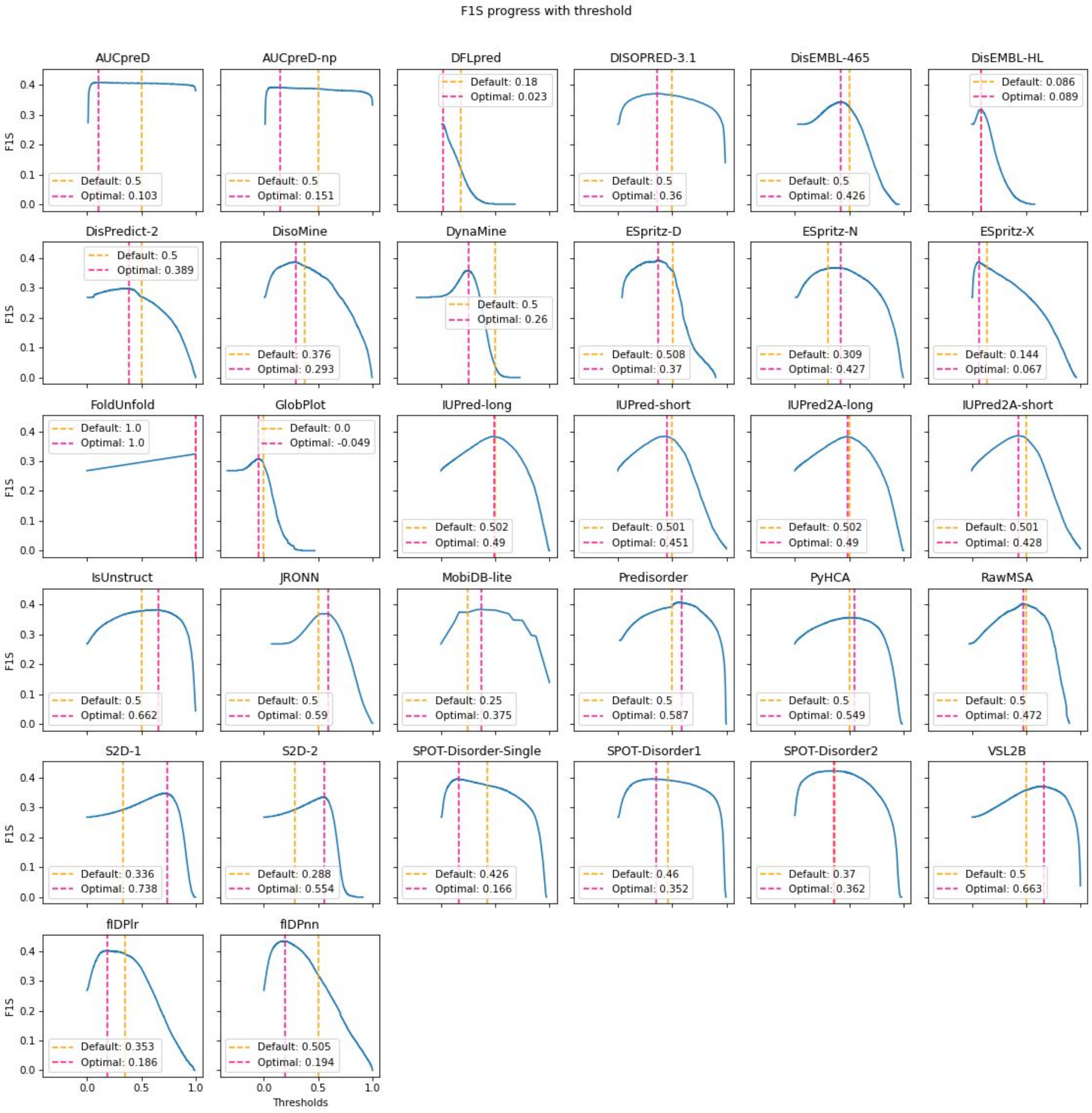

Supplementary Figure 21. F1-Score progress with threshold for mammalian proteins in the *DisProt* dataset.

F1-score progress (y-axis) with increasing threshold value (x-axis) for each predictor calculated on mammalian proteins on the *DisProt* dataset.

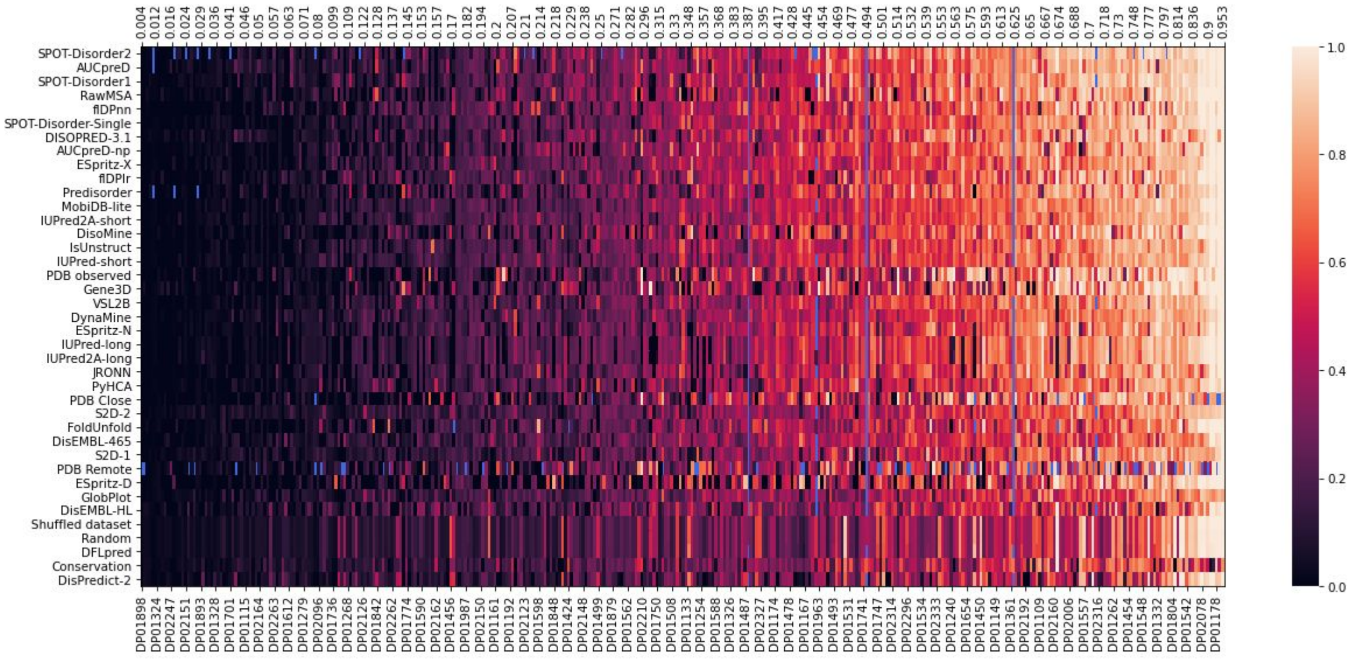

Supplementary Figure 22: F_{Max} per target for mammalian proteins in the *DisProt* dataset.
F_{Max} of each target (x-axis, bottom labels, not all labels are visible) from each predictor (y-axis). Targets are sorted by average F_{Max} (x-axis, top labels). Calculated on mammalian proteins of the *DisProt* dataset. Missing values are in blue.

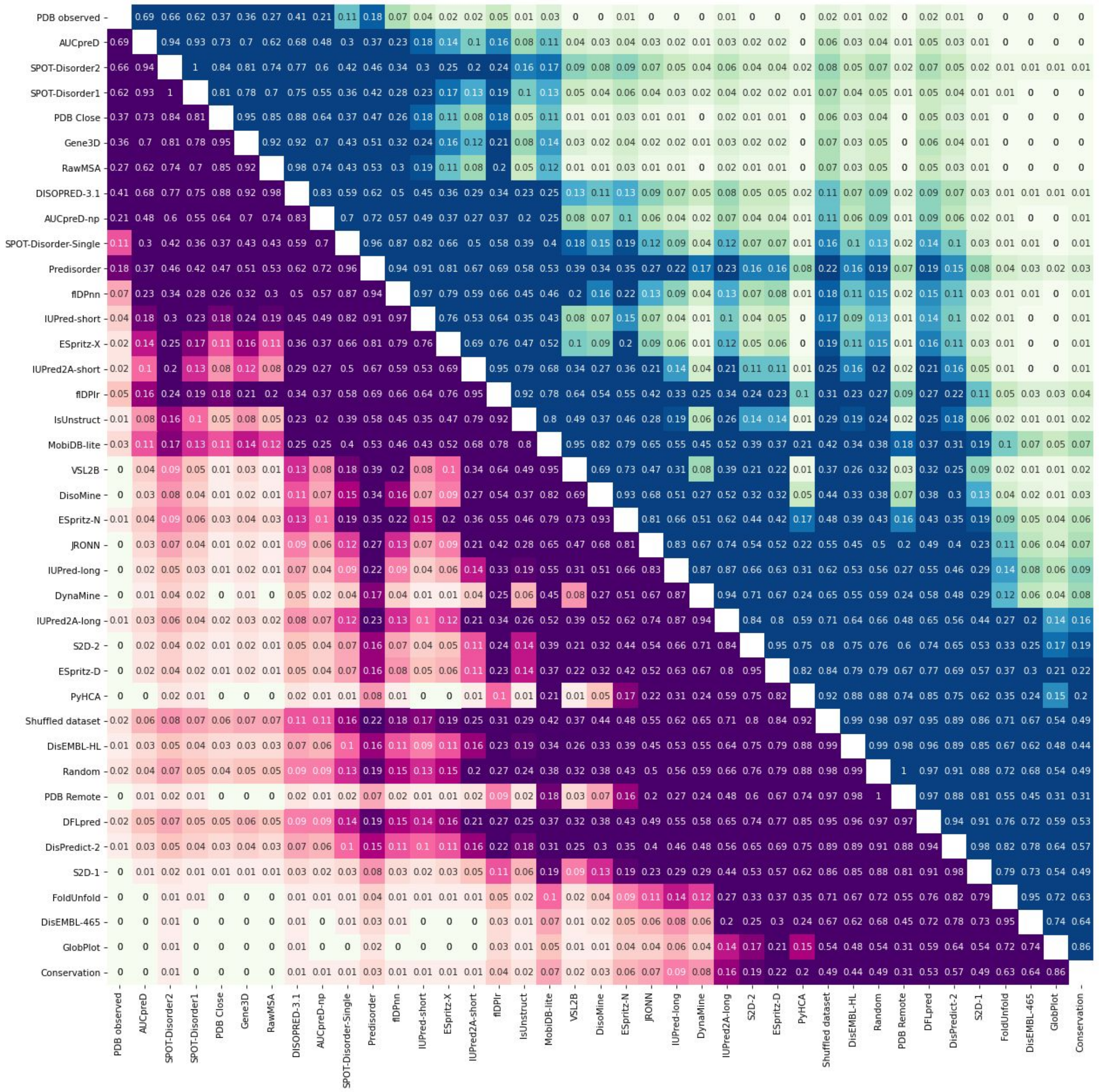

Supplementary Figure 23. Overall average ranking of all predictors and baselines for mammalian proteins in the *DisProt* dataset.

Heatmap of the T-test p-value associated to the statistical significance of the difference between ranking distribution of predictors. A ranking distribution for a predictor is the position of that predictor in its ranking for the following metrics: 'bac', 'f1s', 'fpr', 'mcc', 'ppv', 'tpr', 'tnr'. Metrics are calculated per target and with predictors threshold optimized by F1-Score.

Prokaryotes

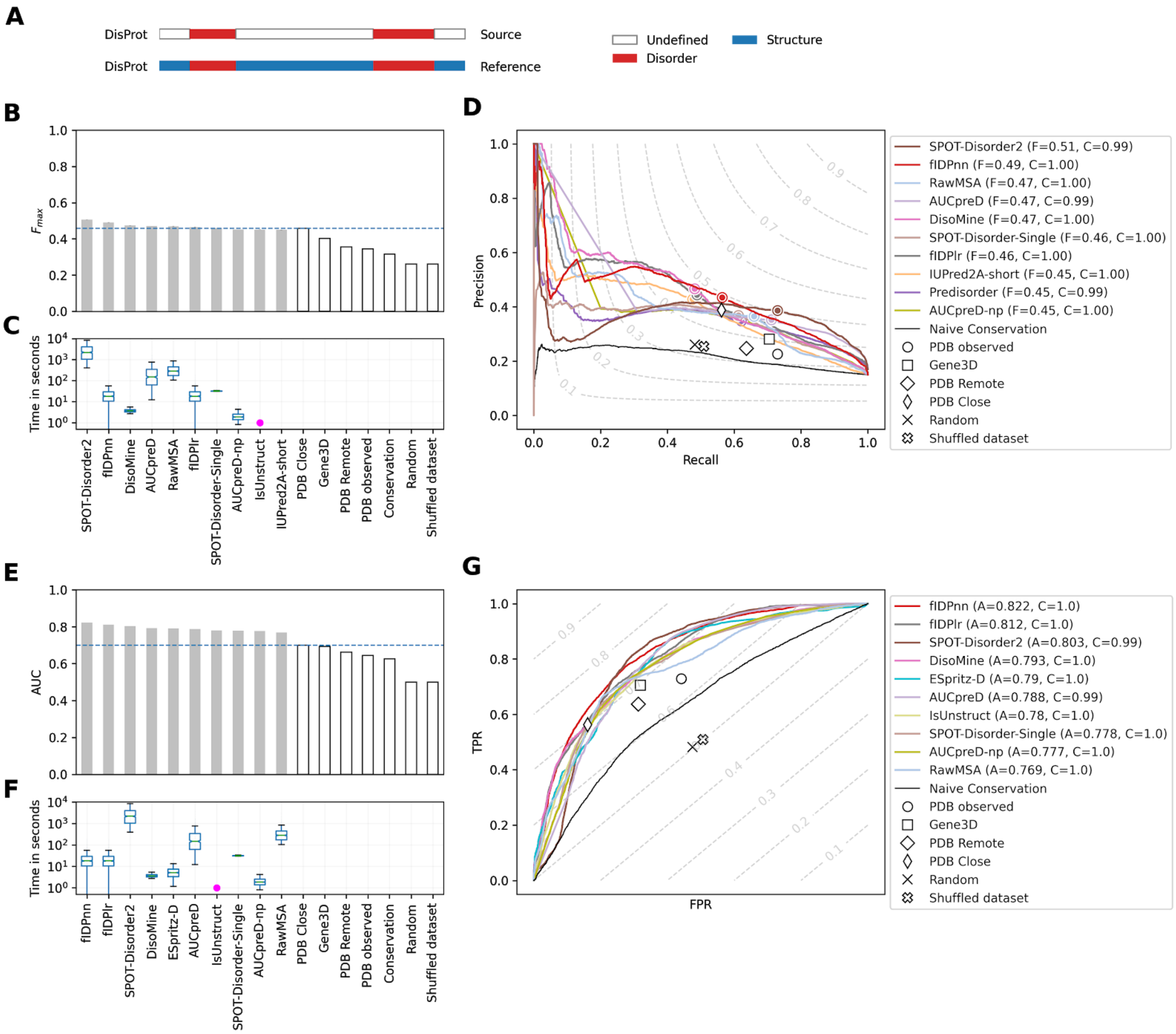

Supplementary Figure 24. Prediction success and CPU times for the ten top-ranking disorder predictors for prokaryotic proteins in the *DisProt* dataset.
Prediction success and CPU times for the ten top-ranking disorder predictors for prokaryotic proteins in the *DisProt* dataset (n= 77 proteins). Reference used (*DisProt*) in the analysis and how it is obtained (panel A). Performance of predictors expressed as maximum F1-Score across all thresholds (F_{max}) (panel B) and AUC (panel E) for the top ten best ranking methods (light gray) and baselines (white) and the distribution of execution time per-target (panels C, F) using *DisProt* dataset. The horizontal line in panels B, E indicates the F_{max} and AUC of the best baseline, respectively. Precision-Recall (panel D) and ROC curves (panel G) of ten top-ranking methods and baselines using *DisProt* dataset, with level curves of the F1-Score and Balanced accuracy, respectively. Boxplots in panels **C, F** are defined as follows: the middle value of the dataset is the median (Q2/50th Percentile). The box boundaries are the 1st quartile (Q1/25th Percentile) and 3rd quartile (Q3/75th Percentile) respectively; Maximum is Q3 + 1.5*(Q3-Q1) and Minimum is Q1 -1.5*(Q3-Q1). Outliers are hidden for clarity. Magenta dots on panels C, F indicate that the whole distribution of execution-times is lower than 1 second.

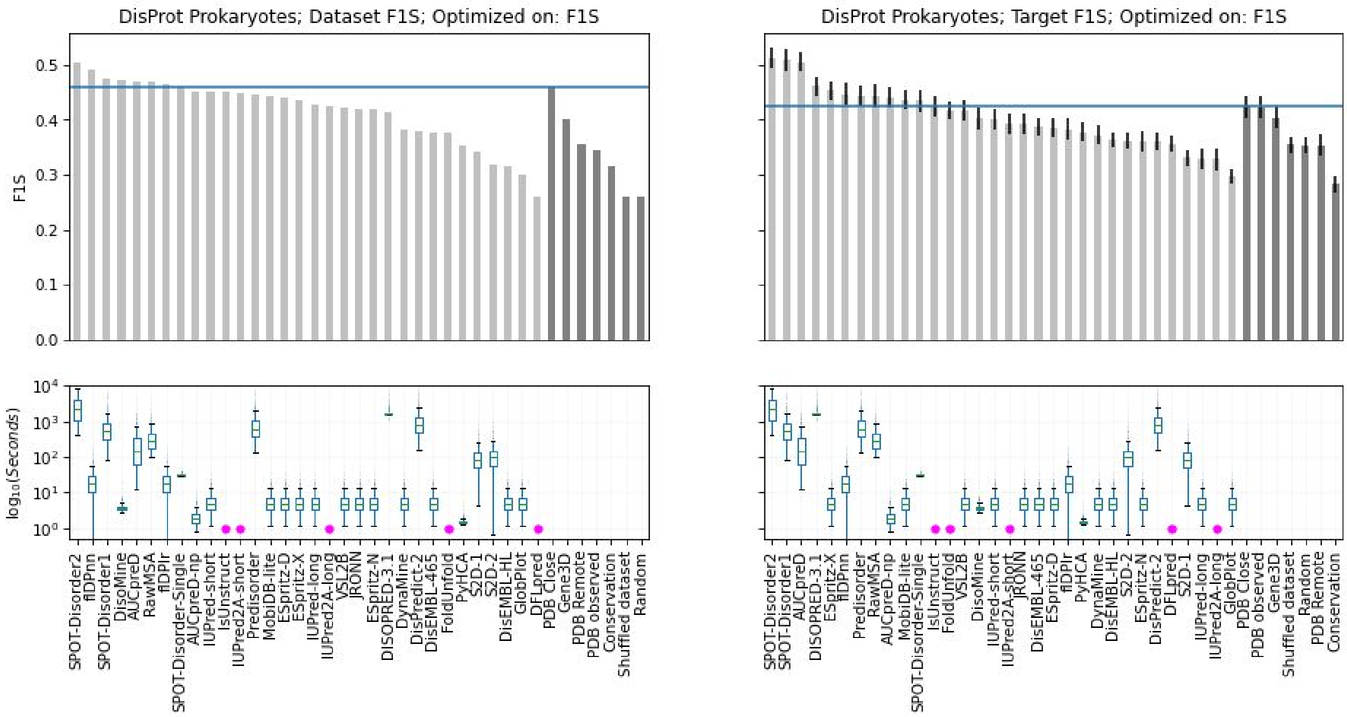

Supplementary Figure 25: F_{Max} for prokaryotic proteins in the *DisProt* dataset.

F_{Max} calculated on the whole dataset with confidence intervals as error bars (left) and averaged over proteins with Standard-Error as error bars (right). Calculated on prokaryotic proteins of the *DisProt* dataset (n= 77 proteins). Boxplots are defined as follows: the middle value of the dataset is the median (Q2/50th Percentile). The box boundaries are the 1st quartile (Q1/25th Percentile) and 3rd quartile (Q3/75th Percentile) respectively; Maximum is Q3 + 1.5*(Q3-Q1) and Minimum is Q1 -1.5*(Q3-Q1). Outliers are hidden for clarity. Magenta dots indicate that the whole distribution of execution-times is lower than 1 second.

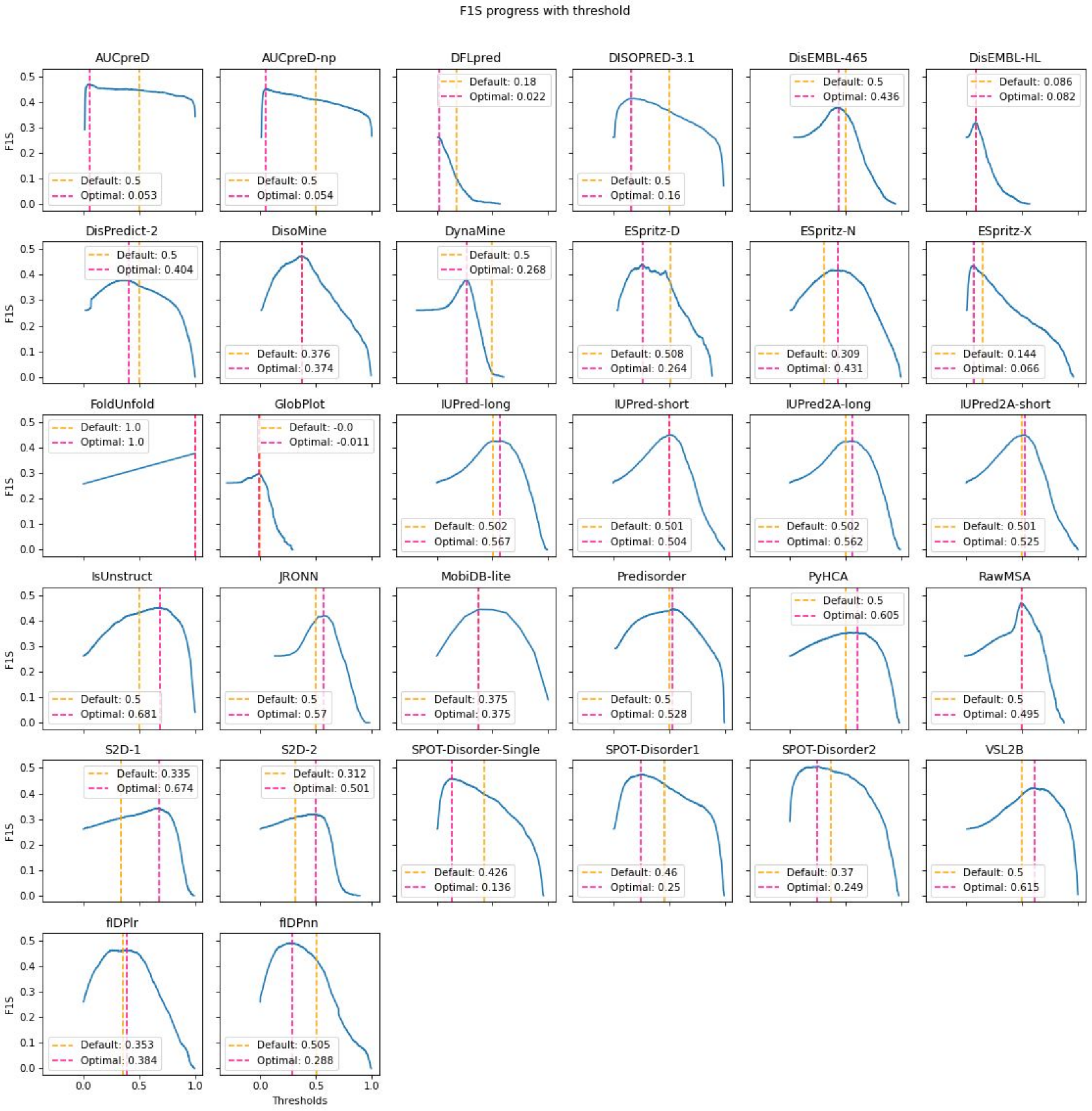

Supplementary Figure 26. F1-score progress with threshold for prokaryotic proteins in the *DisProt* dataset.

F1-score progress (y-axis) with increasing threshold value (x-axis) for each predictor calculated on prokaryotic proteins on the *DisProt* dataset.

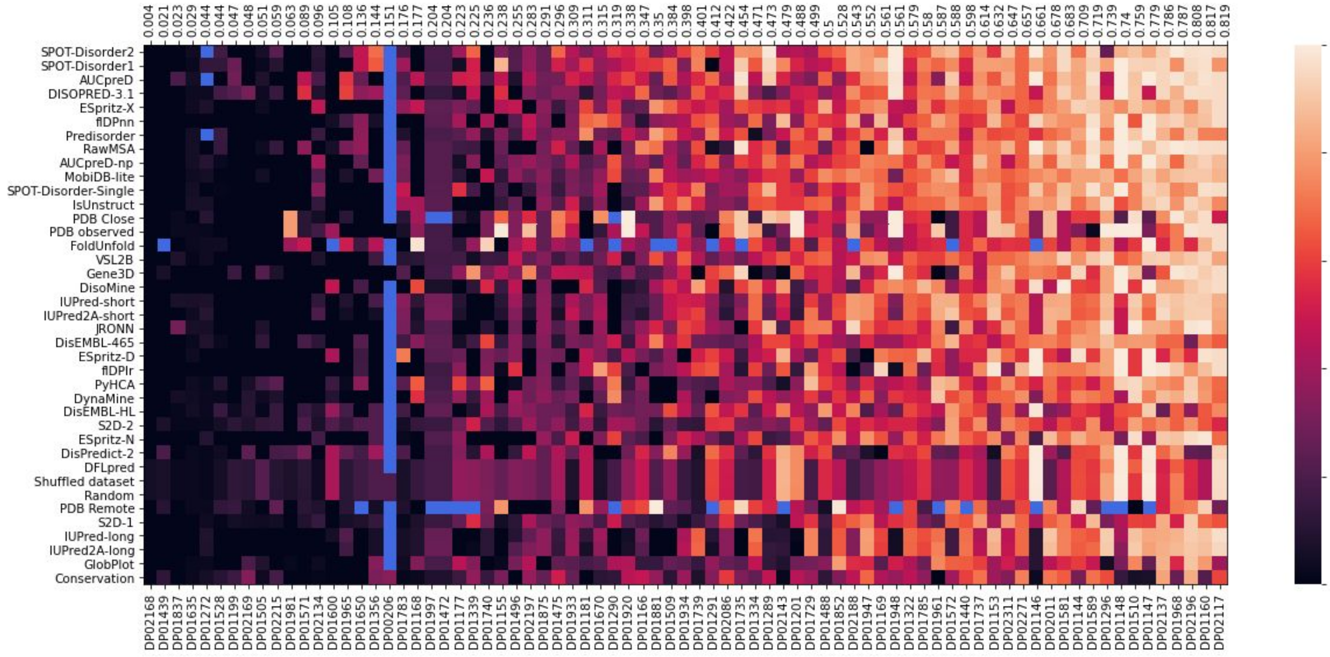

Supplementary Figure 27: F_{Max} per protein for prokaryotic proteins in the *DisProt* dataset.
F_{Max} of each target (x-axis, bottom labels) from each predictor (y-axis). Targets are sorted by average F_{Max} (x-axis, top labels). Calculated on prokaryotic proteins of the *DisProt* dataset. Missing values are in blue.

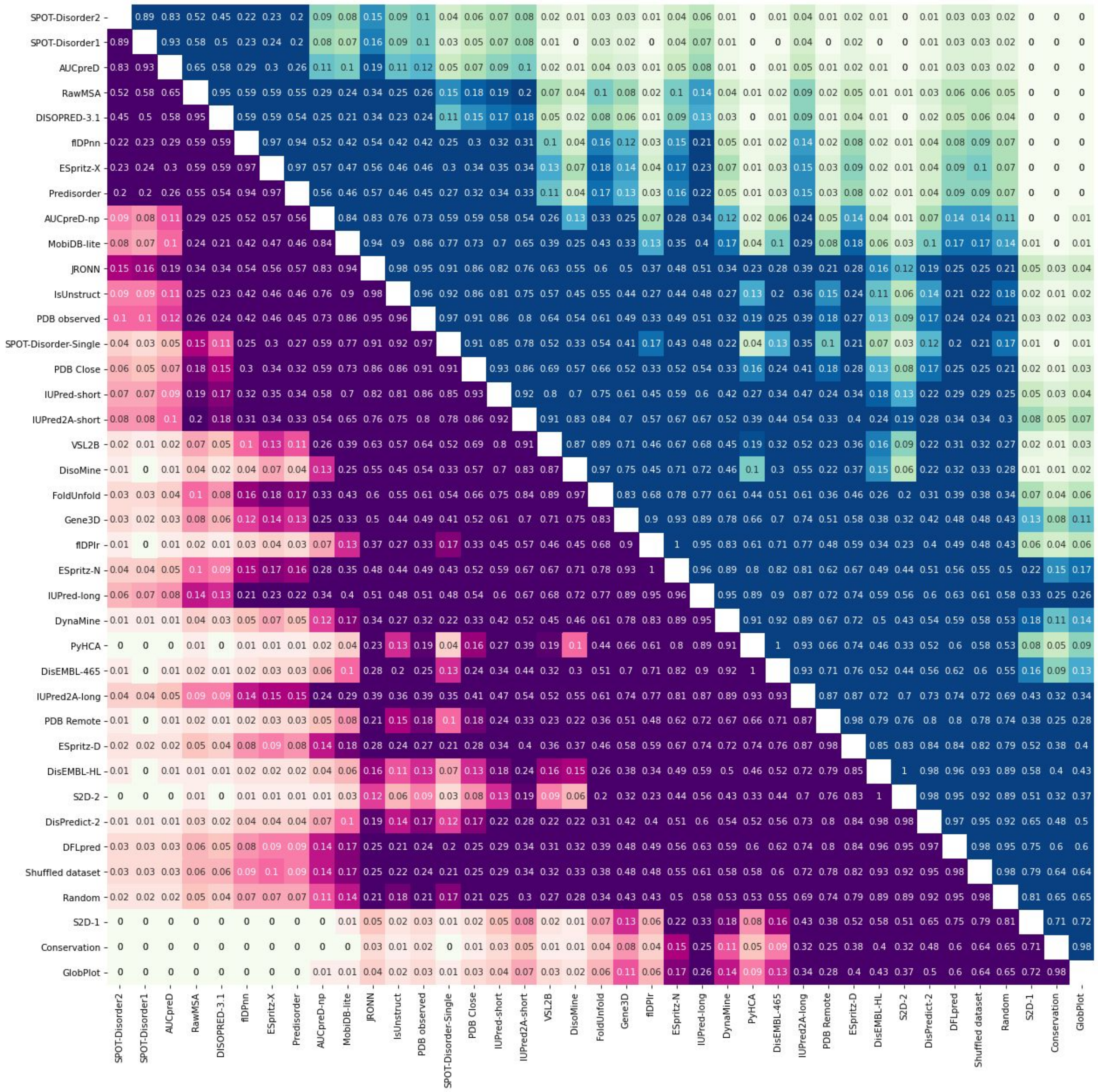

Supplementary Figure 28. Overall average ranking of all predictors and baselines for prokaryotic proteins in the *DisProt* dataset.
Heatmap of the T-test p-value associated to the statistical significance of the difference between ranking distribution of predictors. A ranking distribution for a predictor is the position of that predictor in its ranking for the following metrics: 'bac', 'f1s', 'fpr', 'mcc', 'ppv', 'tpr', 'tnr'. Metrics are calculated per target and with predictors threshold optimized by F1-Score.

DisProt-PDB dataset

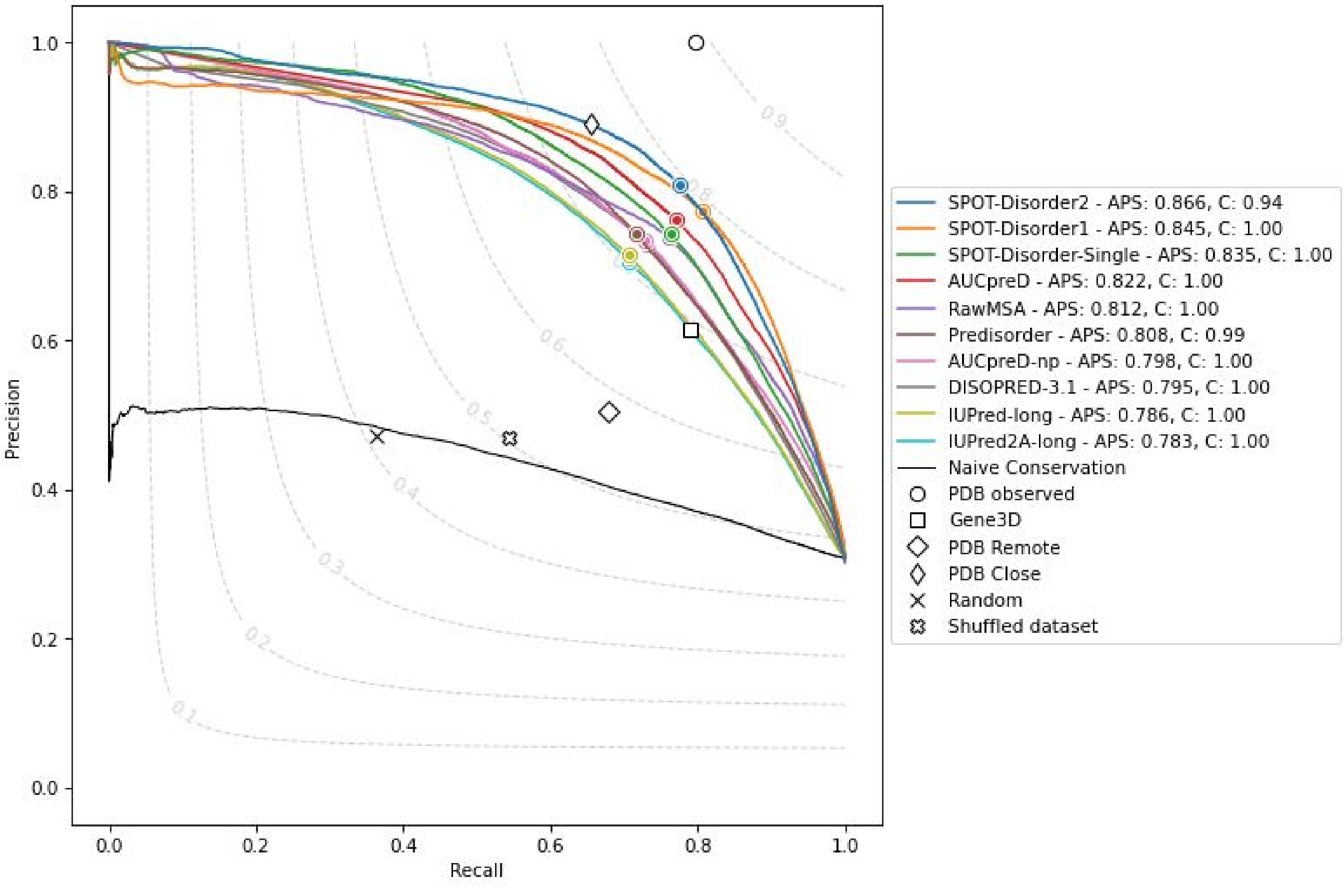

Supplementary Figure 29: Precision recall curve in the *DisProt-PDB* dataset.
Precision (y-axis) recall (x-axis) curves of the 10 best ranking methods. Ranking is based on their APS (average precision score) on the *DisProt-PDB* dataset.

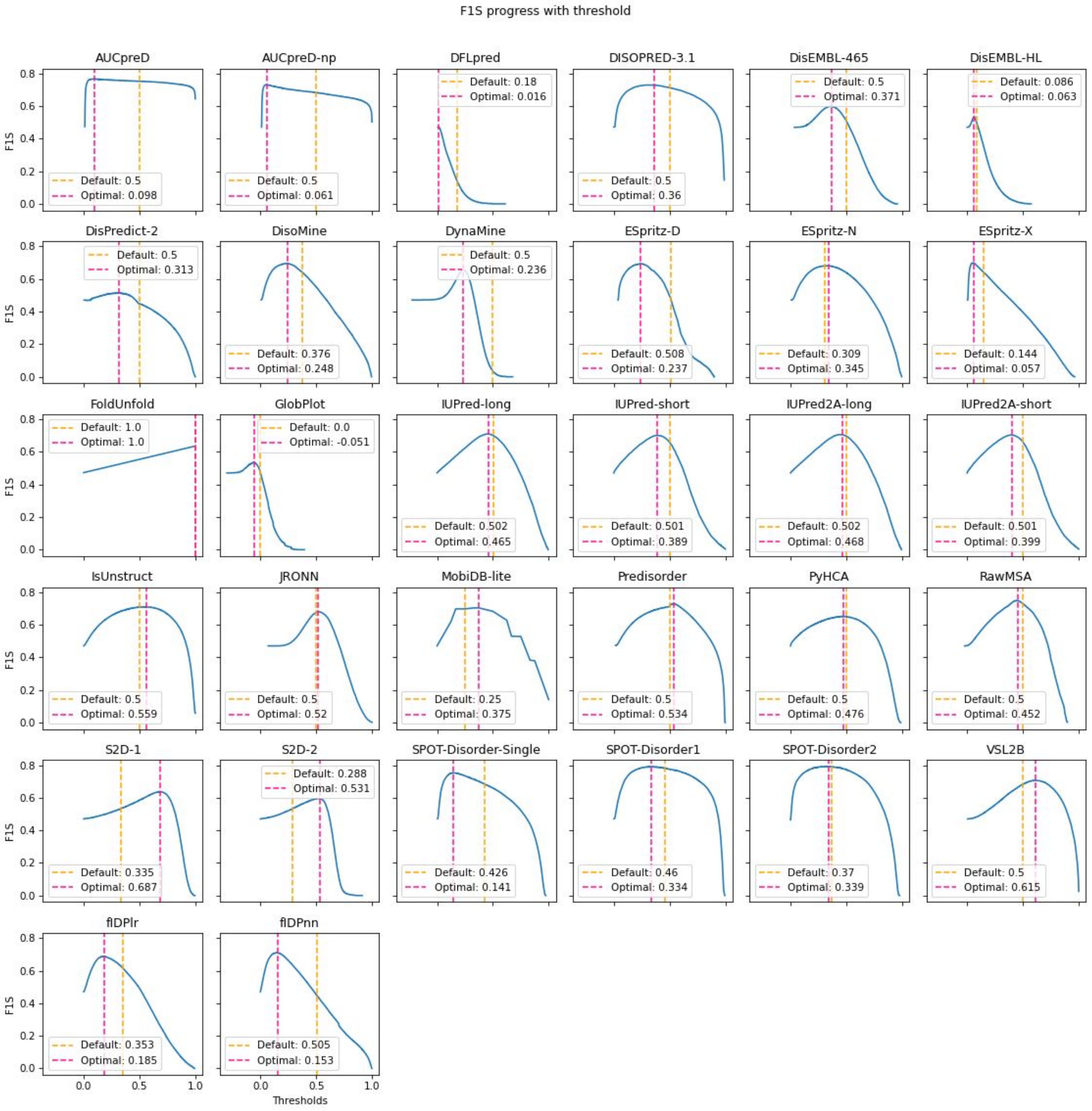

Supplementary Figure 30. F1-score progress with threshold in the *DisProt-PDB* dataset.
F1-score progress (y-axis) with increasing threshold value (x-axis) for each predictor on the *DisProt-PDB* dataset.

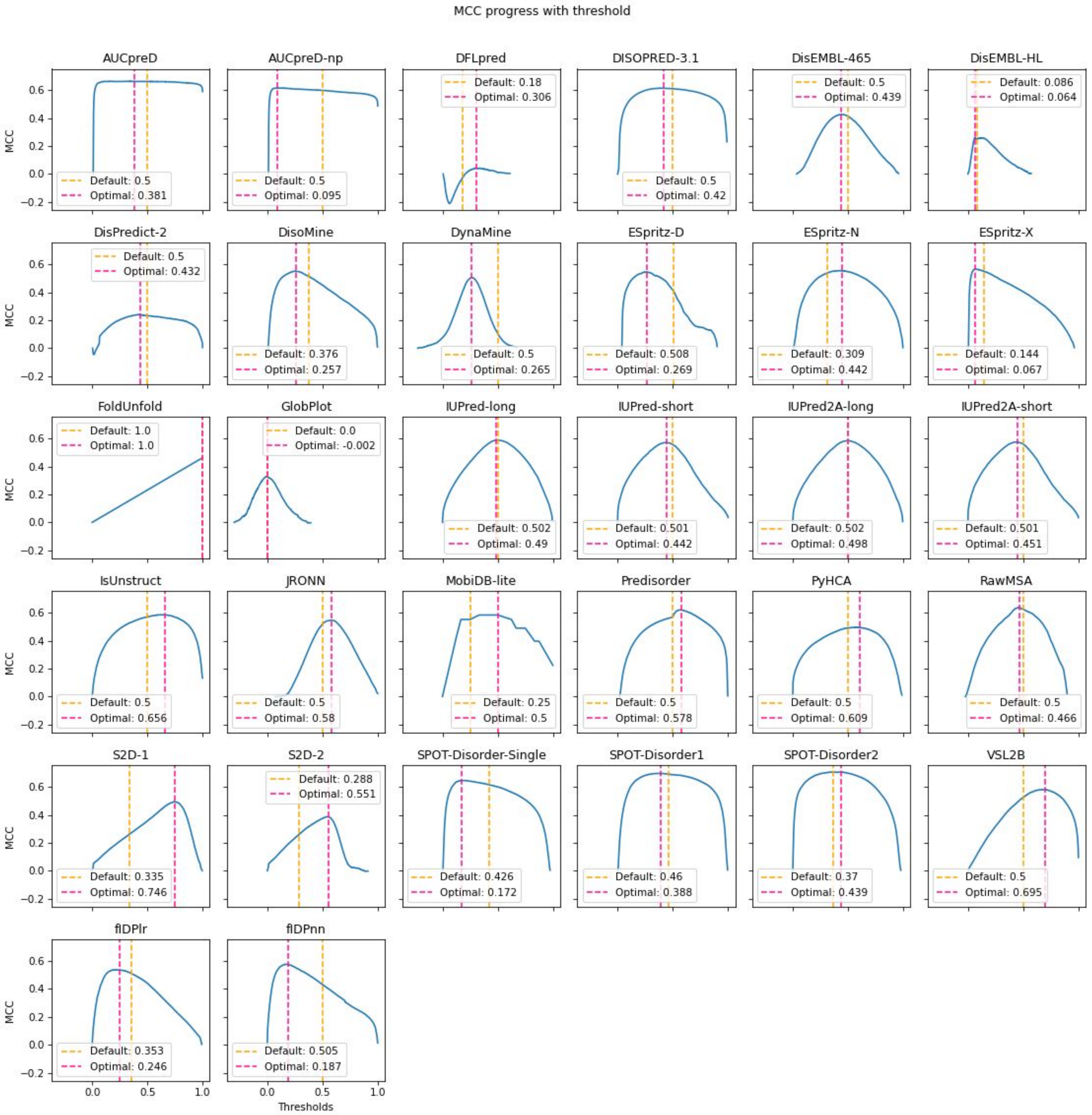

Supplementary Figure 31. MCC progress with threshold in the *DisProt-PDB* dataset.
MCC progress (y-axis) with increasing threshold value (x-axis) for each predictor on the *DisProt-PDB* dataset.

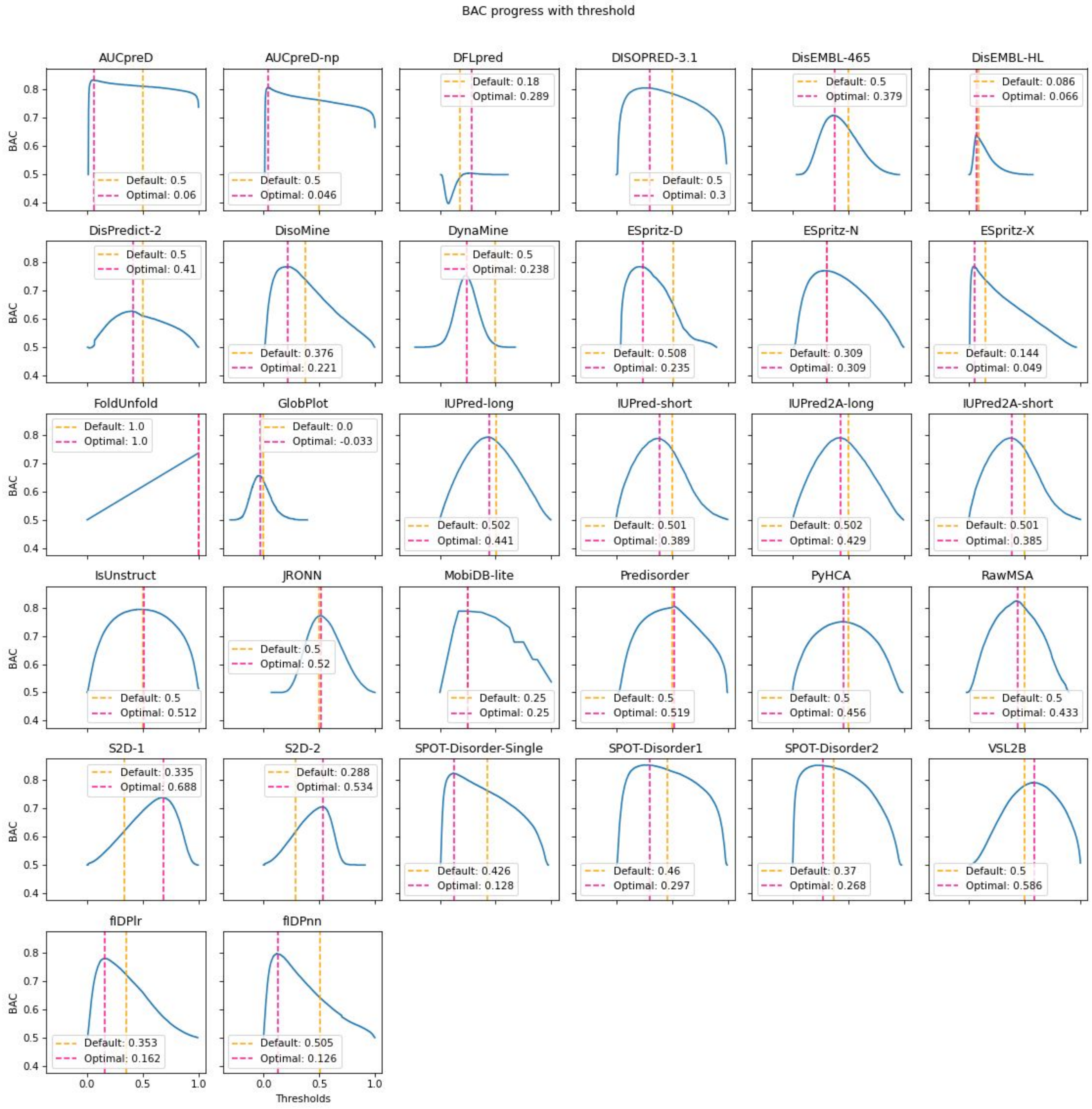

Supplementary Figure 32. Balanced accuracy progress with threshold in the *DisProt-PDB* dataset.

Balanced accuracy progress (y-axis) with increasing threshold value (x-axis) for each predictor on the *DisProt-PDB* dataset.

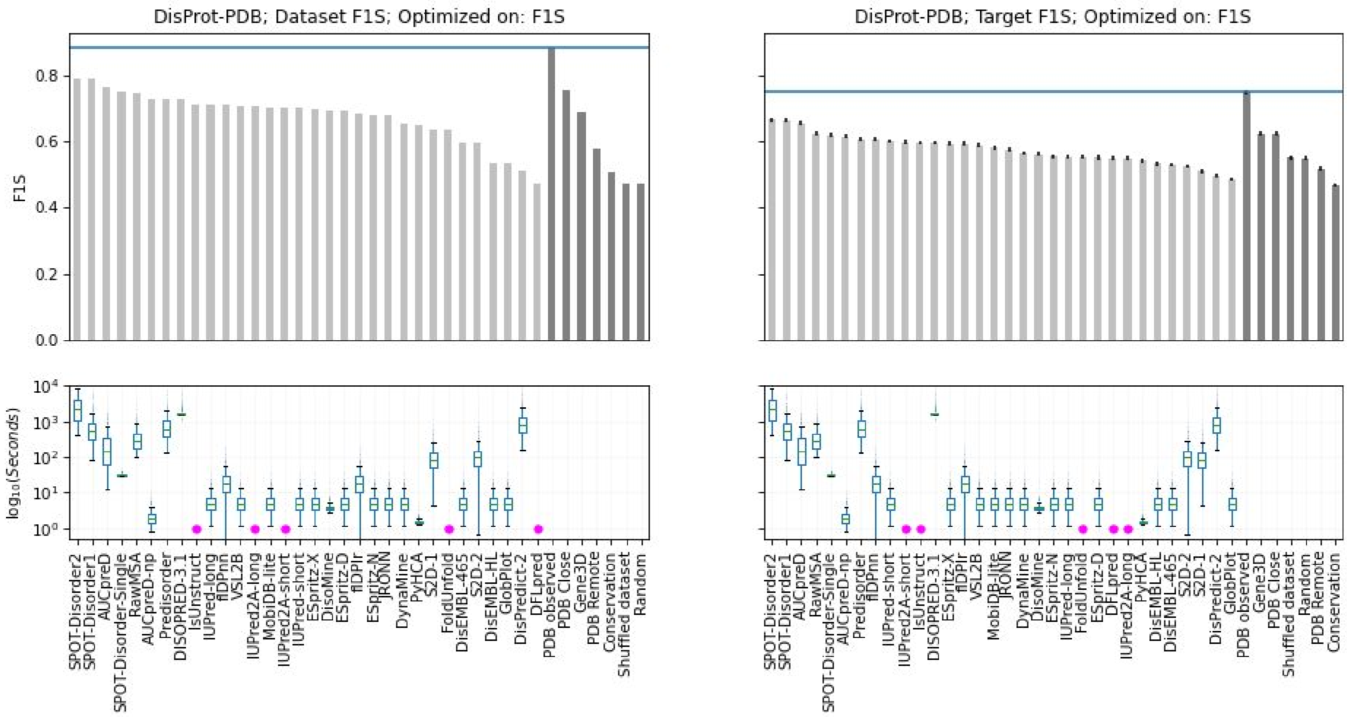

Supplementary Figure 33: F_{Max} in the *DisProt-PDB* dataset.

F_{Max} calculated on the whole dataset with confidence intervals as error bars (left) and averaged over proteins with Standard-Error as error bars (right). Calculated on *DisProt-PDB* dataset (n= 646 proteins). Boxplots are defined as follows: the middle value of the dataset is the median (Q2/50th Percentile). The box boundaries are the 1st quartile (Q1/25th Percentile) and 3rd quartile (Q3/75th Percentile) respectively; Maximum is Q3 + 1.5*(Q3-Q1) and Minimum is Q1 -1.5*(Q3-Q1). Outliers are hidden for clarity. Magenta dots indicate that the whole distribution of execution-times is lower than 1 second.

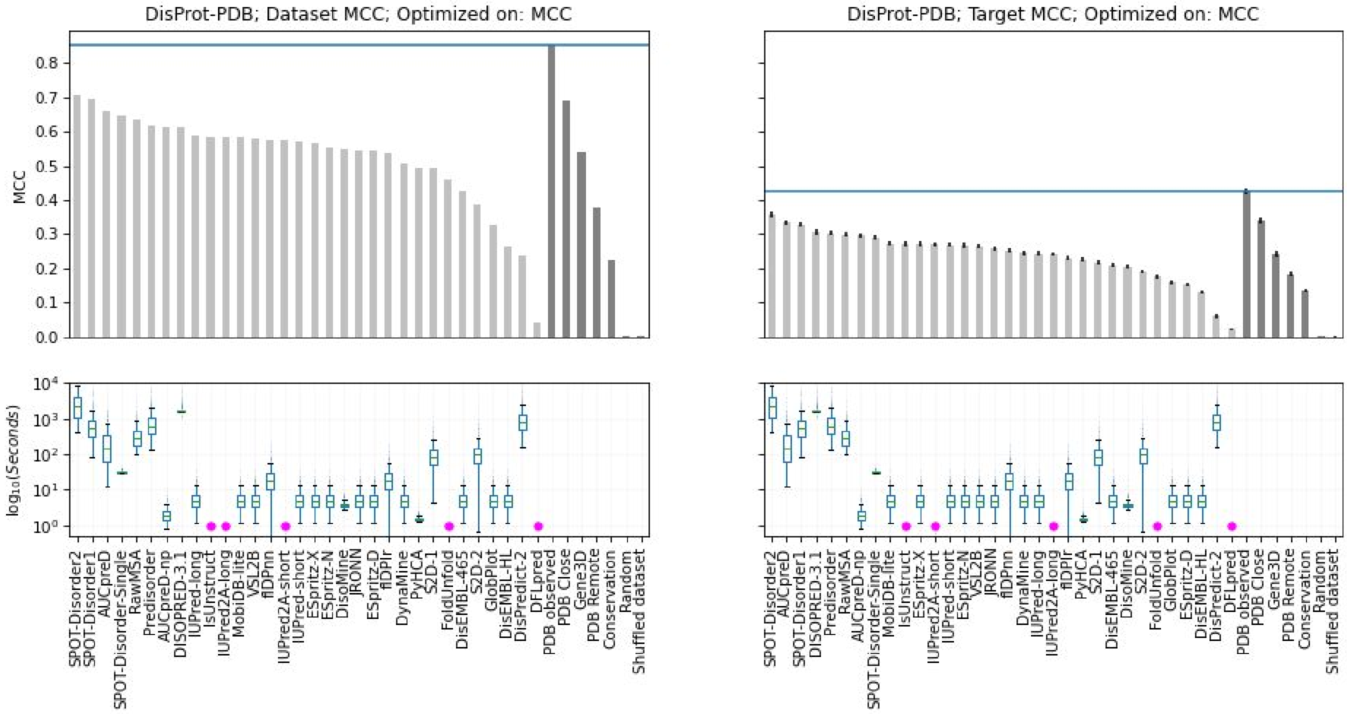

Supplementary Figure 34: MCC in the *DisProt-PDB* dataset.

MCC calculated on the whole dataset with confidence intervals as error bars (left) and averaged over proteins with Standard-Error as error bars (right). Calculated on *DisProt-PDB* dataset (n= 646 proteins).

Predictors threshold is optimized on MCC. Boxplots are defined as follows: the middle value of the dataset is the median (Q2/50th Percentile). The box boundaries are the 1st quartile (Q1/25th Percentile) and 3rd quartile (Q3/75th Percentile) respectively; Maximum is Q3 + 1.5*(Q3-Q1) and Minimum is Q1 -1.5*(Q3-Q1). Outliers are hidden for clarity. Magenta dots indicate that the whole distribution of execution-times is lower than 1 second.

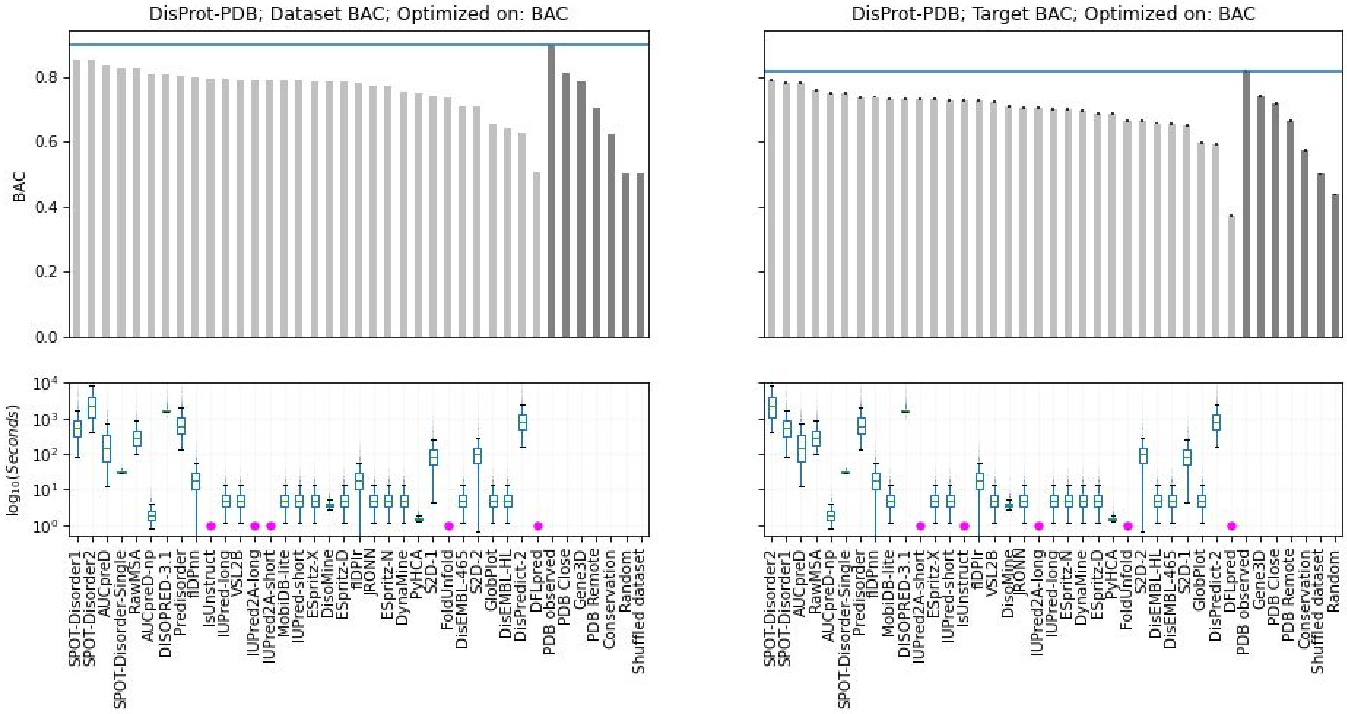

Supplementary Figure 35: Balanced accuracy in the *DisProt-PDB* dataset.
Balanced accuracy calculated on the whole dataset with confidence intervals as error bars (left) and averaged over proteins with Standard-Error as error bar (right). Calculated on *DisProt-PDB* dataset (n= 646 proteins). Predictors threshold is optimized on Balanced accuracy. Boxplots are defined as follows: the middle value of the dataset is the median (Q2/50th Percentile). The box boundaries are the 1st quartile (Q1/25th Percentile) and 3rd quartile (Q3/75th Percentile) respectively; Maximum is Q3 + 1.5*(Q3-Q1) and Minimum is Q1 -1.5*(Q3-Q1). Outliers are hidden for clarity. Magenta dots indicate that the whole distribution of execution-times is lower than 1 second.

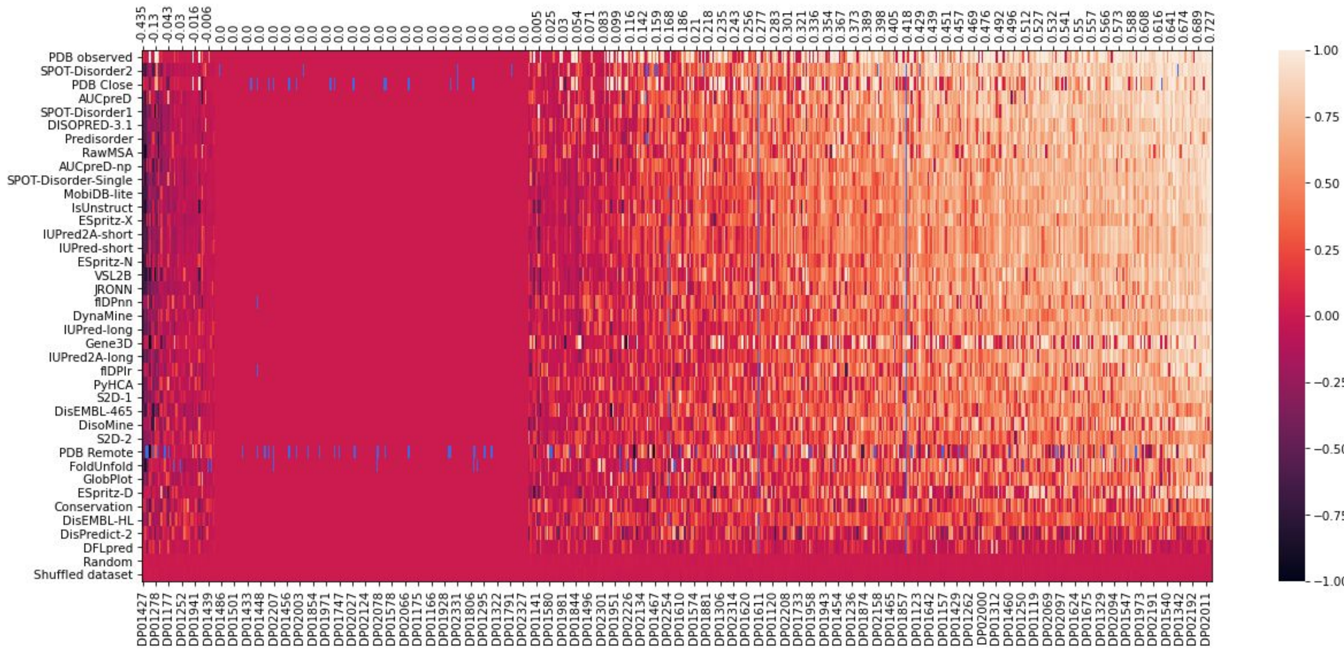

Supplementary Figure 36: MCC per protein in the *DisProt-PDB* dataset.

MCC of each target (x-axis, bottom labels, not all labels are visible) from each predictor (y-axis). Targets are sorted by average MCC (x-axis, top labels). Calculated on *DisProt-PDB* dataset. Predictors threshold is optimized on MCC. Missing values are in blue.

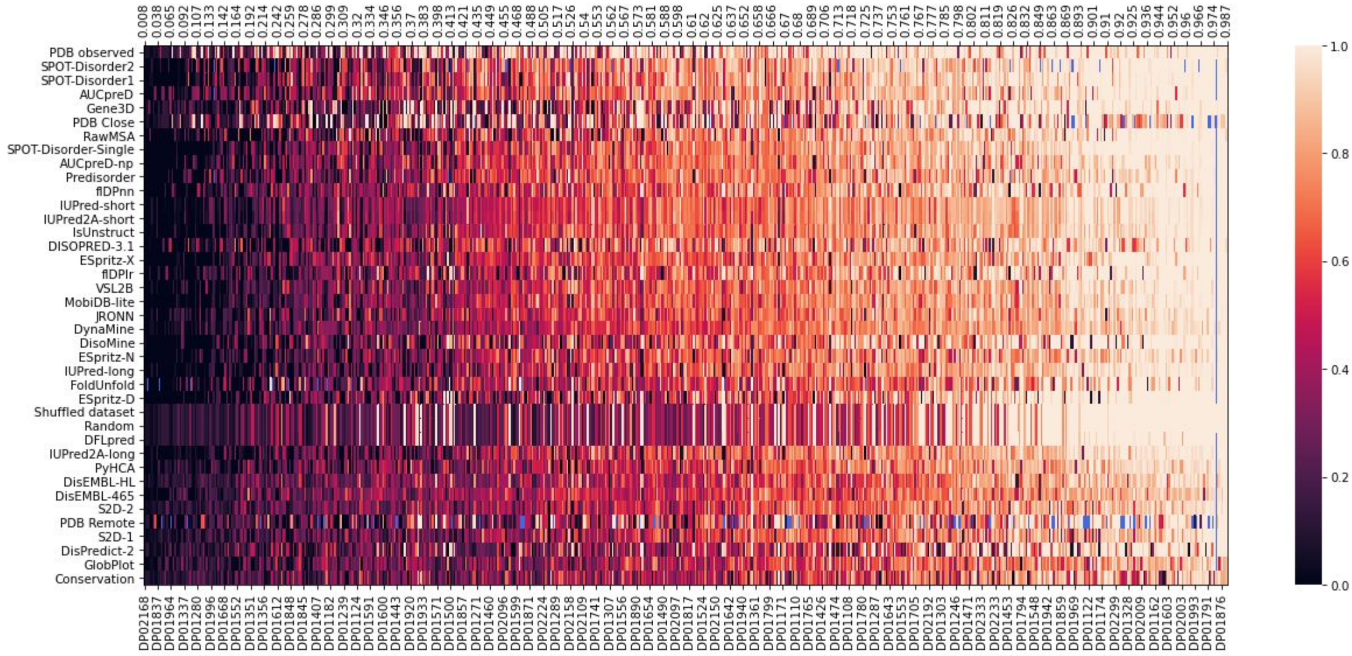

Supplementary Figure 37: F_{Max} per protein in the *DisProt-PDB* dataset.

F_{Max} of each target (x-axis, bottom labels, not all labels are visible) from each predictor (y-axis). Targets are sorted by average F_{Max} (x-axis, top labels). Calculated on *DisProt-PDB* dataset. Missing values are in blue.

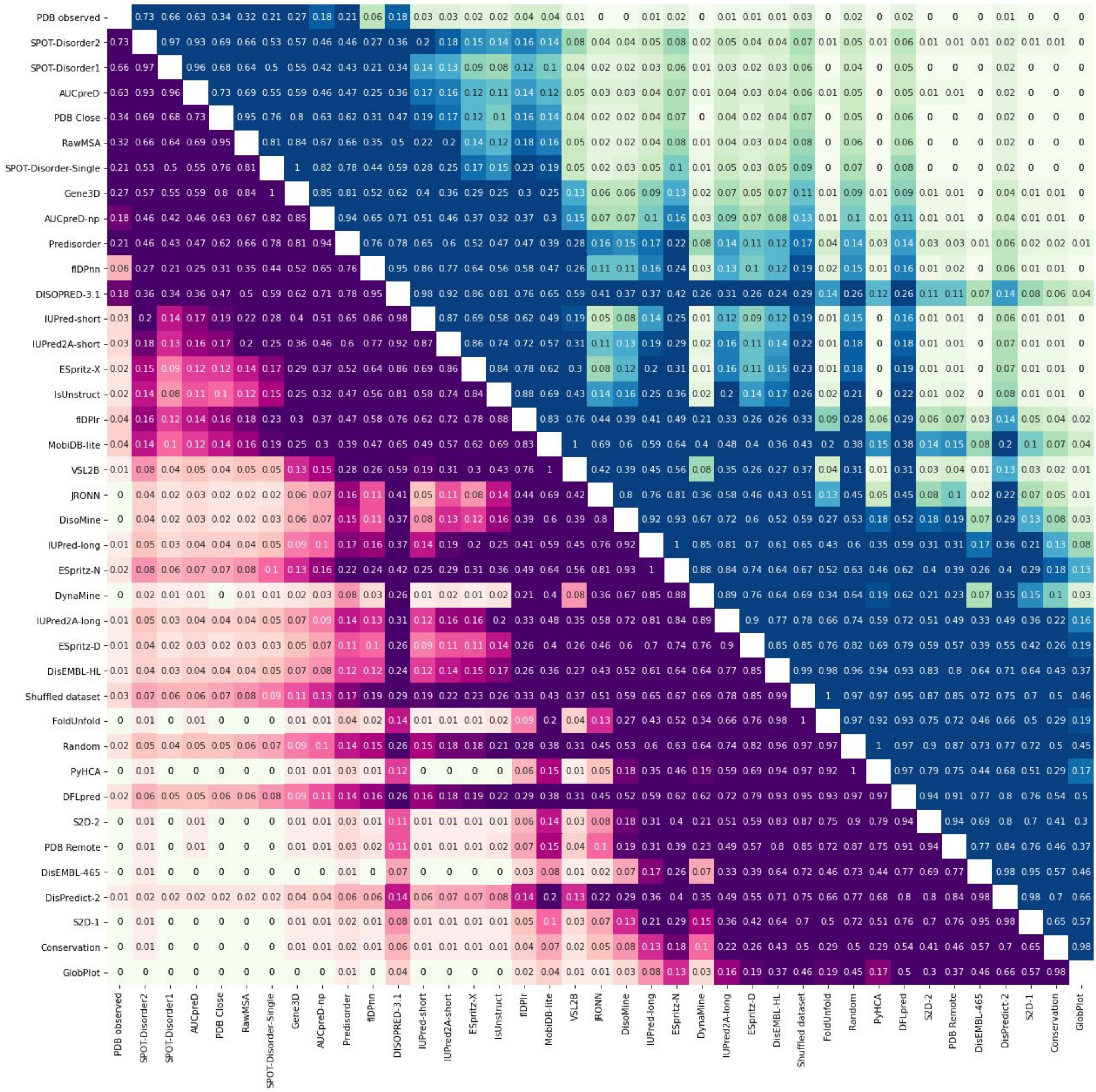

Supplementary Figure 38. Overall average ranking of all predictors and baselines in the *DisProt-PDB* dataset.

Heatmap of the T-test p-value associated to the statistical significance of the difference between ranking distribution of predictors. A ranking distribution for a predictor is the position of that predictor in its ranking for each metric. Metrics used are: bac, f1s, fpr, mcc, ppv, tpr, tnr; they are calculated with predictors threshold optimized by F1-Score.

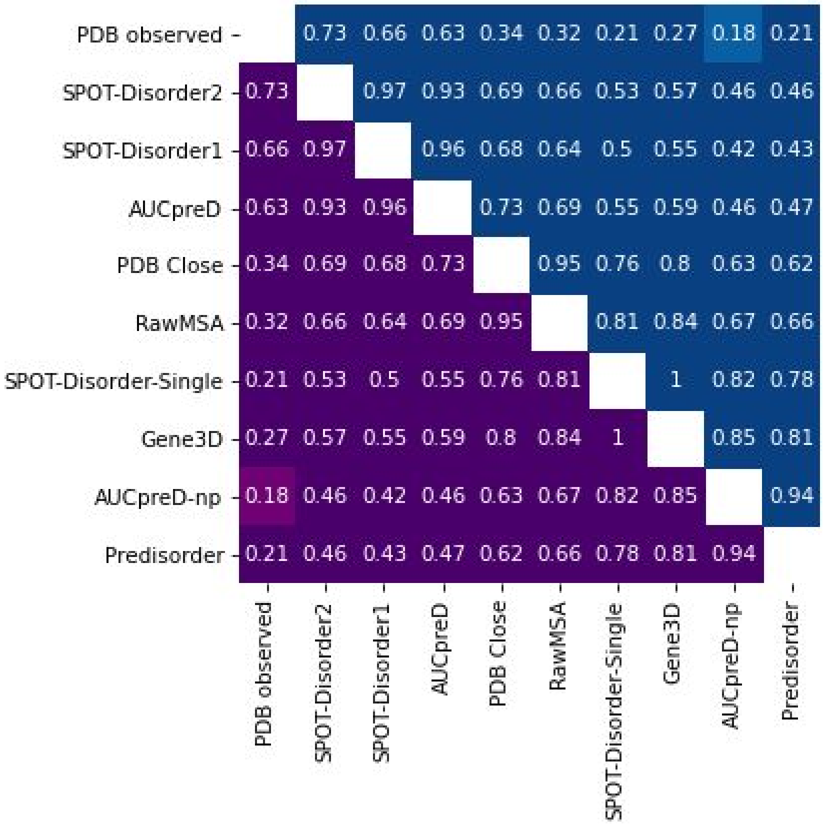

Supplementary Figure 39. Overall average ranking of the 10 best ranking predictors and baselines in the *DisProt-PDB* dataset.

Heatmap of the T-test p-value associated to the statistical significance of the difference between ranking distribution of predictors. A ranking distribution for a predictor is the position of that predictor in its ranking for each metric. Metrics used are: bac, f1s, fpr, mcc, ppv, tpr, tnr; they are calculated with predictors threshold optimized by F1-Score.

Mammals

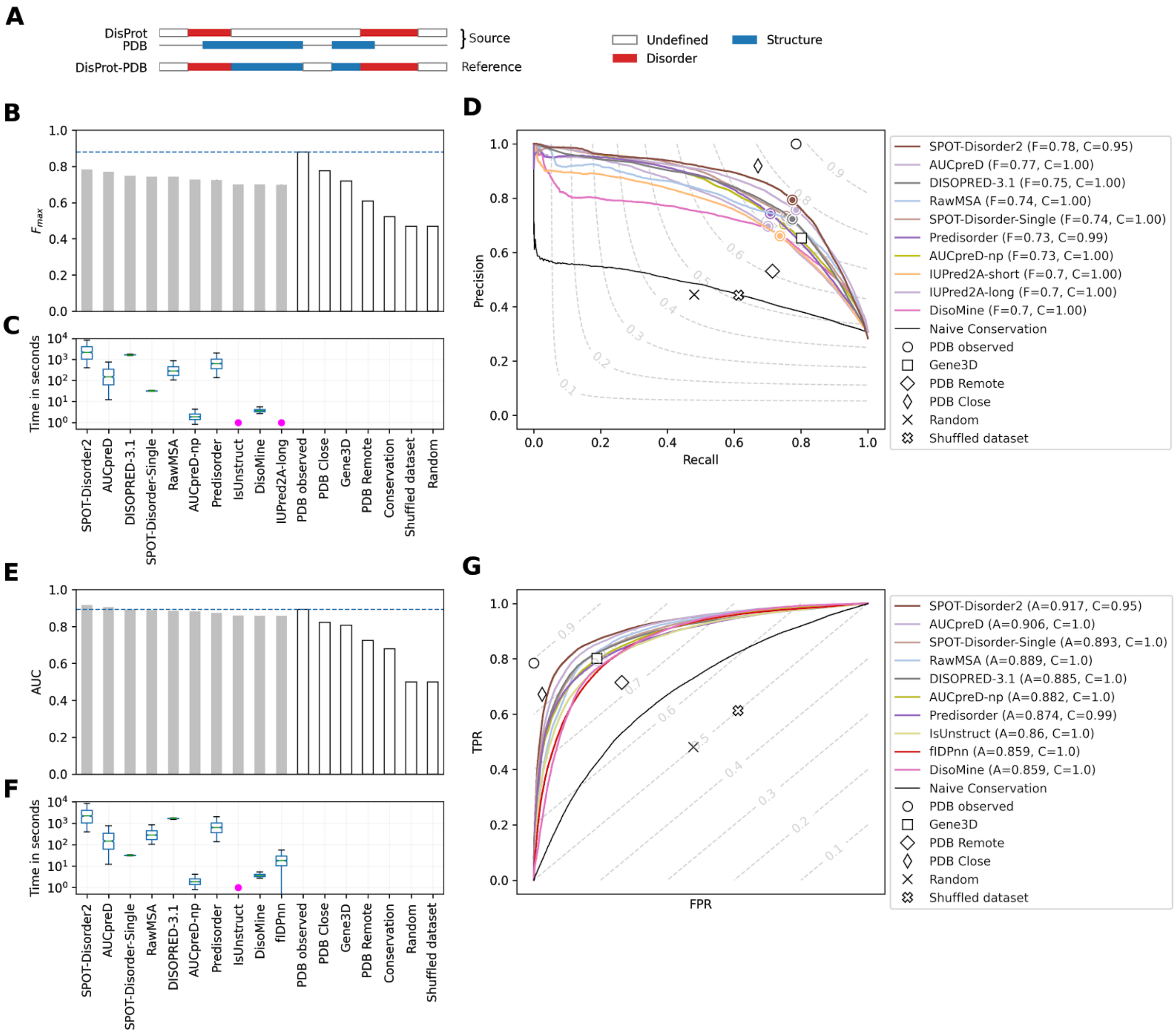

Supplementary Figure 40. Prediction success and CPU times for the ten top-ranking disorder predictors for mammalian proteins in the *DisProt-PDB* dataset.

Prediction success and CPU times for the ten top-ranking disorder predictors for mammalian proteins in the *DisProt-PDB* dataset (n= 368 proteins). Reference used (*DisProt-PDB*) in the analysis and how it is obtained (panel A). Performance of predictors expressed as maximum F1-Score across all thresholds (F_{max}) (panel B) and AUC (panel E) for the top ten best ranking methods (light gray) and baselines (white) and the distribution of execution time per-target (panels C, F) using *DisProt-PDB* dataset. The horizontal line in panels B, E indicates the F_{max} and AUC of the best baseline, respectively. Precision-Recall (panel D) and ROC curves (panel G) of ten top-ranking methods and baselines using *DisProt-PDB* dataset, with level curves of the F1-Score and Balanced accuracy, respectively. Boxplots in panels **C, F** are defined as follows: the middle value of the dataset is the median (Q2/50th Percentile). The box boundaries are the 1st quartile (Q1/25th Percentile) and 3rd quartile (Q3/75th Percentile) respectively; Maximum is Q3 + 1.5*(Q3-Q1) and Minimum is Q1 -1.5*(Q3-Q1). Outliers are hidden for clarity. Magenta dots on panels C, F indicate that the whole distribution of execution-times is lower than 1 second.

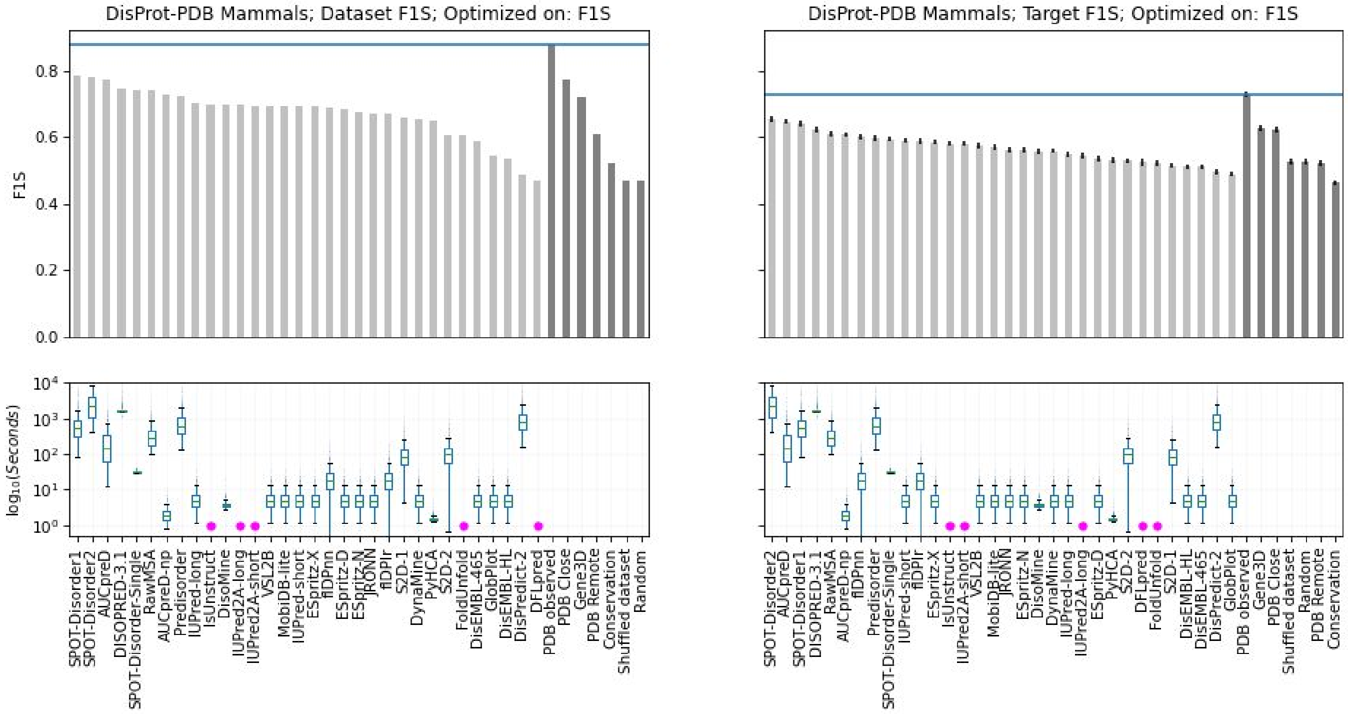

Supplementary Figure 41: F_{Max} for mammalian proteins in the *DisProt-PDB* dataset.
F_{Max} calculated on the whole dataset with confidence intervals as error bars (left) and averaged over proteins with Standard-Error as error bars (right). Calculated on mammalian proteins of the *DisProt-PDB* dataset (n= 368 proteins). Boxplots are defined as follows: the middle value of the dataset is the median (Q2/50th Percentile). The box boundaries are the 1st quartile (Q1/25th Percentile) and 3rd quartile (Q3/75th Percentile) respectively; Maximum is Q3 + 1.5*(Q3-Q1) and Minimum is Q1 -1.5*(Q3-Q1). Outliers are hidden for clarity. Magenta dots indicate that the whole distribution of execution-times is lower than 1 second.

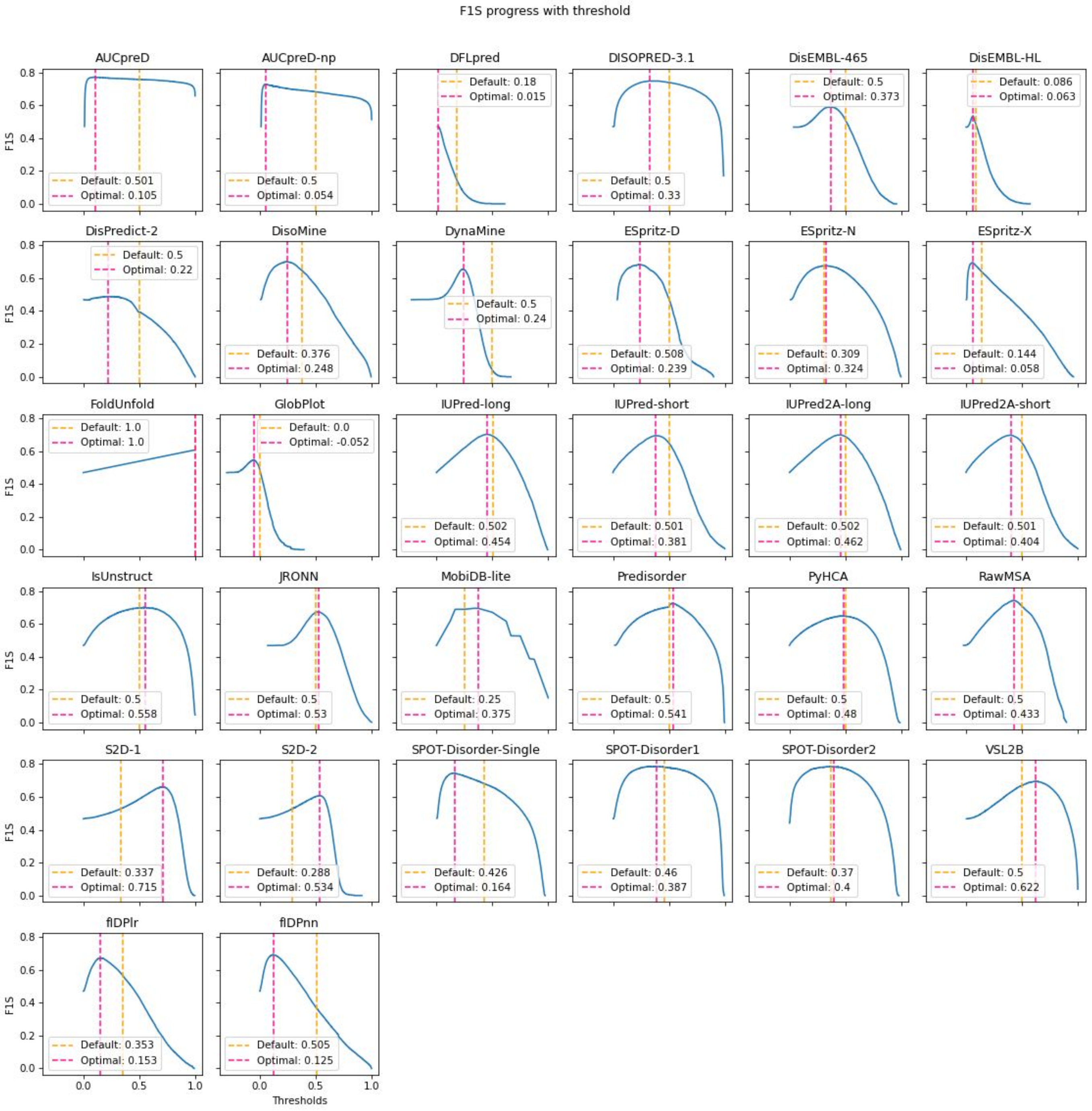

Supplementary Figure 42. F1-Score progress with threshold for mammalian proteins in the *DisProt-PDB* dataset.

F1-score progress (y-axis) with increasing threshold value (x-axis) for each predictor calculated on mammalian proteins on the *DisProt-PDB* dataset.

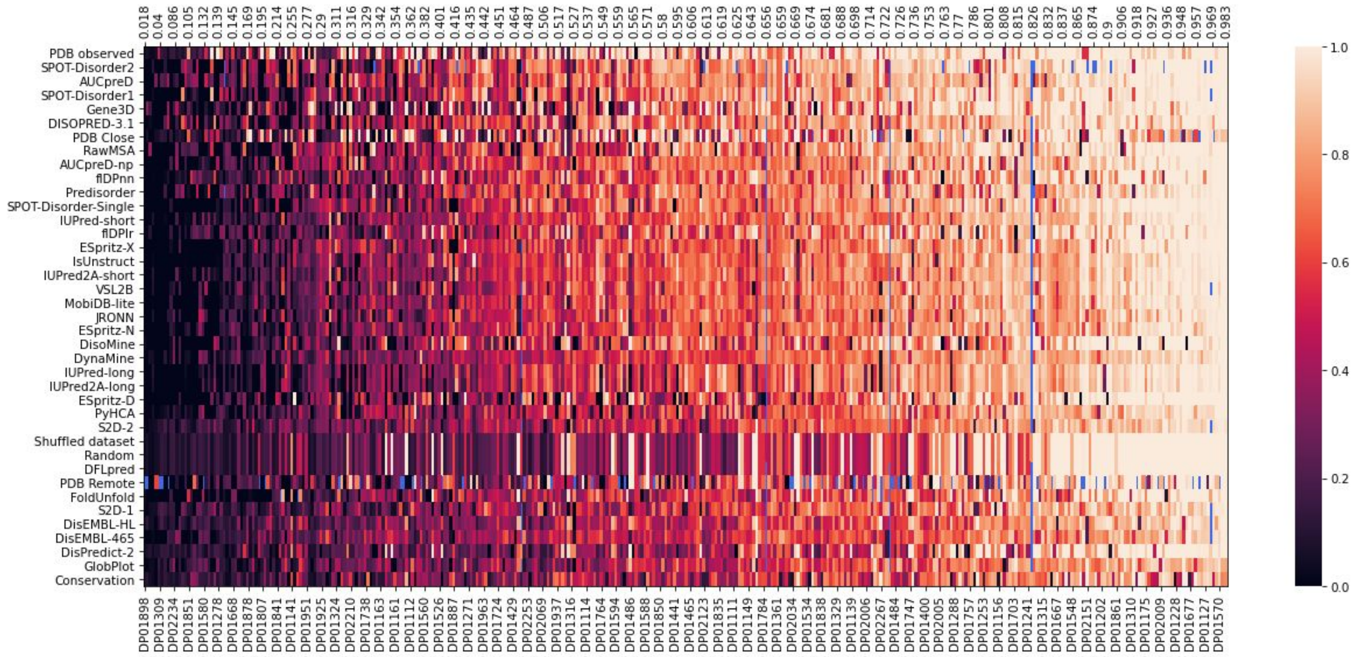

Supplementary Figure 43: F_{Max} per protein for mammalian proteins in the *DisProt-PDB* dataset. F_{Max} of each target (x-axis, bottom labels) from each predictor (y-axis). Targets are sorted by average F_{Max} (x-axis, top labels). Calculated on mammalian proteins of the *DisProt-PDB* dataset. Missing values are in blue.

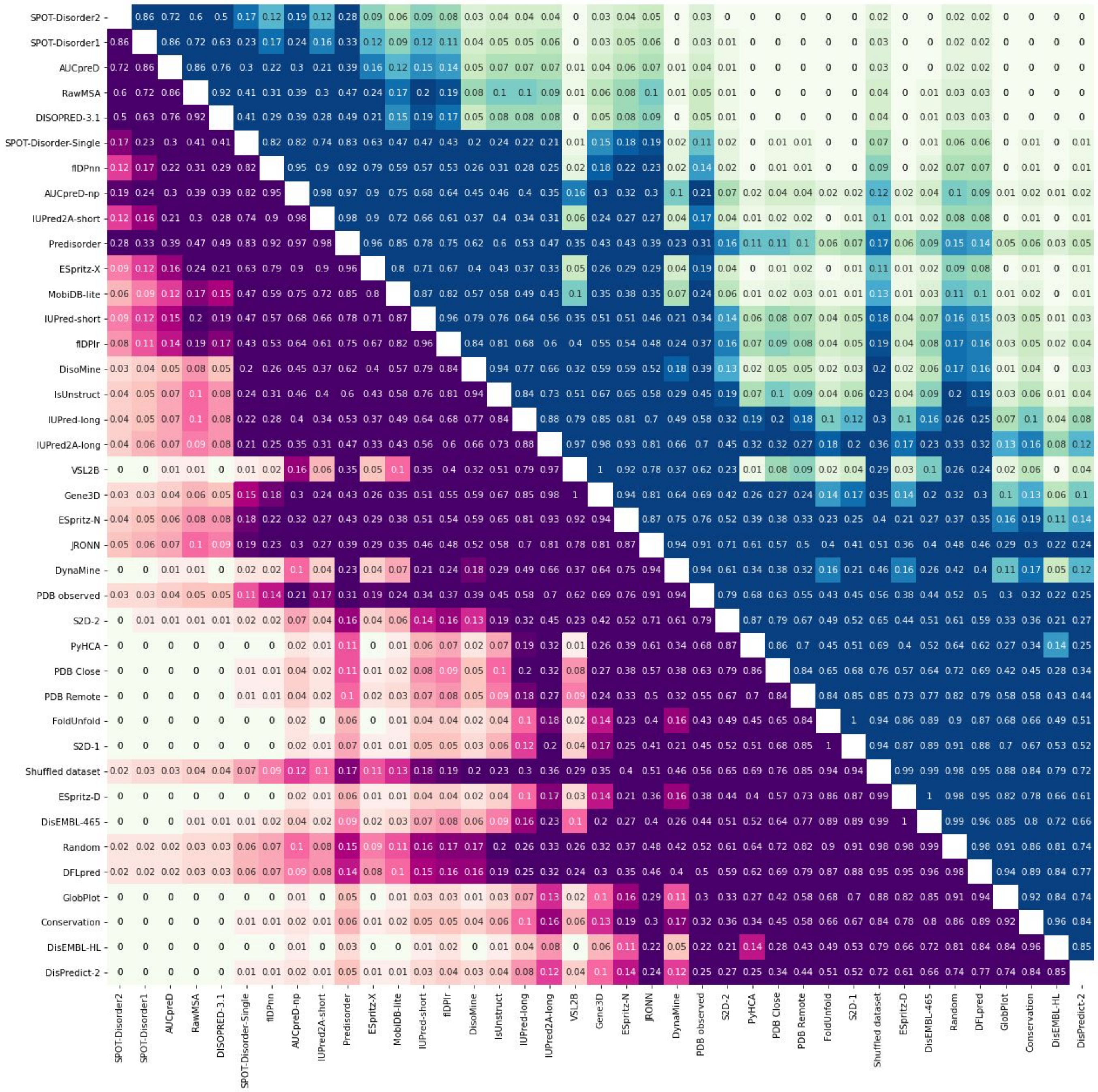

Supplementary Figure 44. Overall average ranking of all predictors and baselines for mammalian proteins in the *DisProt-PDB* dataset.

Heatmap of the T-test p-value associated to the statistical significance of the difference between ranking distribution of predictors. A ranking distribution for a predictor is the position of that predictor in its ranking for the following metrics: 'bac', 'f1s', 'fpr', 'mcc', 'ppv', 'tpr', 'tnr'. Metrics are calculated per target and with predictors threshold optimized by F1-Score.

Prokaryotes

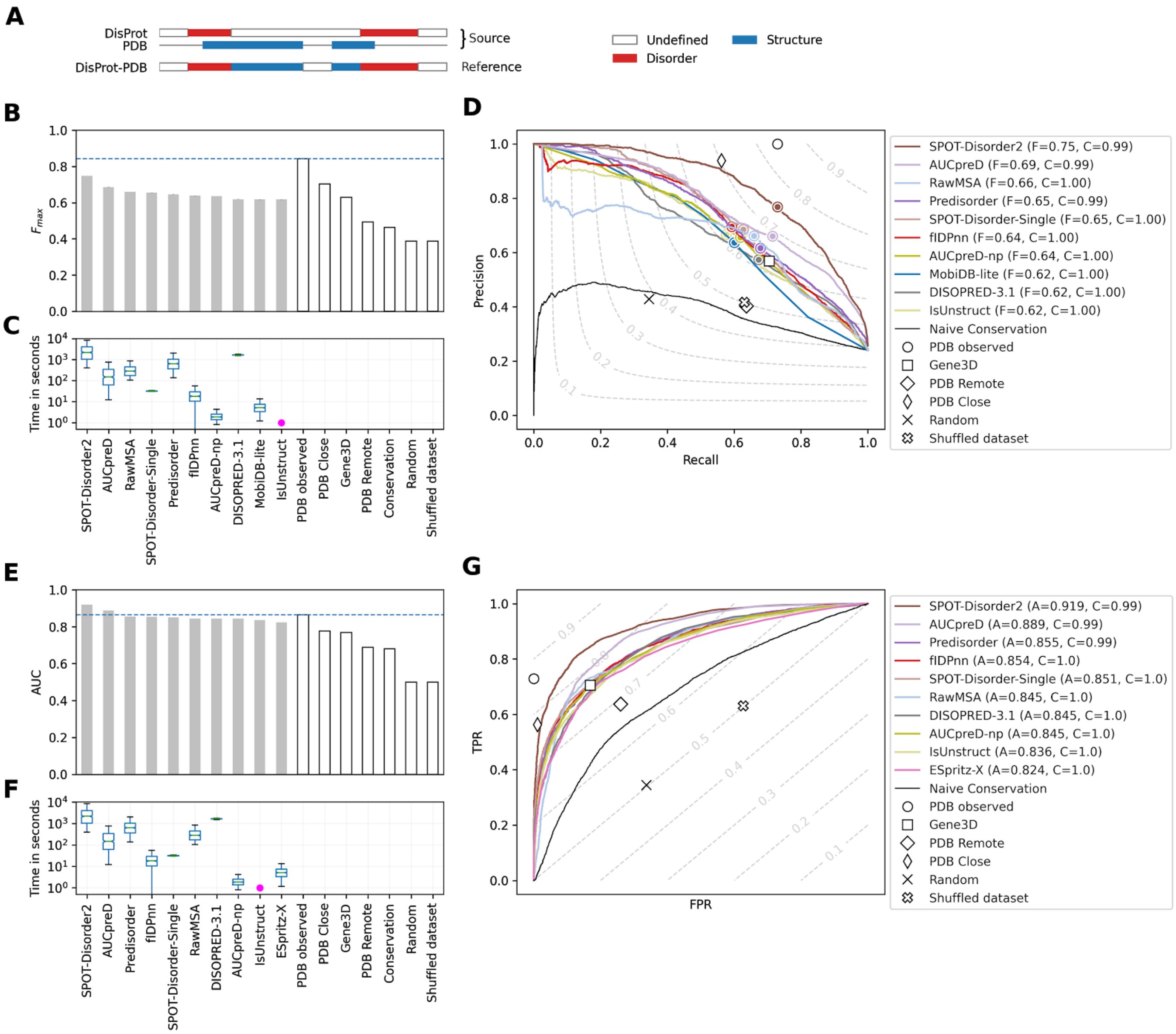

Supplementary Figure 45. Prediction success and CPU times for the ten top-ranking disorder predictors for prokaryotic proteins in the *DisProt-PDB* dataset.

Prediction success and CPU times for the ten top-ranking disorder predictors for prokaryotic proteins in the *DisProt-PDB* dataset (n= 77 proteins). Reference used (*DisProt-PDB*) in the analysis and how it is obtained (panel A). Performance of predictors expressed as maximum F1-Score across all thresholds (F_{max}) (panel B) and AUC (panel E) for the top ten best ranking methods (light gray) and baselines (white) and the distribution of execution time per-target (panels C, F) using *DisProt-PDB* dataset. The horizontal line in panels B, E indicates the F_{max} and AUC of the best baseline, respectively. Precision-Recall (panel D) and ROC curves (panel G) of ten top-ranking methods and baselines using *DisProt-PDB* dataset, with level curves of the F1-Score and Balanced accuracy, respectively. Boxplots in panels **C, F** are defined as follows: the middle value of the dataset is the median (Q2/50th Percentile). The box boundaries are the 1st quartile (Q1/25th Percentile) and 3rd quartile (Q3/75th Percentile) respectively; Maximum is Q3 + 1.5*(Q3-Q1) and Minimum is Q1 -1.5*(Q3-Q1). Outliers are hidden for clarity. Magenta dots on panels C, F indicate that the whole distribution of execution-times is lower than 1 second.

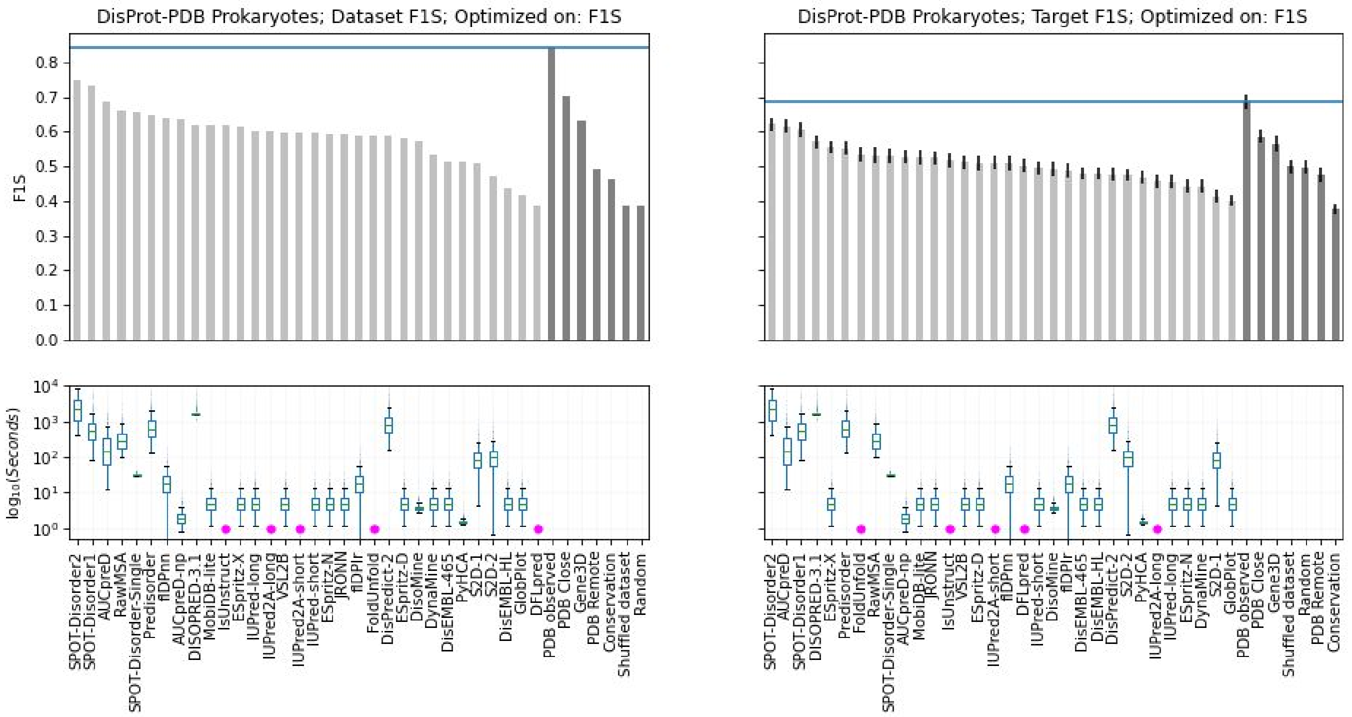

Supplementary Figure 46: F_{Max} for prokaryotic proteins in the *DisProt-PDB* dataset.
F_{Max} calculated on the whole dataset with confidence intervals as error bars (left) and averaged over proteins with Standard-Error as error bars (right). Calculated on prokaryotic proteins of the *DisProt-PDB* dataset (n= 77 proteins). Boxplots are defined as follows: the middle value of the dataset is the median (Q2/50th Percentile). The box boundaries are the 1st quartile (Q1/25th Percentile) and 3rd quartile (Q3/75th Percentile) respectively; Maximum is Q3 + 1.5*(Q3-Q1) and Minimum is Q1 -1.5*(Q3-Q1). Outliers are hidden for clarity. Magenta dots indicate that the whole distribution of execution-times is lower than 1 second.

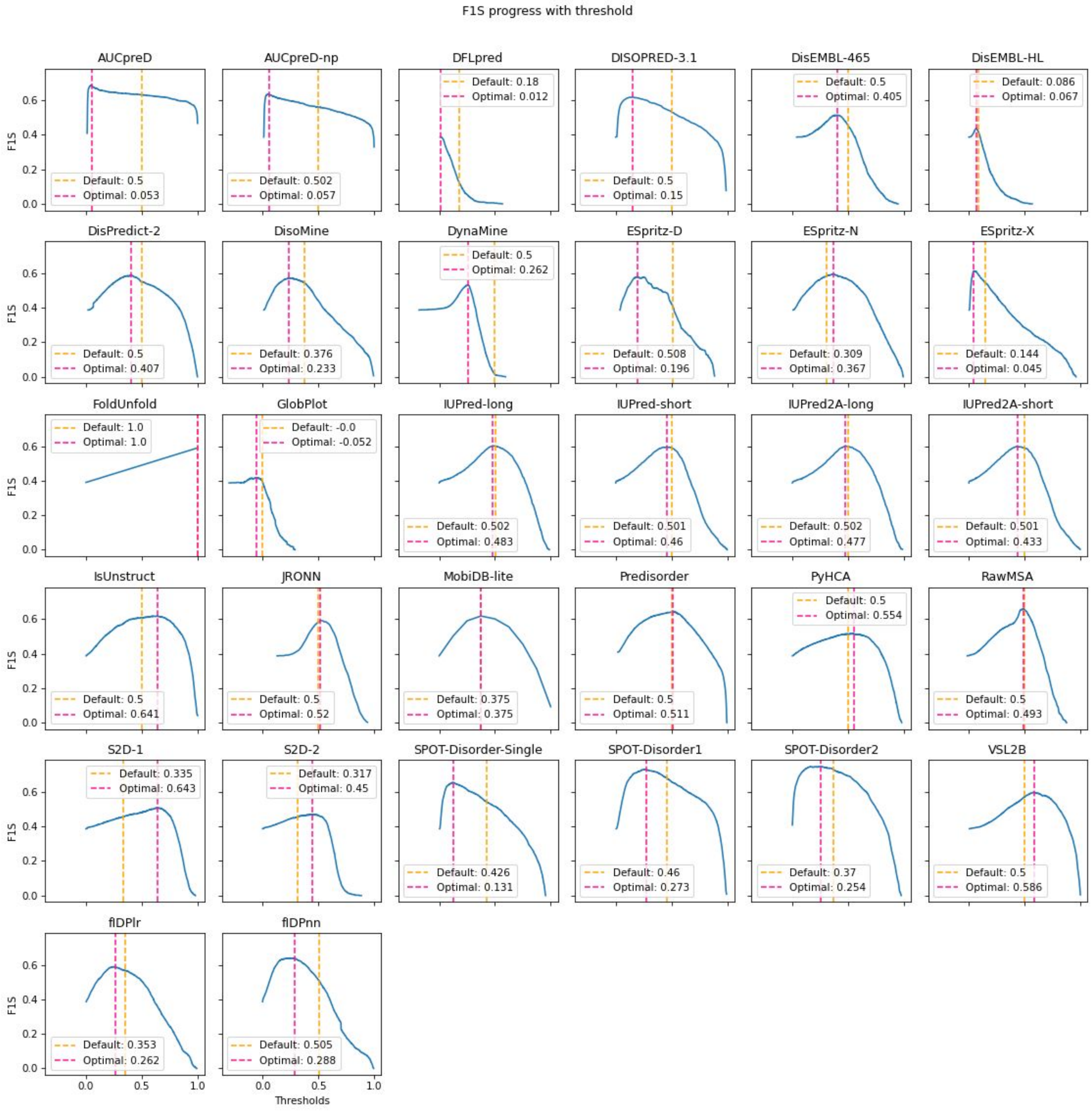

Supplementary Figure 47. F1-Score progress with threshold for prokaryotic proteins in the *DisProt-PDB* dataset.

F1-score progress (y-axis) with increasing threshold value (x-axis) for each predictor calculated on prokaryotic proteins on the *DisProt-PDB* dataset.

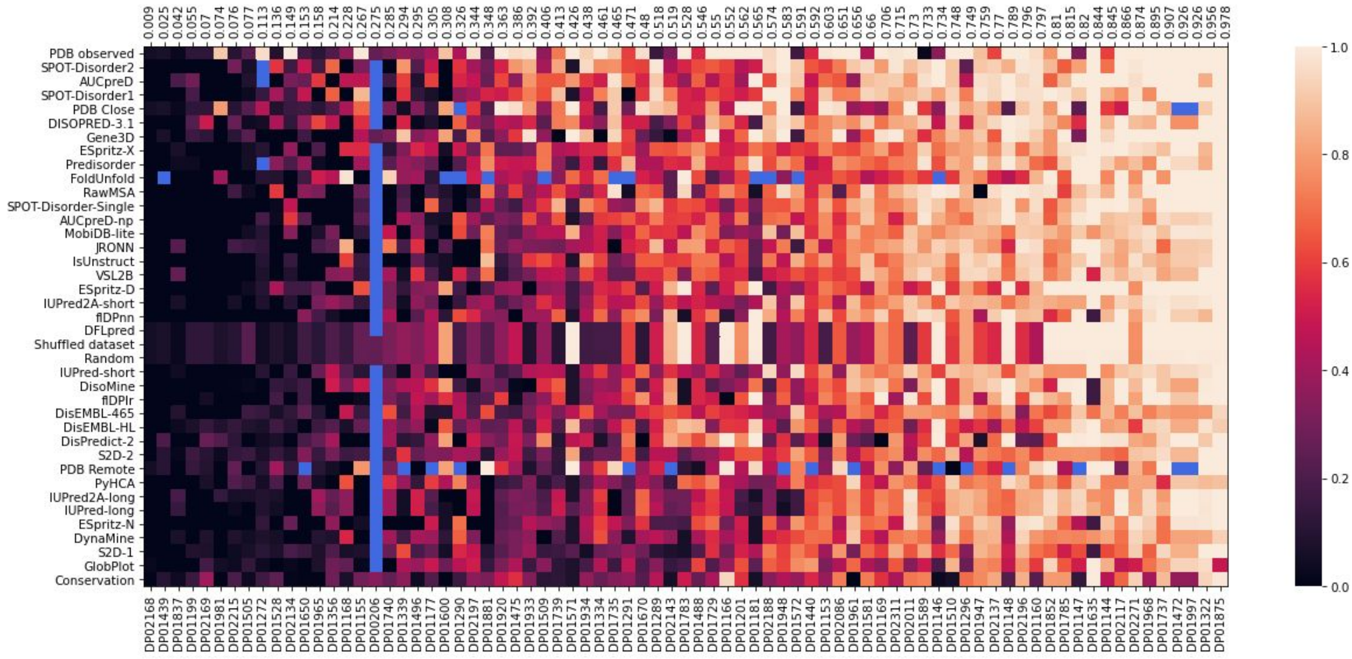

Supplementary Figure 48: F_{Max} per protein for prokaryotic proteins in the *DisProt-PDB* dataset. F_{Max} of each target (x-axis, bottom labels) from each predictor (y-axis). Targets are sorted by average F_{Max} (x-axis, top labels). Calculated on prokaryotic proteins of the *DisProt-PDB* dataset. Missing values are in blue.

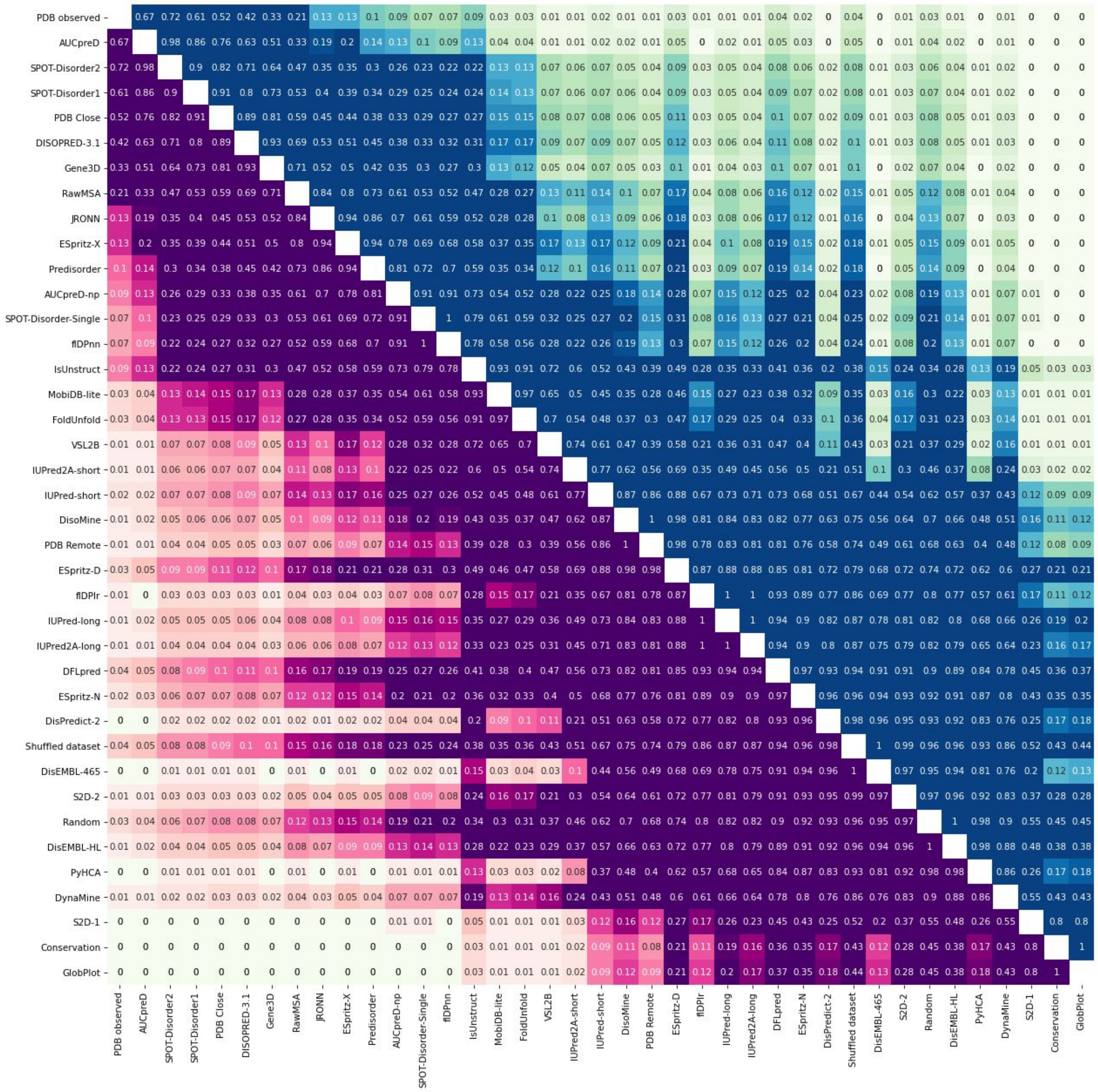

Supplementary Figure 49. Overall average ranking of all predictors and baselines for prokaryotic proteins in the *DisProt-PDB* dataset.

Heatmap of the T-test p-value associated to the statistical significance of the difference between ranking distribution of predictors. A ranking distribution for a predictor is the position of that predictor in its ranking for the following metrics: 'bac', 'f1s', 'fpr', 'mcc', 'ppv', 'tpr', 'tnr'. Metrics are calculated per target and with predictors threshold optimized by F1-Score.

Fully Disordered Proteins

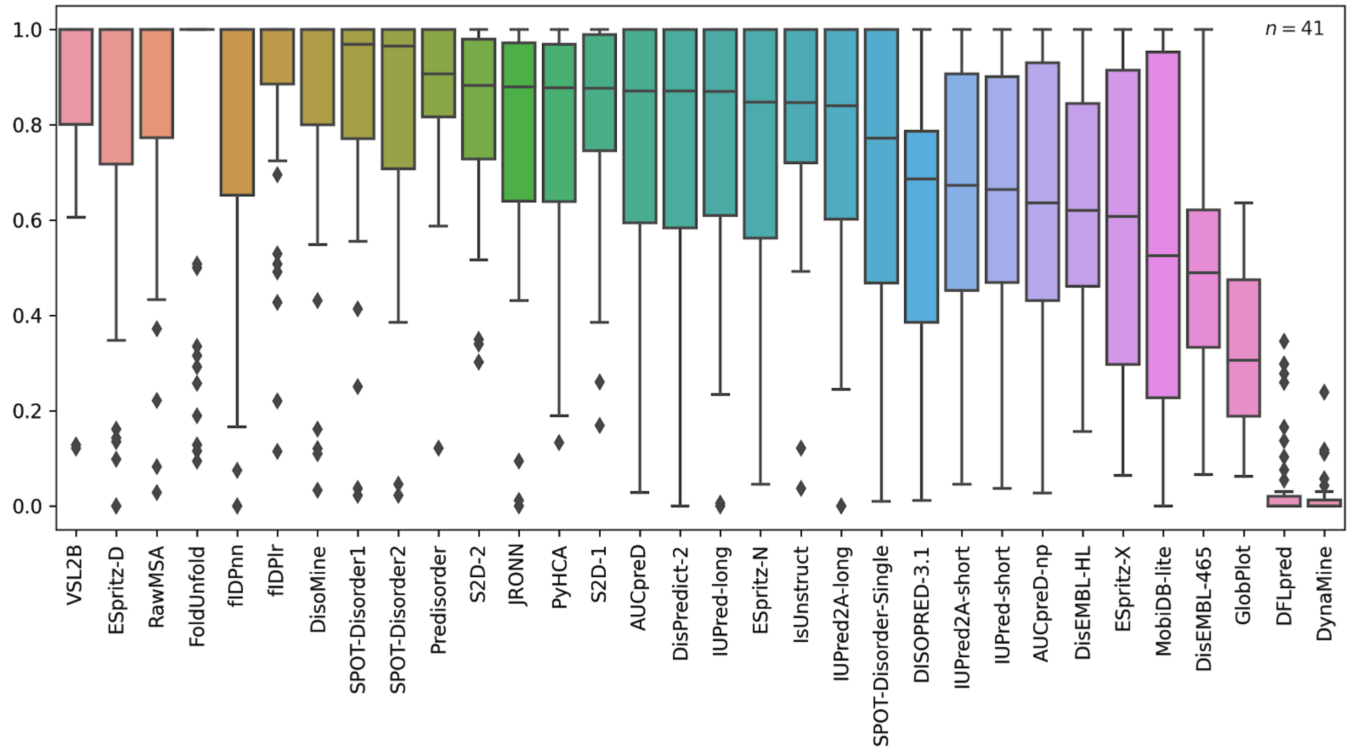

Supplementary Figure 50. Fraction of disordered residues in fully disordered proteins. Distribution of the fraction of disordered residues for the proteins with at least 95% of disordered residues in the *DisProt* dataset (n= 41 proteins) and in their predicted fraction of disordered residues for each method. Methods are sorted by their median. Boxplots are defined as follows: the middle value of the dataset is the median (Q2/50th Percentile). The box boundaries are the 1st quartile (Q1/25th Percentile) and 3rd quartile (Q3/75th Percentile) respectively; Maximum is Q3 + 1.5*(Q3-Q1) and Minimum is Q1 -1.5*(Q3-Q1).

Binding

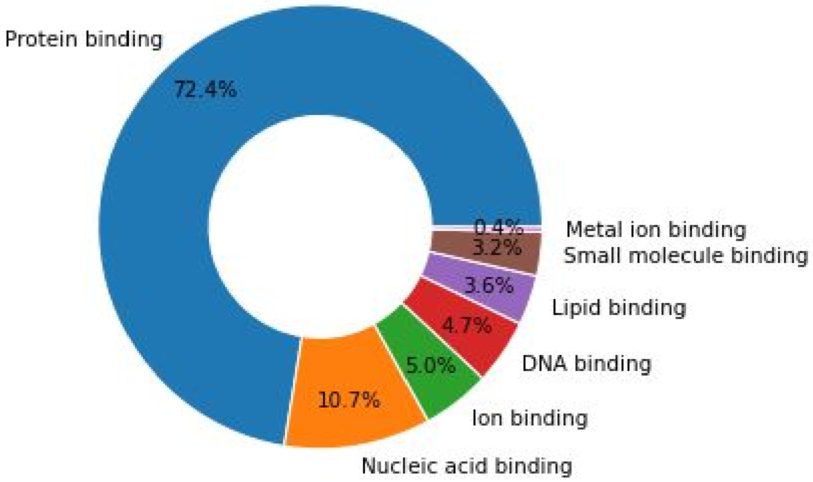

Supplementary Figure 51. Type of bindings in the *DisProt-Binding* dataset.
Abundance of the type of binding annotations in the *DisProt-Binding* datasets.

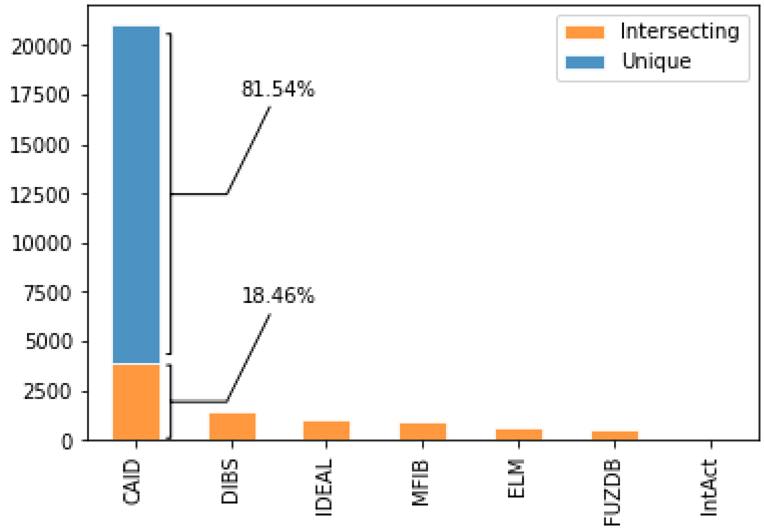

Supplementary Figure 52. Annotation overlap between resources annotating binding.
Number and fraction of residues annotated with binding in CAID dataset that are Unique to CAID dataset (blue) or present in other datasets (orange). n= 23,121 (ELM) + 22,487 (MFIB) + 13,975 (DIBS) + 9,650 (FUZDB) + 12,537 (IDEAL) + 1,233,543 (IntAct) + 21,027 (DisProt) = 1,336,340 binding residues.

DisProt-Binding dataset

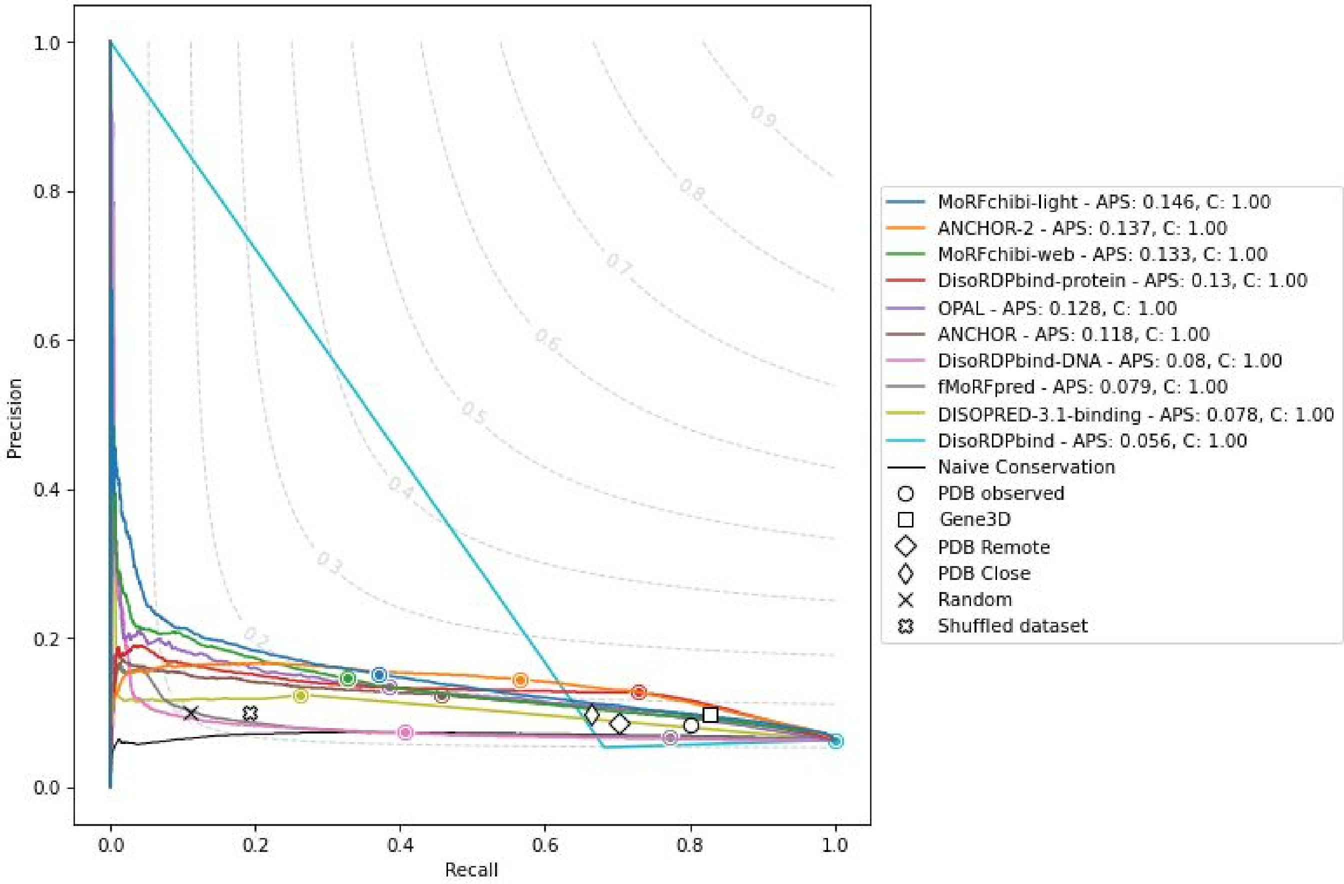

Supplementary Figure 53: Precision recall curve in the *DisProt-Binding* dataset.
Precision (y-axis) recall (x-axis) curves of the 10 best ranking methods. Ranking is based on their APS (average precision score) in the *DisProt-Binding* dataset.

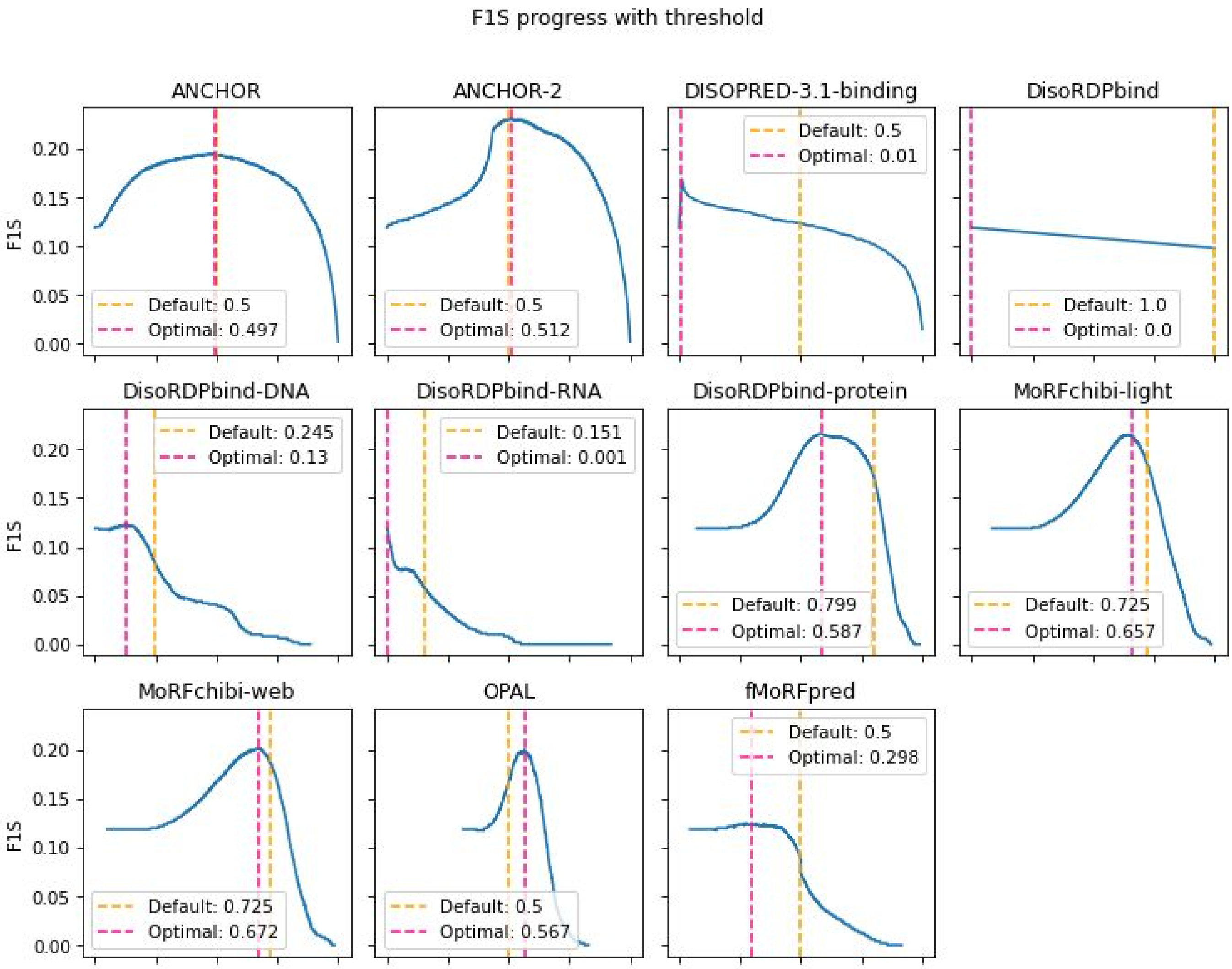

Supplementary Figure 54. F1-score progress with threshold in the *DisProt-Binding* dataset. F1-score progress (y-axis) with increasing threshold value (x-axis) for each predictor in the *DisProt-Binding* dataset.

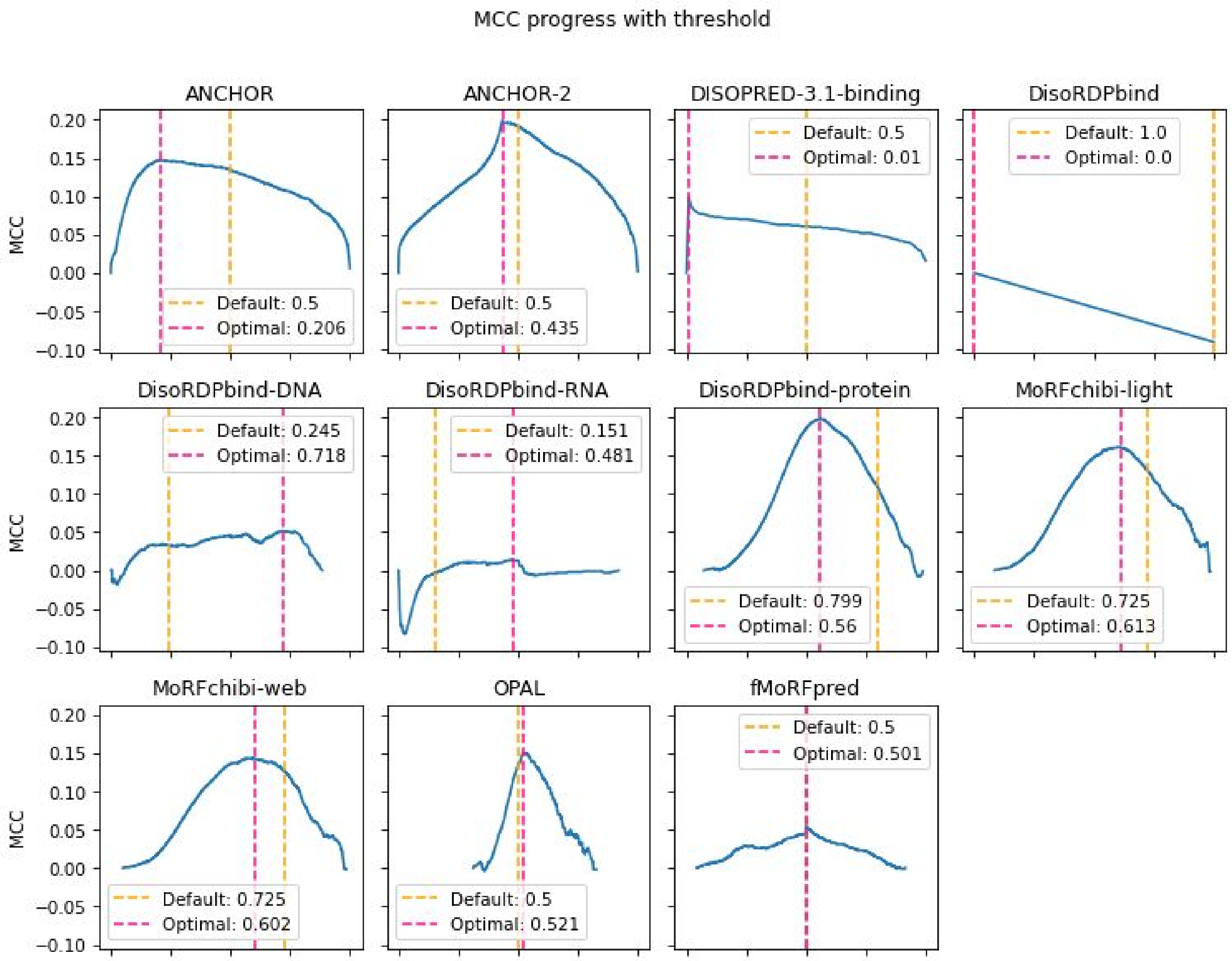

Supplementary Figure 55. MCC progress with threshold in the *DisProt-Binding* dataset.
MCC progress (y-axis) with increasing threshold value (x-axis) for each predictor in the *DisProt-binding* dataset.

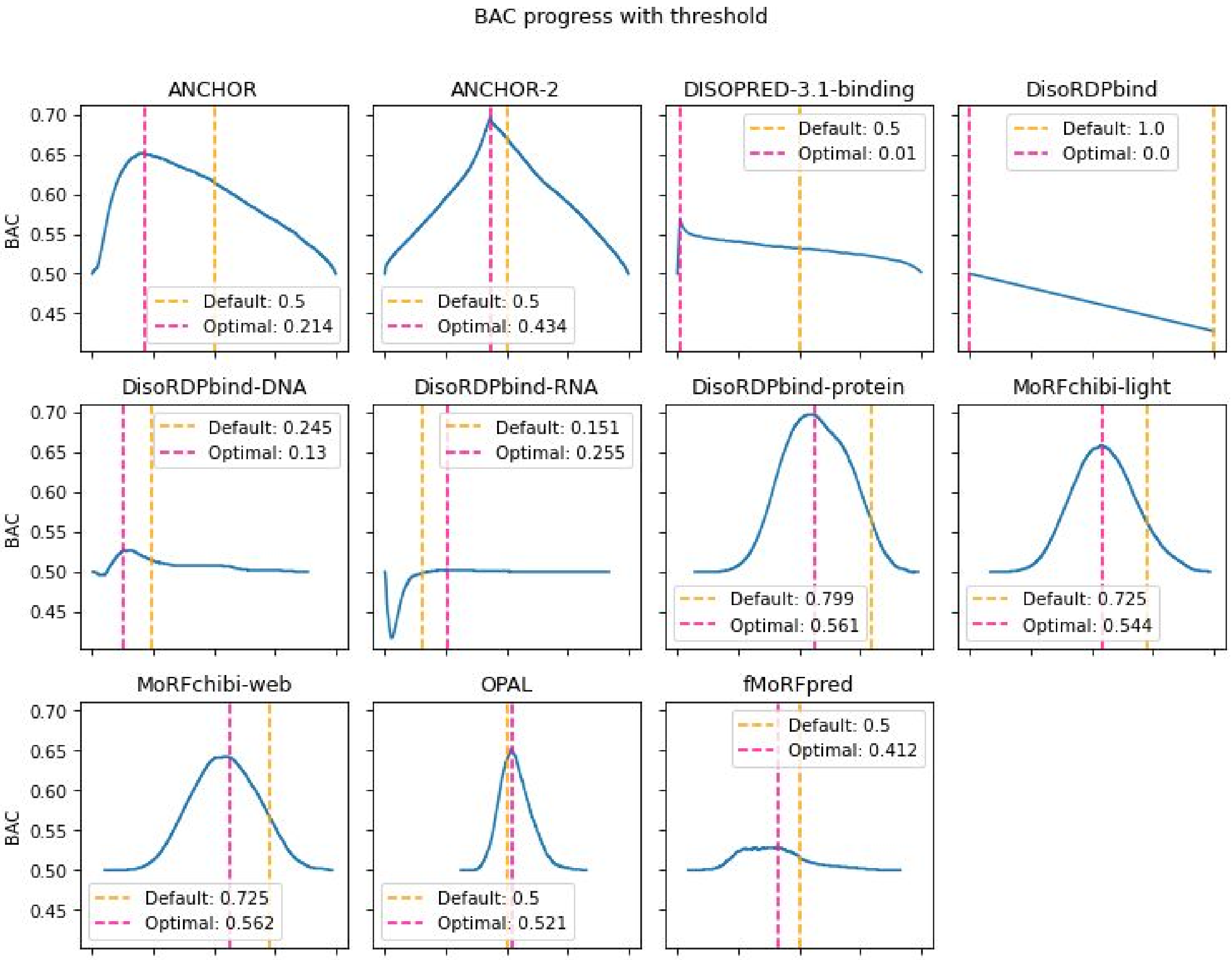

Supplementary Figure 56. Balanced accuracy progress with threshold in the *DisProt-Binding* dataset.

Balanced accuracy progress (y-axis) with increasing threshold value (x-axis) for each predictor in the *DisProt-binding* dataset.

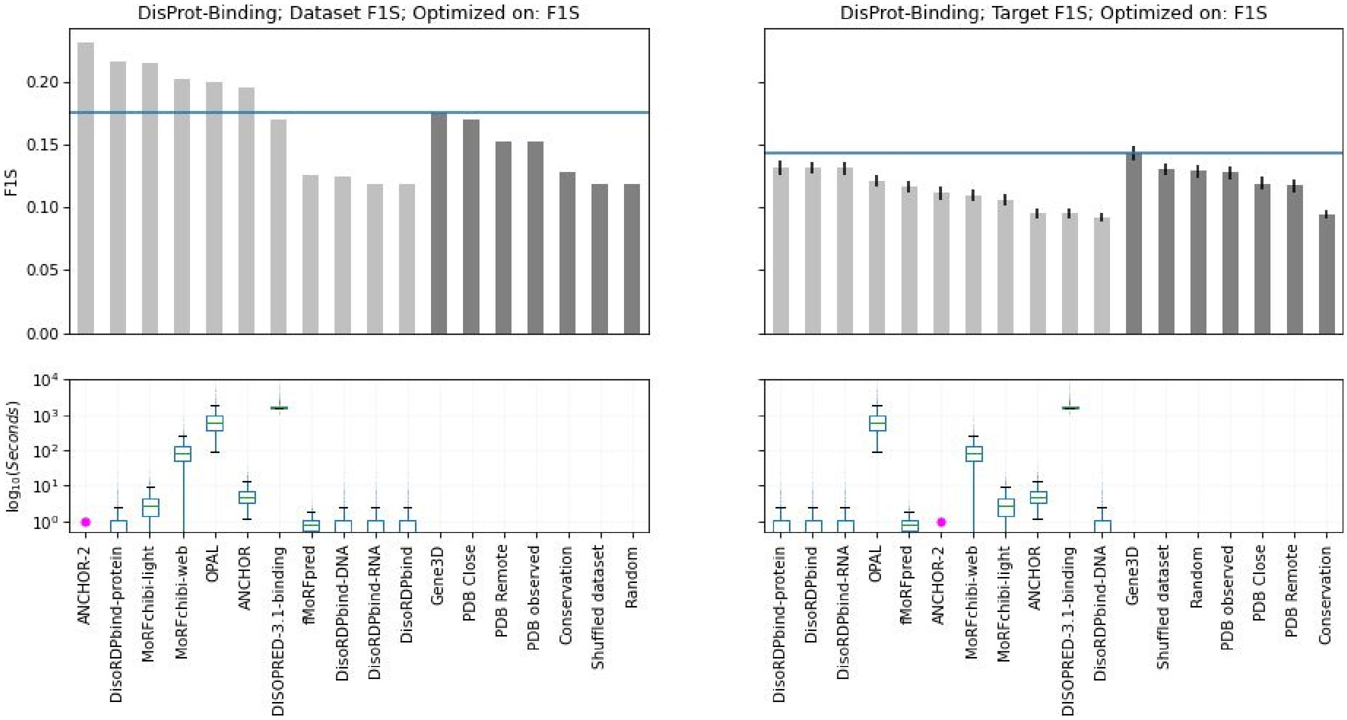

Supplementary Figure 57: F_{Max} in the *DisProt-Binding* dataset.

F_{Max} calculated on the whole dataset with confidence intervals as error bars (left) and averaged over proteins with Standard-Error as error bars (right). Calculated on *DisProt-Binding* dataset (n= 646 proteins). Boxplots are defined as follows: the middle value of the dataset is the median (Q2/50th Percentile). The box boundaries are the 1st quartile (Q1/25th Percentile) and 3rd quartile (Q3/75th Percentile) respectively; Maximum is Q3 + 1.5*(Q3-Q1) and Minimum is Q1 -1.5*(Q3-Q1). Outliers are hidden for clarity. Magenta dots indicate that the whole distribution of execution-times is lower than 1 second.

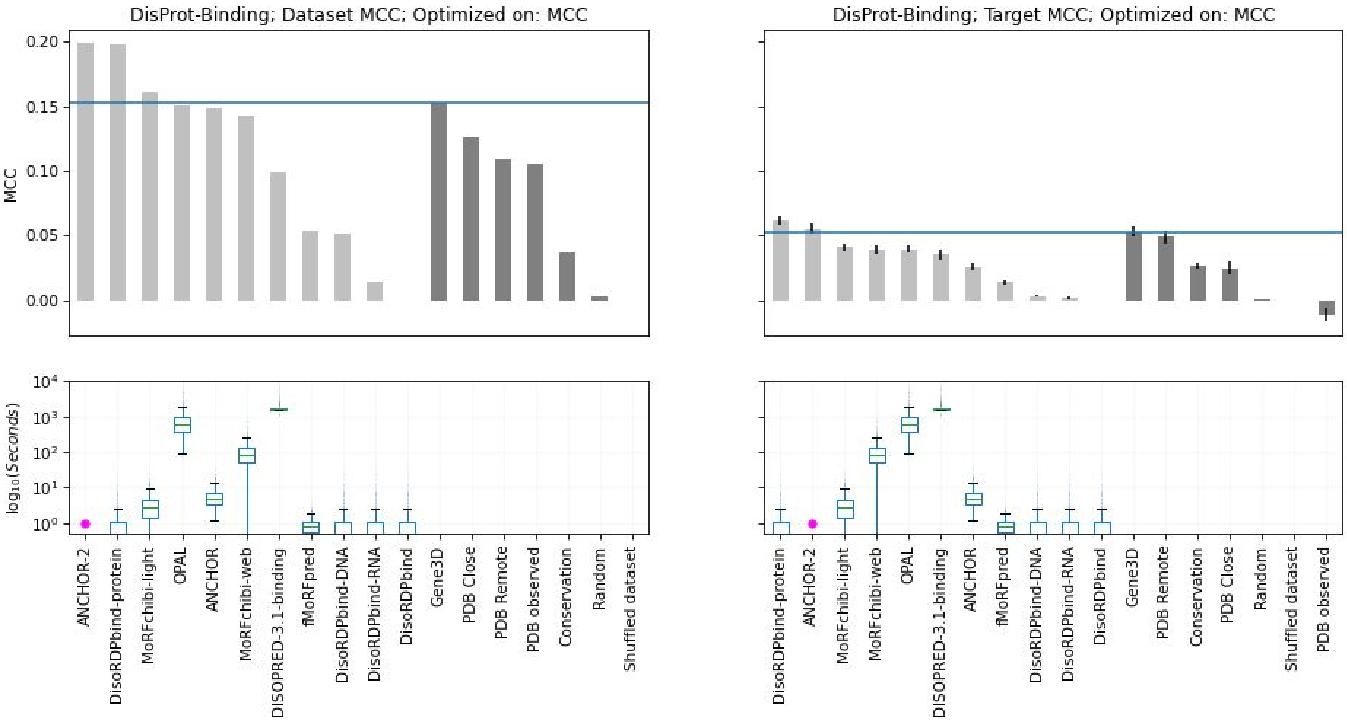

Supplementary Figure 58: MCC in the *DisProt-Binding* dataset.
MCC calculated on the whole dataset with confidence intervals as error bars (left) and averaged over proteins with Standard-Error as error bars (right). Calculated on *DisProt-Binding* dataset (n= 646 proteins). Predictors threshold is optimized on MCC. Boxplots are defined as follows: the middle value of the dataset is the median (Q2/50th Percentile). The box boundaries are the 1st quartile (Q1/25th Percentile) and 3rd quartile (Q3/75th Percentile) respectively; Maximum is Q3 + 1.5*(Q3-Q1) and Minimum is Q1 -1.5*(Q3-Q1). Outliers are hidden for clarity. Magenta dots indicate that the whole distribution of execution-times is lower than 1 second.

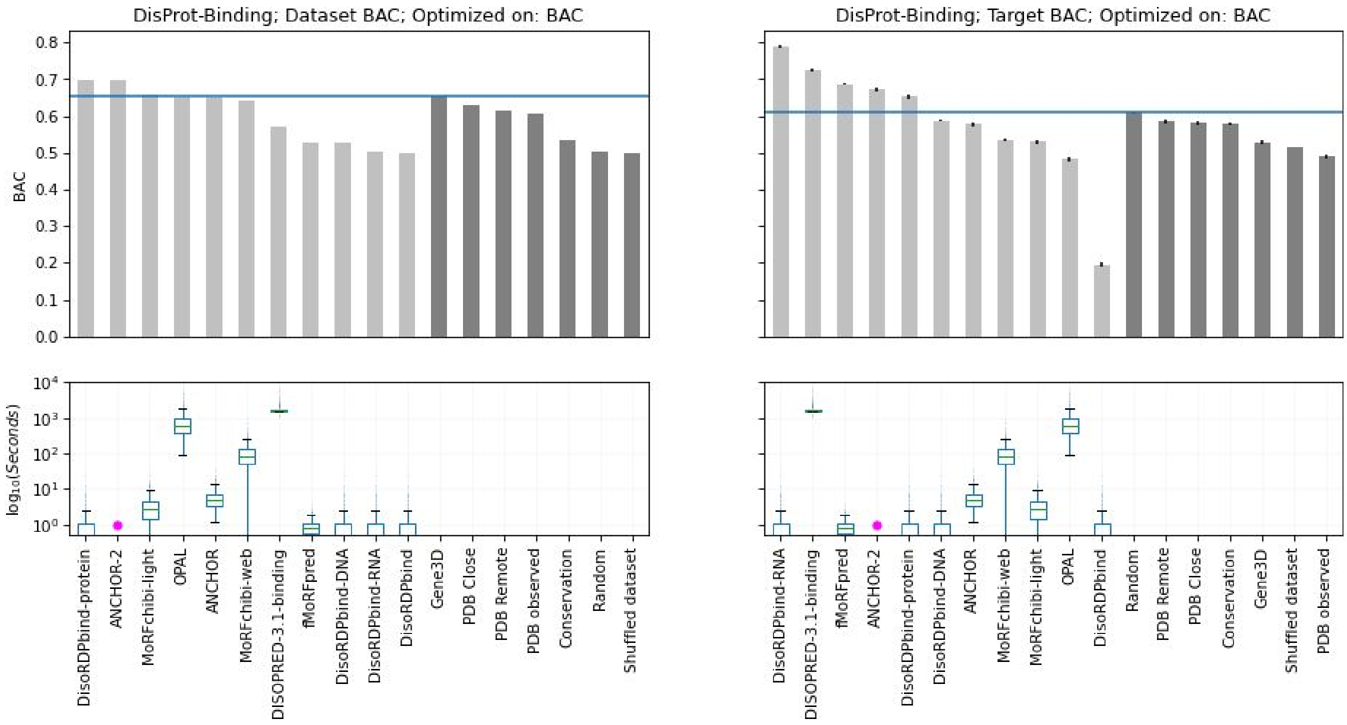

Supplementary Figure 59: Balanced accuracy in the *DisProt-Binding* dataset.
Balanced accuracy calculated on the whole dataset with confidence intervals as error bars (left) and averaged over proteins with Standard-Error as error bars (right). Calculated on *DisProt-Binding* dataset (n= 646 proteins). Predictors threshold is optimized on Balanced accuracy. Boxplots are defined as follows: the middle value of the dataset is the median (Q2/50th Percentile). The box boundaries are the 1st quartile (Q1/25th Percentile) and 3rd quartile (Q3/75th Percentile) respectively; Maximum is Q3 + 1.5*(Q3-Q1) and Minimum is Q1 -1.5*(Q3-Q1). Outliers are hidden for clarity. Magenta dots indicate that the whole distribution of execution-times is lower than 1 second.

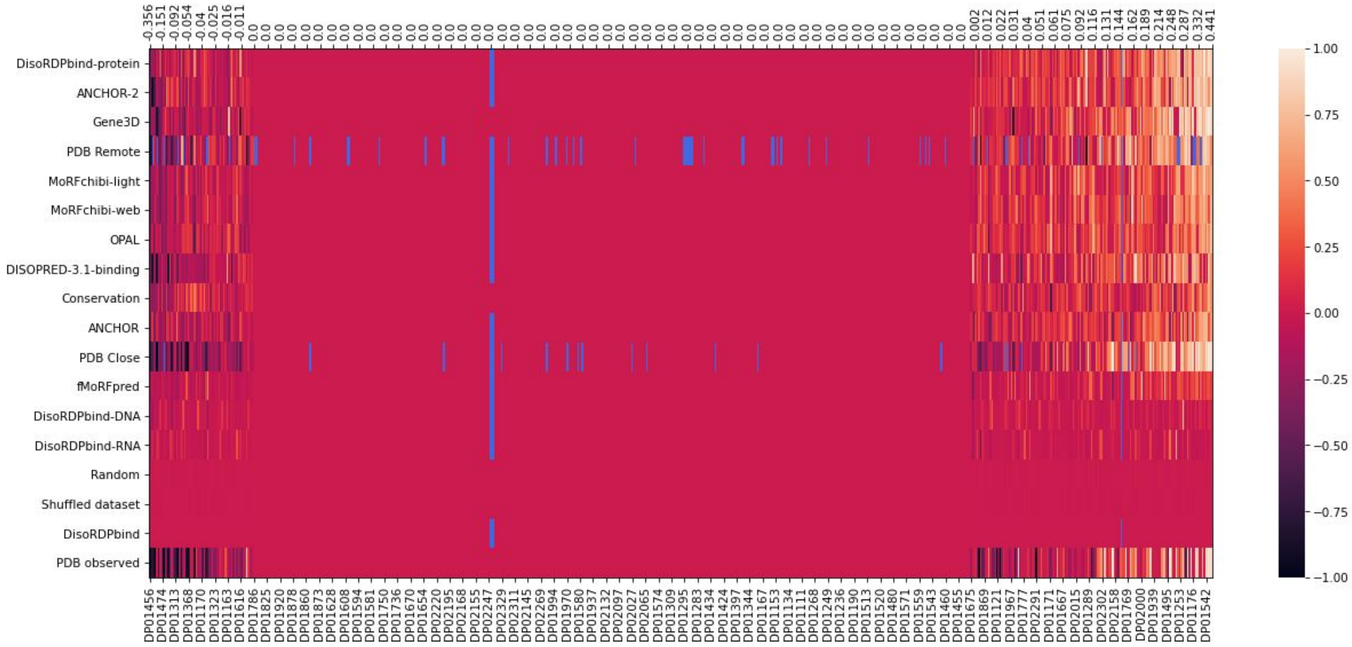

Supplementary Figure 60: MCC per protein in the *DisProt-Binding* dataset.

MCC of each target (x-axis, bottom labels, not all labels are visible) from each predictor (y-axis). Targets are sorted by average MCC (x-axis, top labels). Calculated on *DisProt-Binding*. Predictors threshold is optimized on MCC. Missing values are in blue.

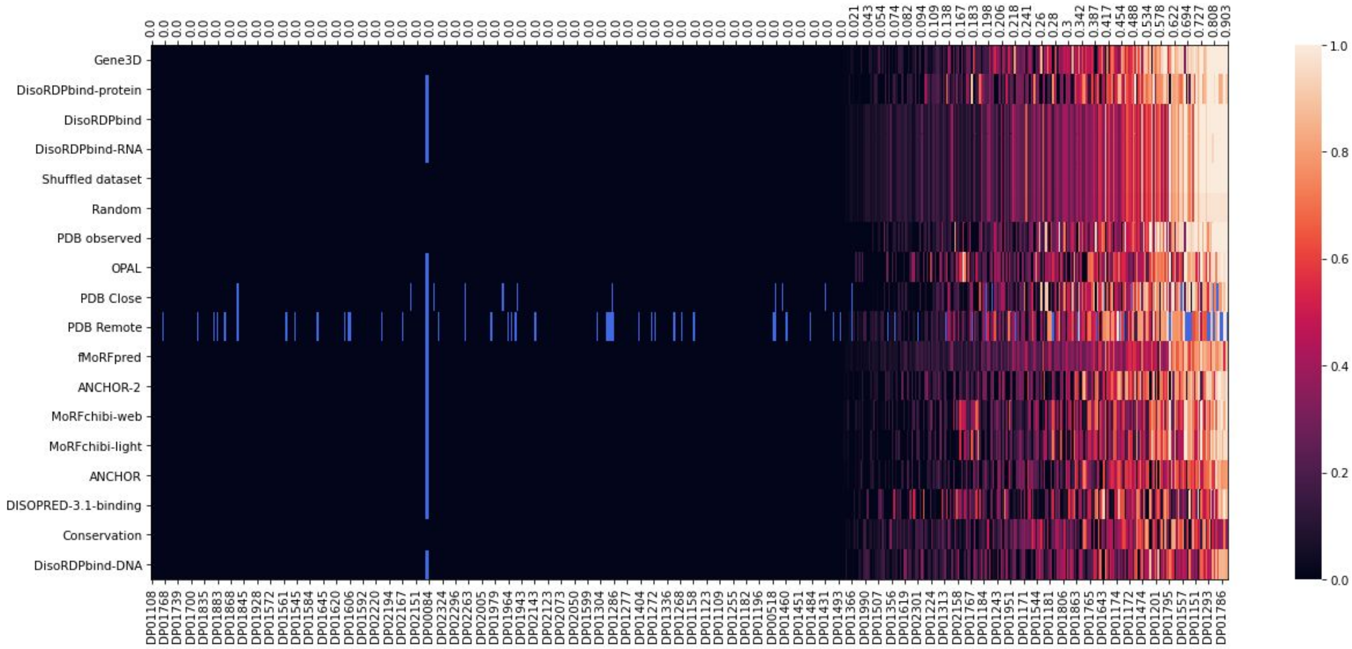

Supplementary Figure 61: F_{Max} per protein in the *DisProt-Binding* dataset.

F_{Max} of each target (x-axis, bottom labels, not all labels are visible) from each predictor (y-axis). Targets are sorted by average F_{Max} (x-axis, top labels). Calculated on *DisProt-Binding* dataset. Missing values are in blue.

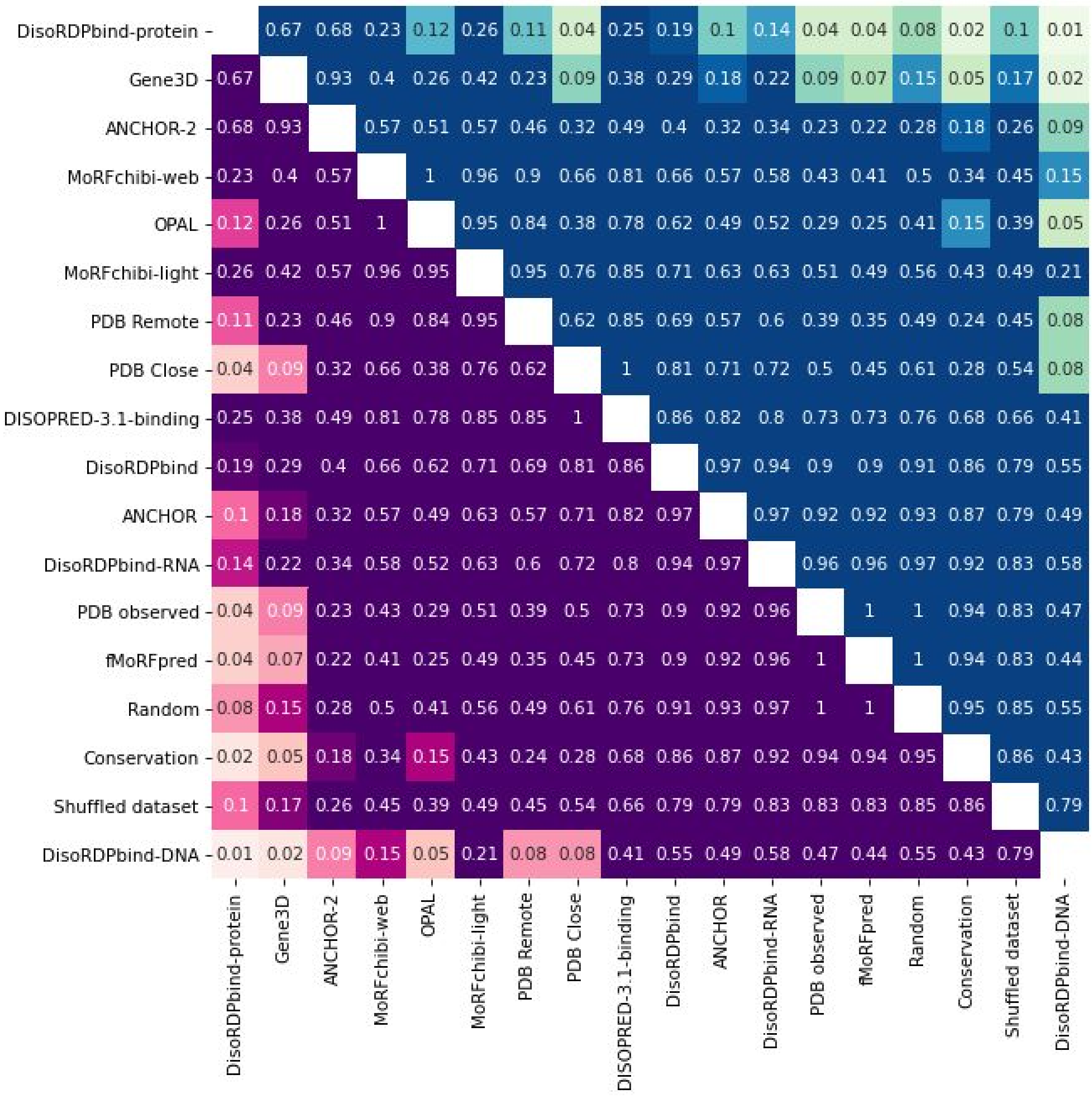

Supplementary Figure 62. Overall average ranking of all predictors and baselines.
Heatmap of the T-test p-value associated to the statistical significance of the difference between ranking distribution of predictors. A ranking distribution for a predictor is the position of that predictor in its ranking for each metric. Metrics used are: bac, f1s, fpr, mcc, ppv, tpr, tnr; they are calculated with predictors threshold optimized by F1-Score.

Mammals

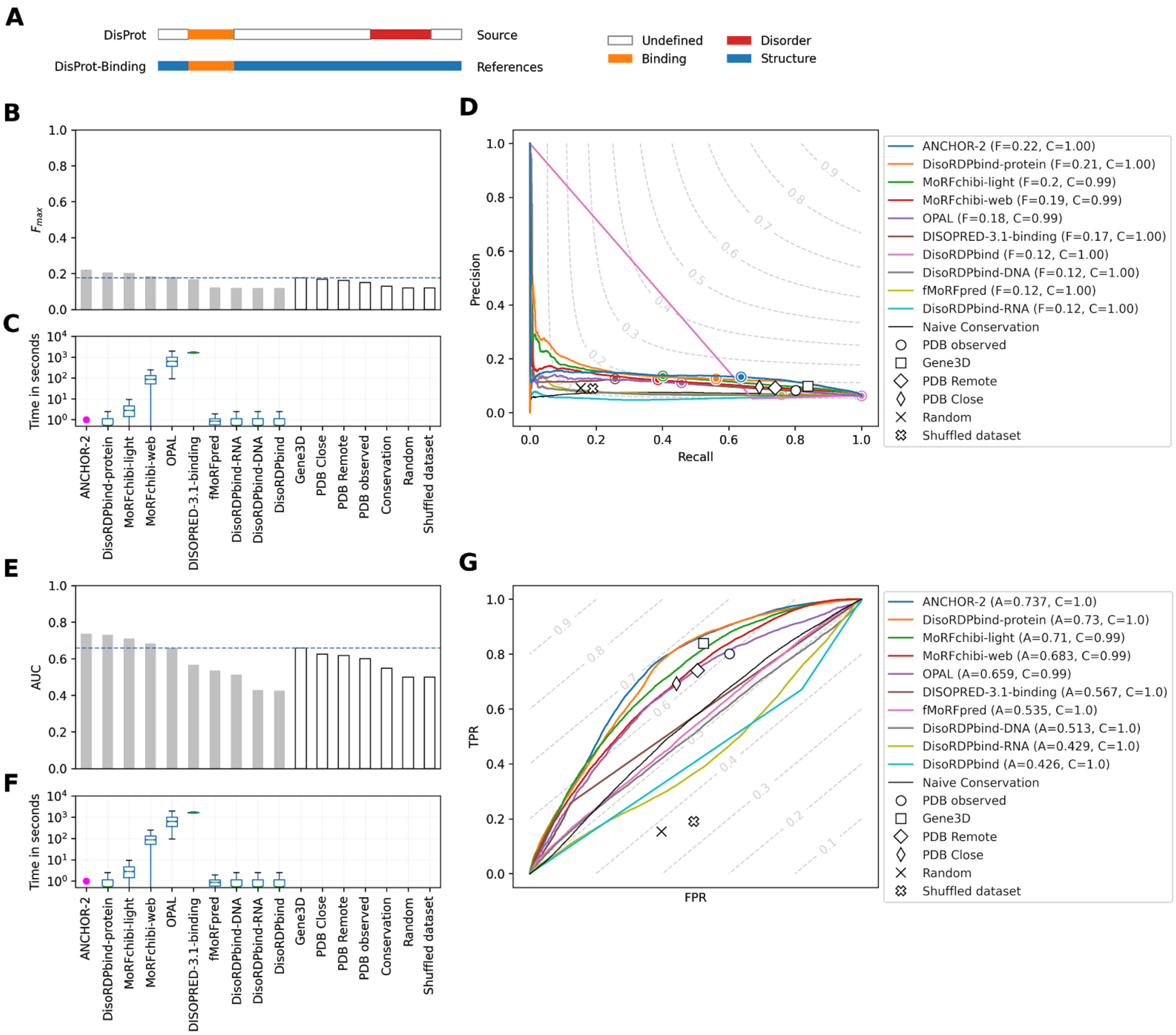

Supplementary Figure 63. Prediction success and CPU times for the ten top-ranking disorder predictors for mammalian proteins in the *DisProt-Binding* dataset.

Prediction success and CPU times for the ten top-ranking disorder predictors for mammalian proteins in the *DisProt-Binding* dataset (n= 368 proteins). Reference used (*DisProt-Binding*) in the analysis and how it is obtained (panel A). Performance of predictors expressed as maximum F1-Score across all thresholds (F_{max}) (panel B) and AUC (panel E) for the top ten best ranking methods (light gray) and baselines (white) and the distribution of execution time per-target (panels C, F) using *DisProt-Binding* dataset. The horizontal line in panels B, E indicates the F_{max} and AUC of the best baseline, respectively. Precision-Recall (panel D) and ROC curves (panel G) of ten top-ranking methods and baselines using *DisProt-Binding* dataset, with level curves of the F1-Score and Balanced accuracy, respectively. Boxplots in panels **C, F** are defined as follows: the middle value of the dataset is the median (Q2/50th Percentile). The box boundaries are the 1st quartile (Q1/25th Percentile) and 3rd quartile (Q3/75th Percentile) respectively; Maximum is Q3 + 1.5*(Q3-Q1) and Minimum is Q1 -1.5*(Q3-Q1). Outliers are hidden for clarity. Magenta dots on panels C, F indicate that the whole distribution of execution-times is lower than 1 second.

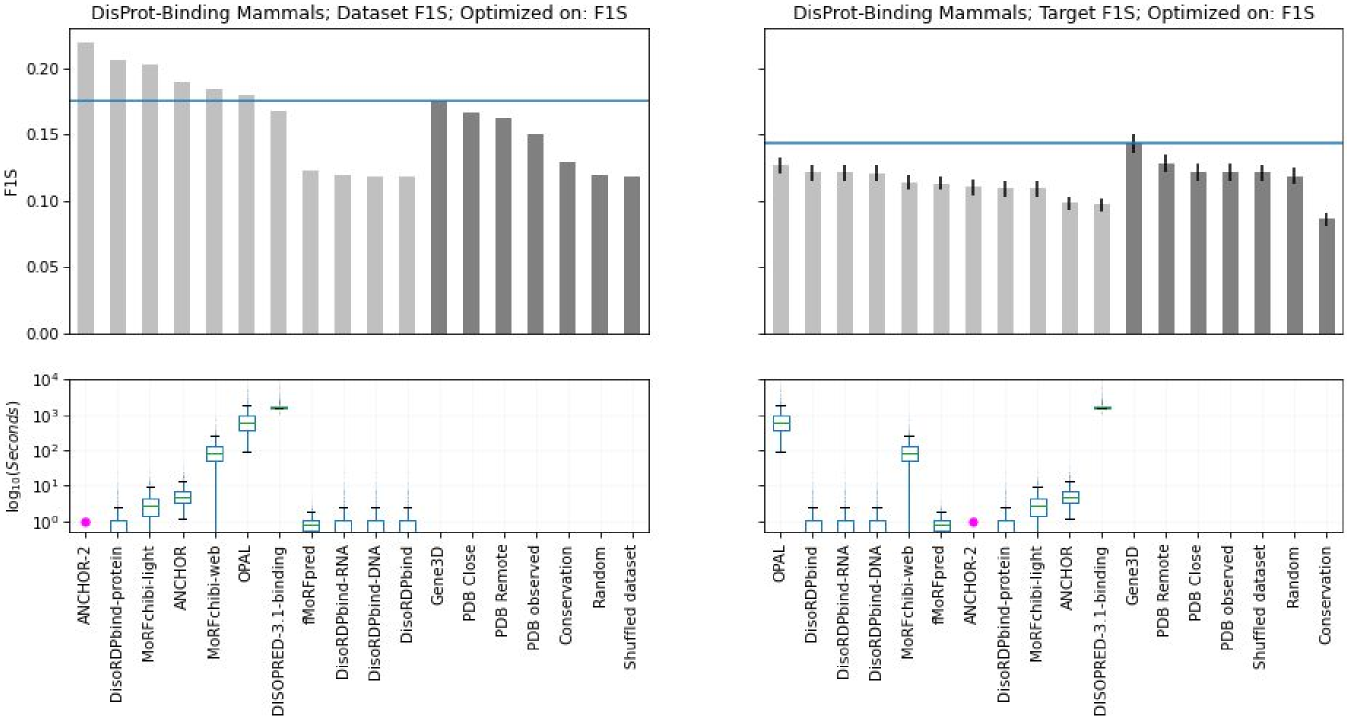

Supplementary Figure 64: F_{Max} for mammalian proteins in the *DisProt-Binding* proteins.
F_{Max} calculated on the whole dataset with confidence intervals as error bars (left) and averaged over proteins with Standard-Error as error bars (right). Calculated on mammalian proteins of the *DisProt-Binding* dataset (n= 368 proteins). Boxplots are defined as follows: the middle value of the dataset is the median (Q2/50th Percentile). The box boundaries are the 1st quartile (Q1/25th Percentile) and 3rd quartile (Q3/75th Percentile) respectively; Maximum is Q3 + 1.5*(Q3-Q1) and Minimum is Q1 -1.5*(Q3-Q1). Outliers are hidden for clarity. Magenta dots indicate that the whole distribution of execution-times is lower than 1 second.

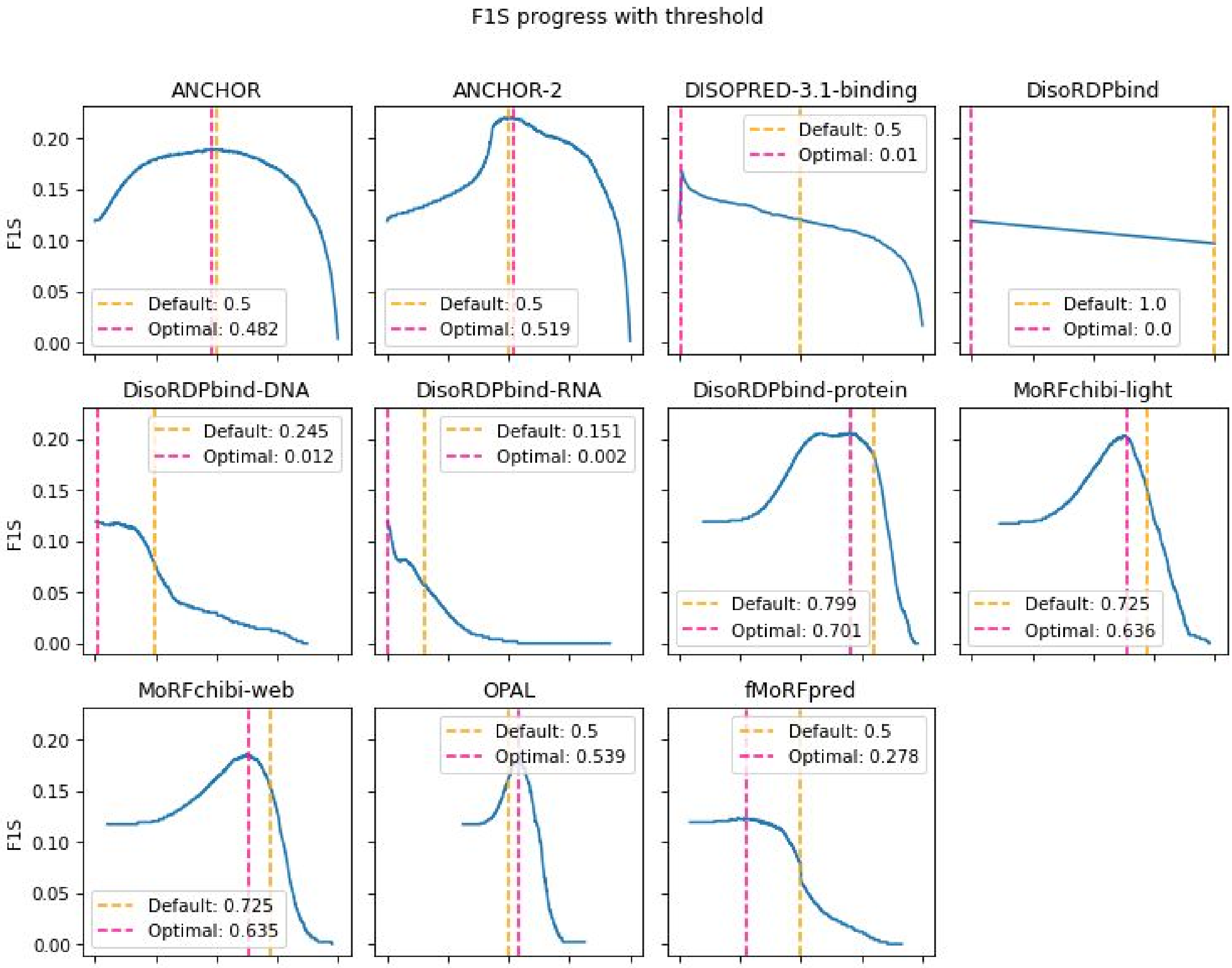

Supplementary Figure 65. F1-Score progress with threshold for mammalian proteins in the *DisProt-Binding* dataset.

F1-score progress (y-axis) with increasing threshold value (x-axis) for each predictor calculated on mammalian proteins on the *DisProt-Binding* dataset.

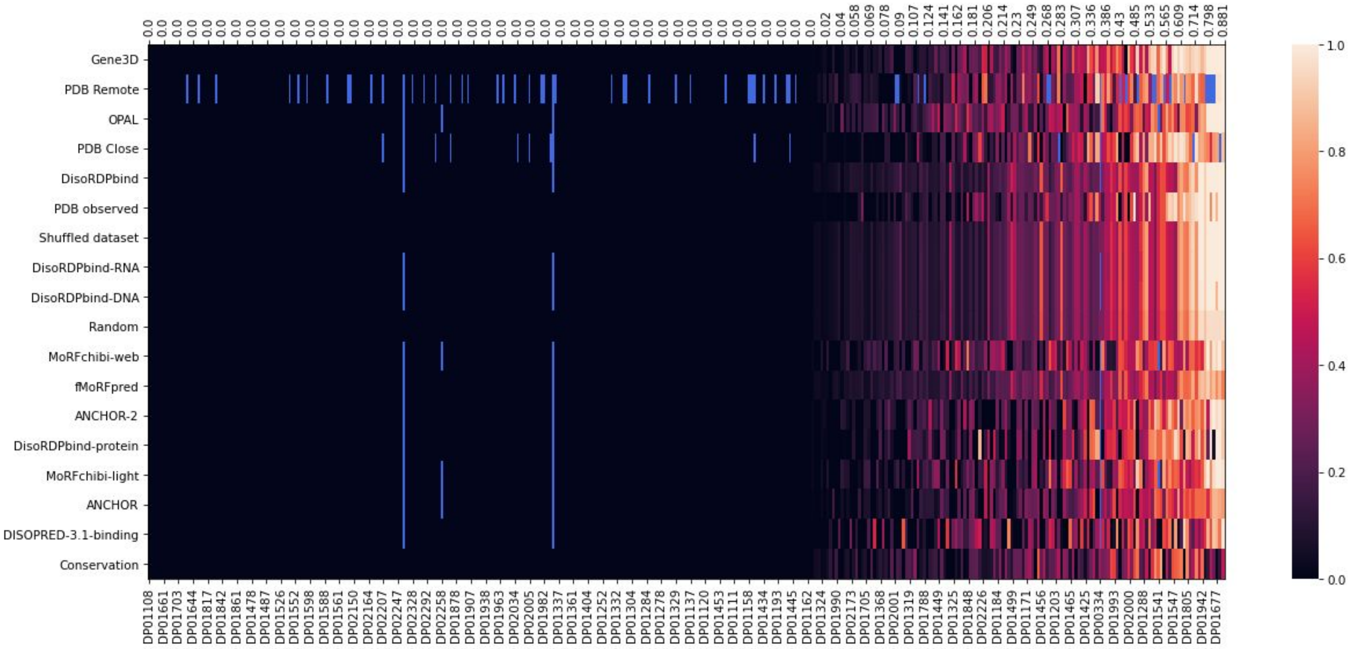

Supplementary Figure 66: F_{Max} per proteins for mammalian proteins in the *DisProt-Binding* dataset.

F_{Max} of each target (x-axis, bottom labels, not all labels are visible) from each predictor (y-axis). Targets are sorted by average F_{Max} (x-axis, top labels). Calculated on mammalian proteins of the *DisProt-Binding* dataset. Missing values are in blue.

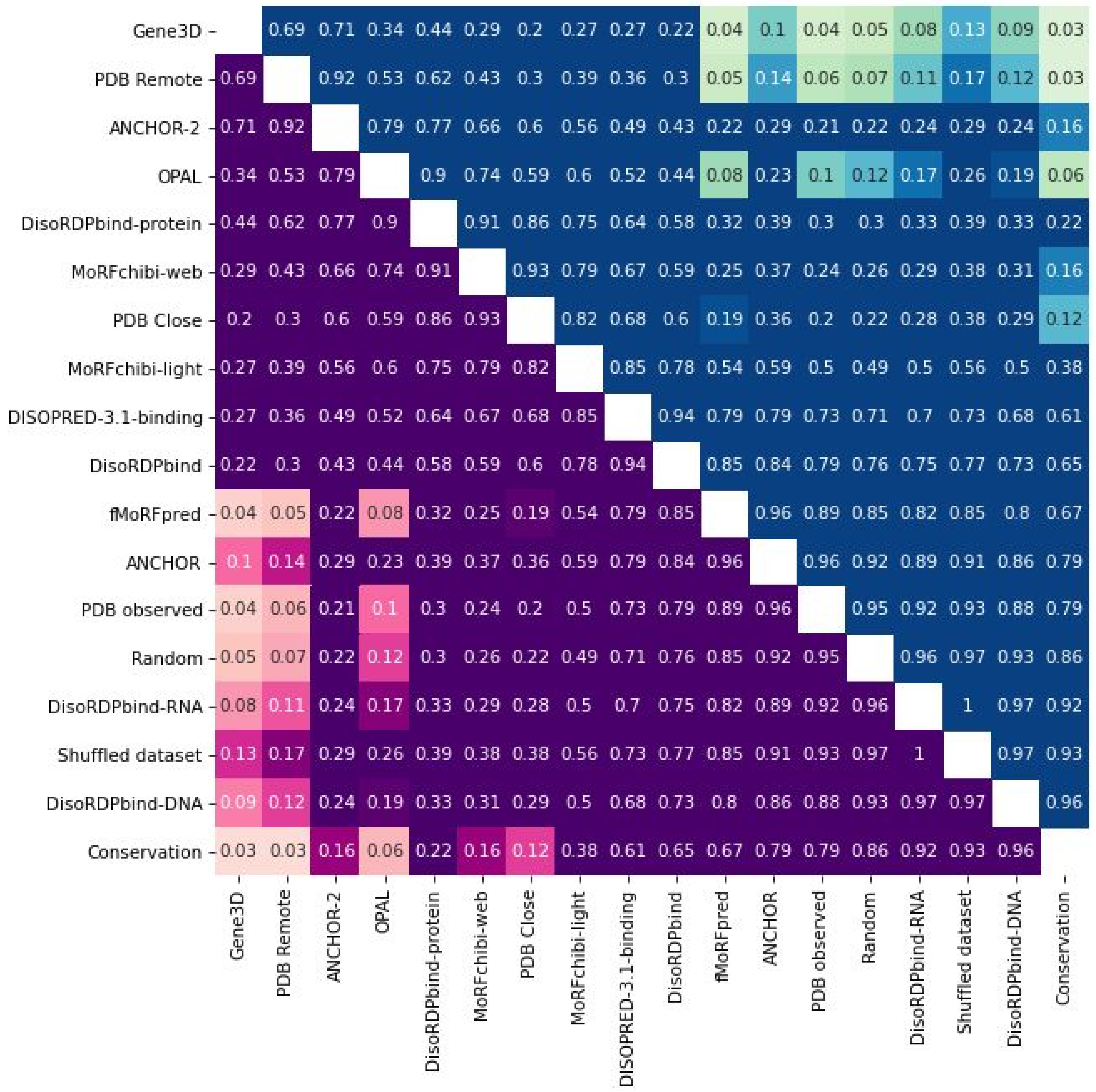

Supplementary Figure 67. Overall average ranking of all predictors and baselines for mammalian proteins in the *DisProt-Binding* dataset.

Heatmap of the T-test p-value associated to the statistical significance of the difference between ranking distribution of predictors. A ranking distribution for a predictor is the position of that predictor in its ranking for the following metrics: 'bac', 'f1s', 'fpr', 'mcc', 'ppv', 'tpr', 'tnr'. Metrics are calculated per target and with predictors threshold optimized by F1-Score.

Prokaryotes

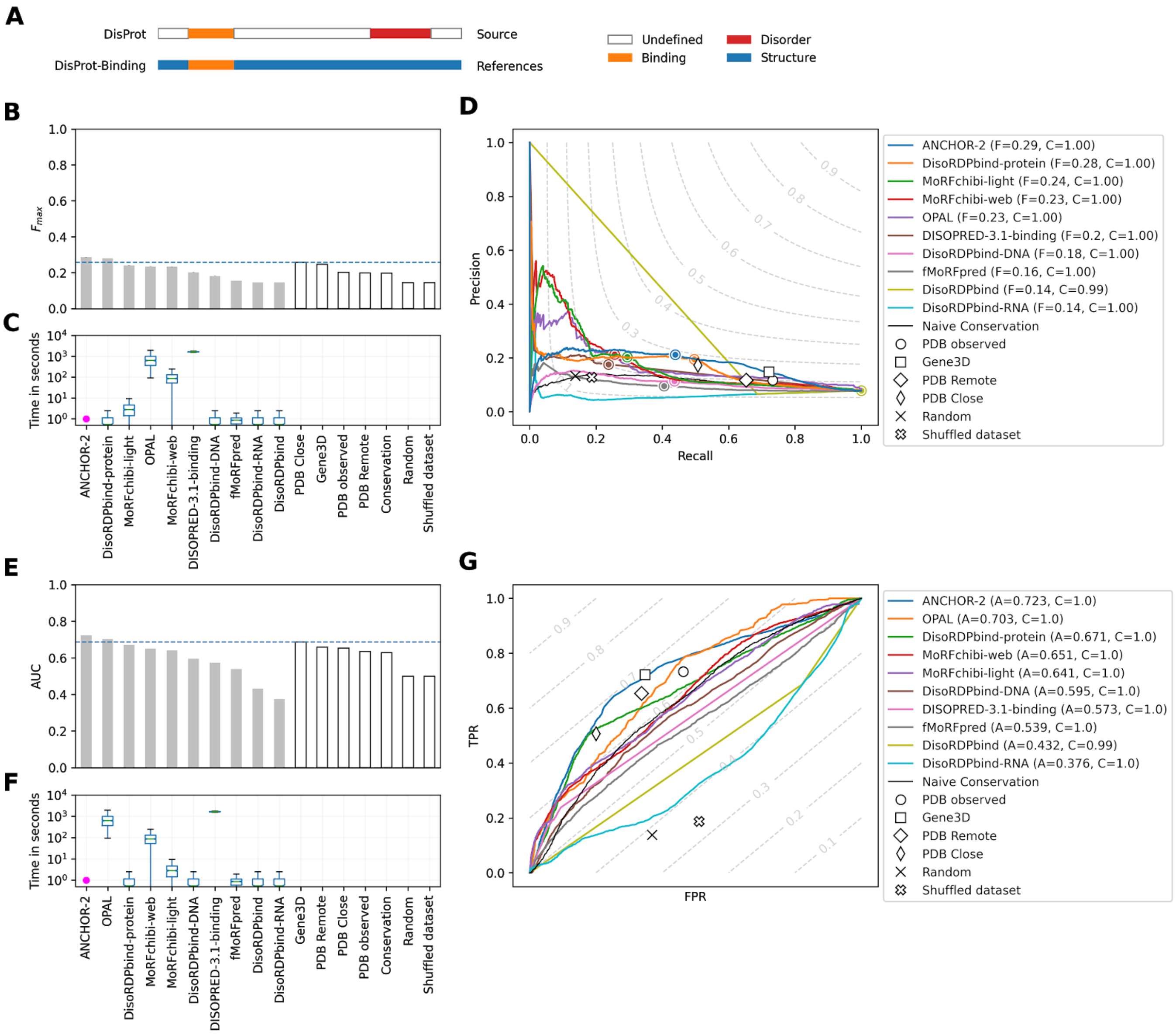

Supplementary Figure 68. Prediction success and CPU times for the ten top-ranking disorder predictors for prokaryotic proteins in the *DisProt-Binding* dataset.
Prediction success and CPU times for the ten top-ranking disorder predictors for prokaryotic proteins in the *DisProt-Binding* dataset (n= 77 proteins). Reference used (*DisProt-Binding*) in the analysis and how it is obtained (panel **A**). Performance of predictors expressed as maximum F1-Score across all thresholds (F_{max}) (panel **B**) and AUC (panel **E**) for the top ten best ranking methods (light gray) and baselines (white) and the distribution of execution time per-target (panels **C, F**) using *DisProt-Binding* dataset. The horizontal line in panels B, E indicates the F_{max} and AUC of the best baseline, respectively. Precision-Recall (panel **D**) and ROC curves (panel **G**) of ten top-ranking methods and baselines using *DisProt-Binding* dataset, with level curves of the F1-Score and Balanced accuracy, respectively. Boxplots in panels **C, F** are defined as follows: the middle value of the dataset is the median (Q2/50th Percentile). The box boundaries are the 1st quartile (Q1/25th Percentile) and 3rd quartile (Q3/75th Percentile) respectively; Maximum is Q3 + 1.5*(Q3-Q1) and Minimum is Q1 -1.5*(Q3-Q1). Outliers are hidden for clarity. Magenta dots on panels C, F indicate that the whole distribution of execution-times is lower than 1 second.

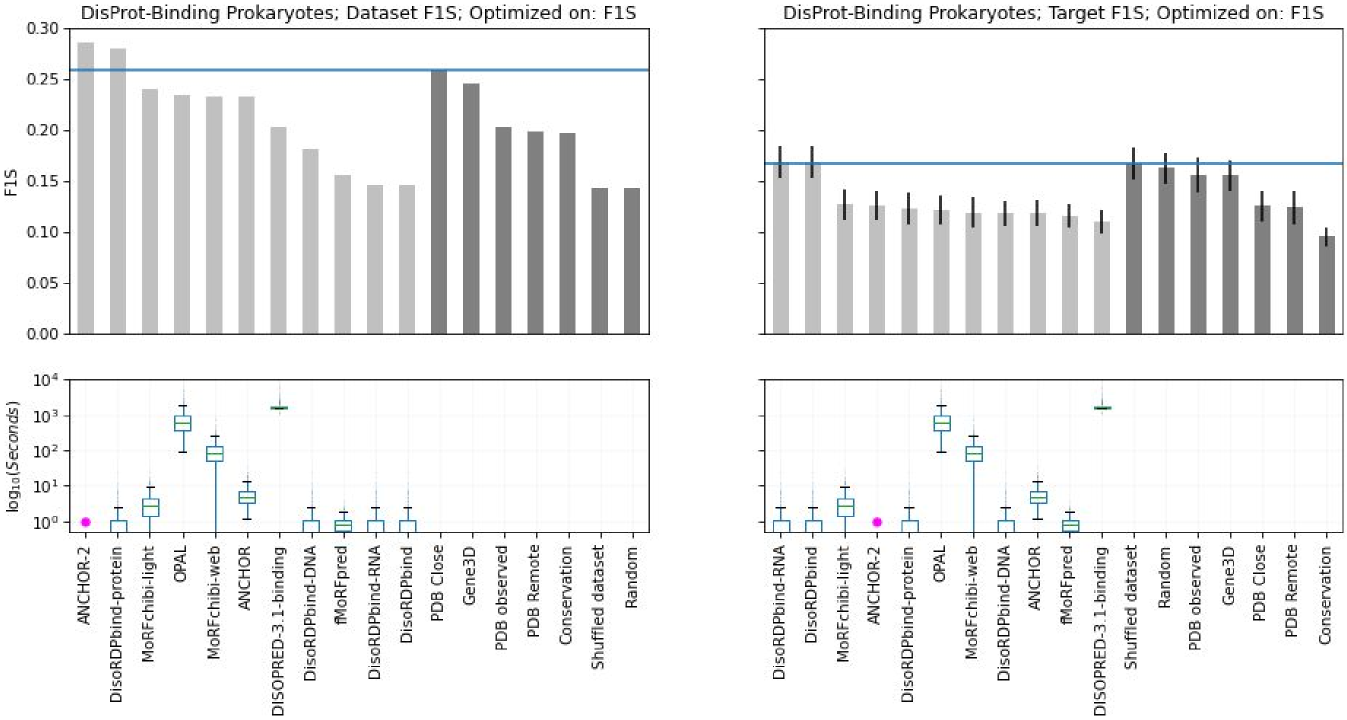

Supplementary Figure 69: F_{Max} for prokaryotic proteins in the *DisProt-Binding* dataset.
F_{Max} calculated on the whole dataset with confidence intervals as error bars (left) and averaged over proteins with Standard-Error as error bars (right). Calculated on prokaryotic proteins of the *DisProt-Binding* dataset (n= 77 proteins). Boxplots are defined as follows: the middle value of the dataset is the median (Q2/50th Percentile). The box boundaries are the 1st quartile (Q1/25th Percentile) and 3rd quartile (Q3/75th Percentile) respectively; Maximum is Q3 + 1.5*(Q3-Q1) and Minimum is Q1 -1.5*(Q3-Q1). Outliers are hidden for clarity. Magenta dots indicate that the whole distribution of execution-times is lower than 1 second.

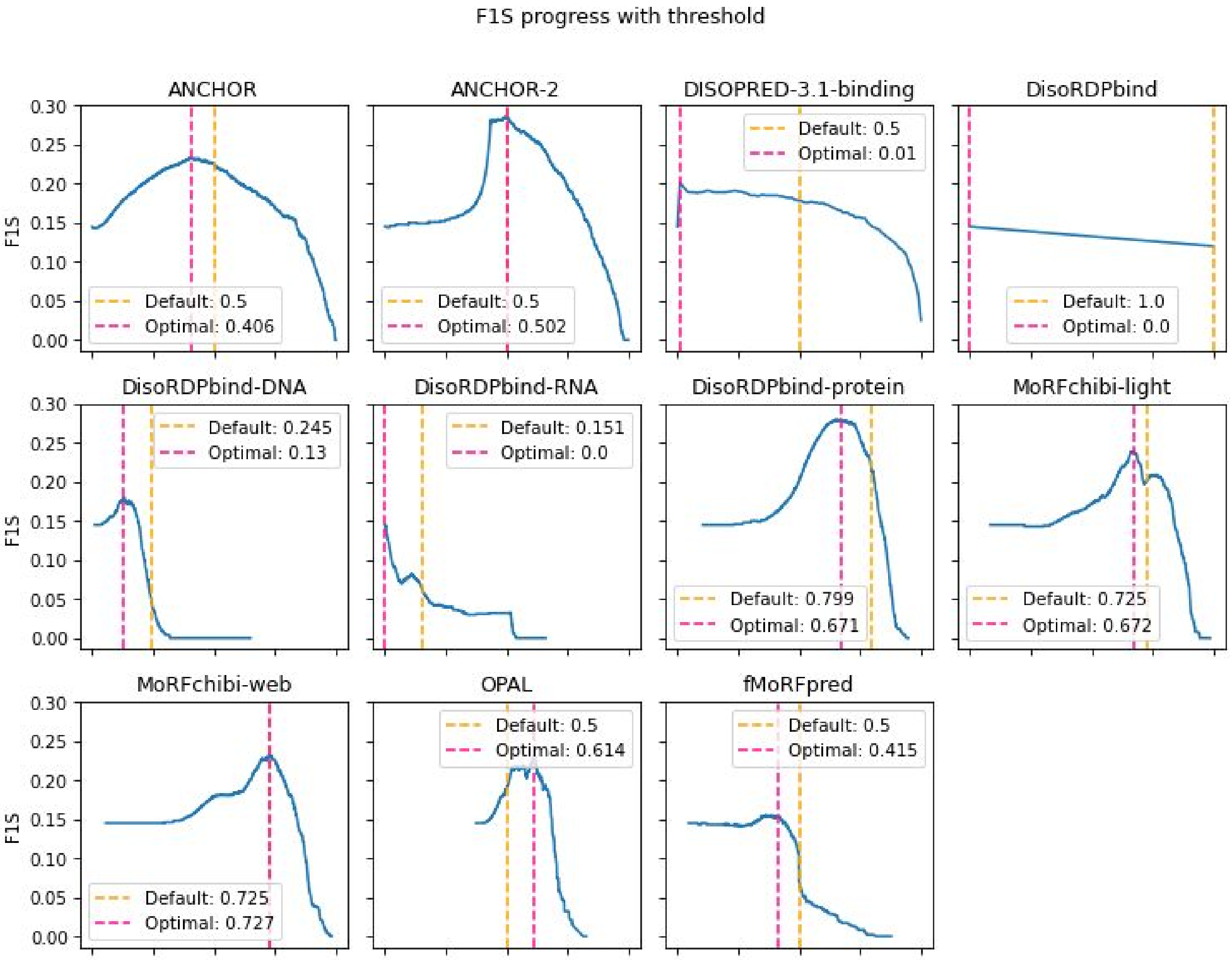

Supplementary Figure 70. F1-Score progress with threshold for prokaryotic proteins in the *DisProt-Binding* dataset.

F1-score progress (y-axis) with increasing threshold value (x-axis) for each predictor calculated on prokaryotic proteins on the *DisProt-Binding* dataset.

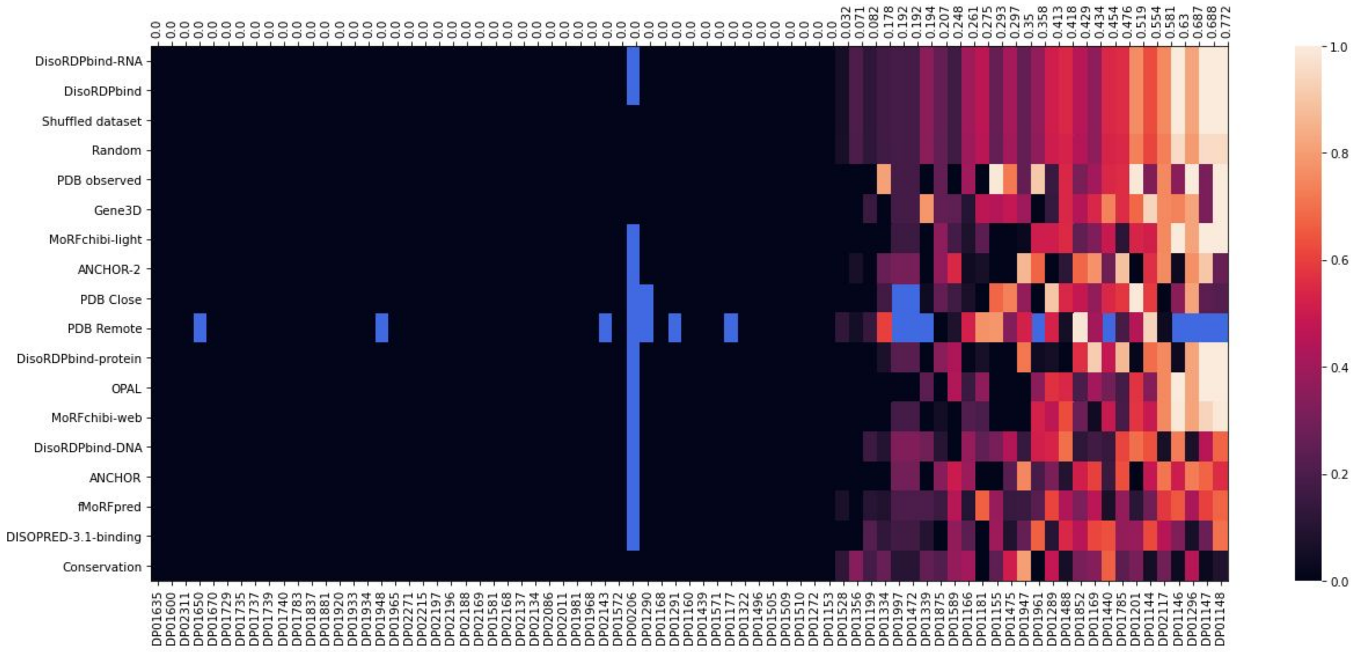

Supplementary Figure 71: F_{Max} per protein for prokaryotic proteins in the *DisProt-Binding* dataset.
F_{Max} of each target (x-axis, bottom labels) from each predictor (y-axis). Targets are sorted by average F_{Max} (x-axis, top labels). Calculated on prokaryotic proteins of the *DisProt-Binding* dataset. Missing values are in blue.

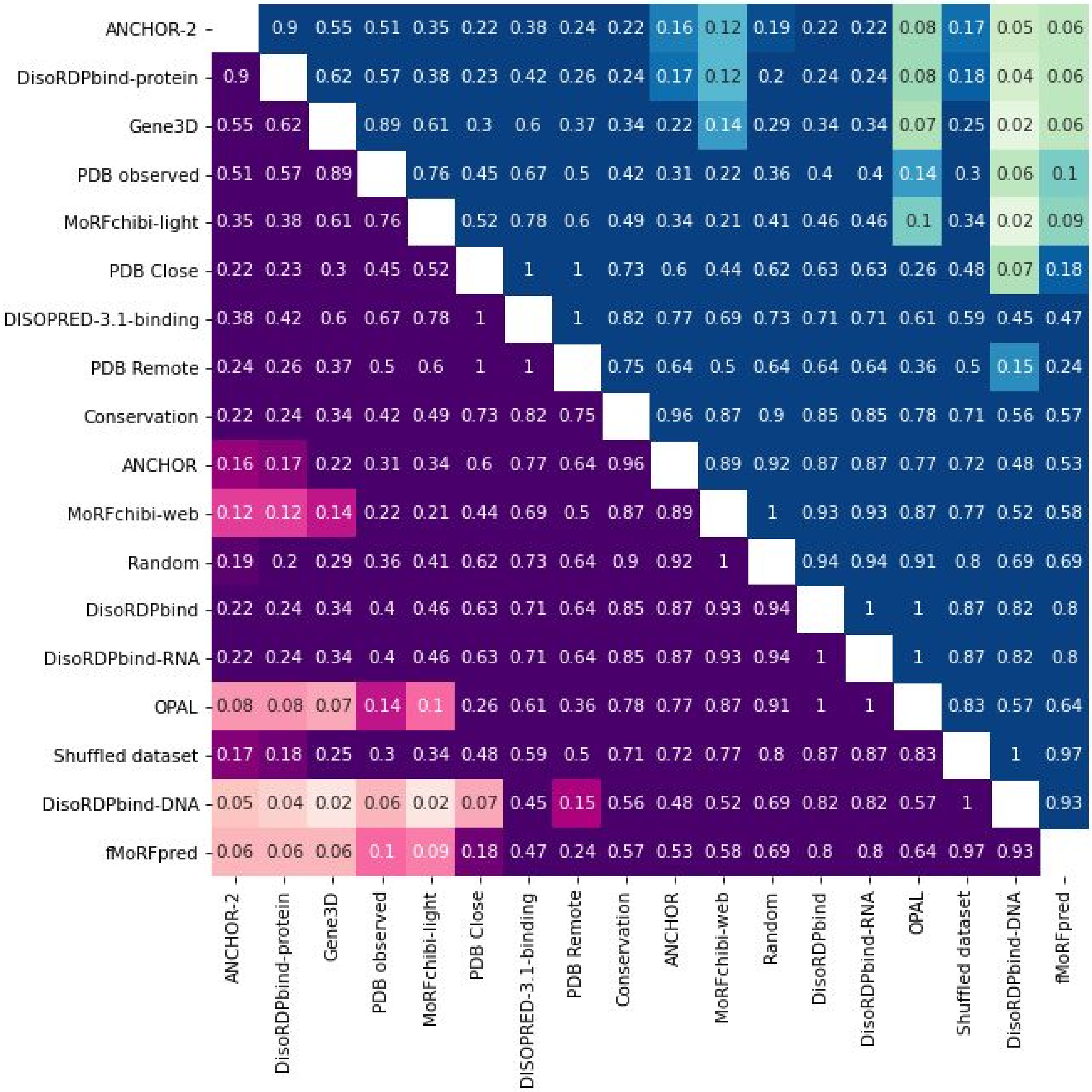

Supplementary Figure 72. Overall average ranking of all predictors and baselines for prokaryotic proteins in the *DisProt-Binding* dataset.
Heatmap of the T-test p-value associated to the statistical significance of the difference between ranking distribution of predictors. A ranking distribution for a predictor is the position of that predictor in its ranking for the following metrics: 'bac', 'f1s', 'fpr', 'mcc', 'ppv', 'tpr', 'tnr'. Metrics are calculated per target and with predictors threshold optimized by F1-Score.
